# Stage-specific associations of nutrition-related surrogate insulin resistance indices across the chronic kidney disease continuum: evidence from a Chinese cohort

**DOI:** 10.3389/fnut.2026.1943087

**Published:** 2026-10-19

**Authors:** Shusheng Fang, Yan Li, Yangxuan He, Jingshan Jiang, Yilin Zhu, Hong Chen, Song Leng

**Affiliations:** Health Management Center, The Second Affiliated Hospital of Dalian Medical University, Dalian, Liaoning, China

**Keywords:** adverse renal outcomes, chronic kidney disease, cohort study, insulin resistance, rapid kidney function decline, risk stratification

## Abstract

**Introduction:**

Comparative longitudinal evidence on how different surrogate insulin resistance (IR) indices relate to chronic kidney disease (CKD) onset and subsequent renal deterioration remains limited. This study aimed to evaluate the associations of surrogate IR indices with CKD incidence, progression, and outcomes.

**Methods:**

This retrospective cohort study included participants from the Dalian Health Management Cohort, in which 12 surrogate IR indices were calculated. The primary outcome was incident CKD, while secondary outcomes included rapid kidney function decline (RKFD) and adverse renal outcomes. Kaplan–Meier analysis compared the CKD incidence across quartiles. Associations between surrogate IR indices and renal outcomes were evaluated using Cox proportional hazards and logistic regression models. Dose–response relationships were assessed using restricted cubic splines, and predictive performance was evaluated using time-dependent receiver operating characteristic curves and associated metrics.

**Results:**

Among 48,304 participants, 1,092 incident CKD cases (2.26%) were identified over a median follow-up of 4.74 years. Kaplan–Meier curves showed increasing CKD risk across the IR quartiles. Quartile-based analyses showed progressively higher incident CKD risk with increasing levels of the evaluated surrogate IR indices (all *P* for trend < 0.001). The triglyceride–glucose index combined with waist circumference (TyG-WC) showed a relatively strong association with incident CKD (Q4 vs. Q1: HR 2.48, 95% CI 1.89–3.26) and exhibited a nonlinear association with an inflection point at 811.58, below which the risk increased more steeply and above which the association tended to plateau. The cholesterol–high-density lipoprotein–glucose index was associated with RKFD (OR per 1-SD increase, 1.56, 95% CI 1.23–1.96), whereas the TyG combined with body mass index was associated with adverse renal outcomes (HR per 1-SD increase, 2.55, 95% CI 1.38–4.71). In predictive analyses, TyG-WC showed the highest discrimination at 1 year (AUC 0.817, 95% CI 0.776–0.851).

**Conclusion:**

Surrogate IR indices were higher in groups with poorer renal outcomes. Indices emphasizing visceral adiposity-related components showed relatively stronger associations with incident CKD, whereas indices incorporating glucose–lipid metabolic and overall adiposity components showed relatively stronger associations with later renal deterioration. TyG-WC may serve as a simple marker for early risk identification and stratification.

## 1 Introduction

Chronic kidney disease (CKD) is a chronic condition characterized by impaired kidney function, defined by a decreased glomerular filtration rate (GFR), presence of kidney damage markers, or both, persisting for at least 3 months, irrespective of the underlying cause (1). CKD is a major global public health issue; as of 2023, the number of individuals with CKD in China has exceeded 150 million, and the disease burden continues to rise (2). However, up to 90% of patients with CKD (including 40% of those with severe CKD) are unaware of their diagnosis, which hinders timely evaluation and optimal disease management (3, 4). Early identification of modifiable metabolic risk factors is therefore crucial for improving CKD prevention and management.

Existing studies suggest that insulin resistance (IR) is associated with CKD, potentially via mechanisms including endothelial dysfunction, reduced bioavailable nitric oxide, activation of the renin–angiotensin–aldosterone system (RAAS), oxidative stress, and inflammation, all of which may contribute to impaired renal function and fibrosis (5, 6). Because direct measurement of IR is difficult in large epidemiological studies, several surrogate IR indices have been developed based on routine anthropometric, lipid, and glucose parameters. These indices capture partially overlapping metabolic characteristics, with different weighting of glucose–lipid metabolism, general adiposity, and visceral adiposity. However, several important gaps remain. First, comparative evidence on how different surrogate IR indices are associated with CKD development and progression remains limited. Second, most previous studies have examined only one or a few surrogate IR indices, limiting direct comparisons of their association patterns and predictive performance (7–9). Third, the definitions of CKD outcomes have varied substantially across studies, including single measurements of estimated glomerular filtration rate (eGFR) < 60 mL/min/1.73 m^2^ (7, 9, 10), a 25% decline in eGFR (8), or annual eGFR decline thresholds such as ≥3 or ≥5 mL/min/1.73 m^2^ (11, 12). Such heterogeneity may compromise comparability and weaken causal interpretation. Fourth, few studies have specifically explored the associations of triglyceride–glucose index (TyG)-related obesity composite indices with the incidence and progression of CKD. Moreover, few studies have examined IR-related indices across the CKD continuum, from incident CKD to subsequent renal deterioration and adverse outcomes.

Therefore, using data from a large Chinese health examination cohort of approximately 50,000 adults, we comprehensively evaluated the associations of 12 surrogate IR indices—TyG, triglyceride-to-high-density lipoprotein cholesterol ratio (TG/HDL-C), metabolic score for insulin resistance (METS-IR), TyG combined with body mass index (TyG-BMI), TyG combined with waist circumference (TyG-WC), TyG combined with waist-to-height ratio (TyG-WHtR), metabolic score for visceral fat (METS-VF), lipid accumulation product (LAP), Chinese visceral adiposity index (CVAI), atherogenic index of plasma (AIP), cholesterol–high-density lipoprotein–glucose index (CHG), and remnant cholesterol (RC)—with incident CKD, CKD progression, and subsequent clinical surrogate endpoints. We further compared their predictive performance to evaluate the relative discriminative ability of different surrogate IR indices for CKD risk.

## 2 Materials and methods

### 2.1 Study design and participants

This study utilized data from the Dalian Health Management Cohort (DHMC; Cohort number: CCC2023112102; https://cohortconsortium.com/queue/management/2669932f46174321b26fa069449e769c?source=queues), a large-scale ongoing cohort initiated in 2014 based on a health examination population from the medical examination center of a tertiary hospital in Dalian. Further details of the cohort design have been published elsewhere (13).

This study was based on a health examination cohort from 2014 to 2025. A total of 69,152 adult participants aged ≥18 years who had undergone at least two routine health examinations were initially screened. Participants were instructed to avoid strenuous exercise, significant psychological stress, and a high-protein diet for 3 days prior to the health examination. Female participants underwent examinations 3 days after the end of menstruation to minimize potential physiological interference. The eGFR was calculated using the 2021 CKD-EPI serum creatinine (Scr)-based equation (14).

The inclusion criteria were: (1) baseline eGFR ≥60 mL/min/1.73 m^2^; and (2) at least two follow-up examinations at intervals ≥3 months. Exclusion criteria were: (1) baseline eGFR <60 mL/min/1.73 m^2^ (*n* = 20,573); (2) evidence of acute infection (white blood cell count (WBC) > 10 × 10^9^/L; *n* = 23); (3) history of tumors, glomerulonephritis, urinary tract infection, hydronephrosis, or polycystic kidney disease (*n* = 237); and (4) follow-up interval <3 months (n = 15). After applying these criteria, a total of 48,304 participants were included in the final baseline cohort (Figure 1). Baseline was defined as the first health examination. Follow-up for CKD incidence was defined as the period from baseline to the first CKD occurrence (confirmed by a second positive result) or the last examination; follow-up for endpoints was defined as the period from CKD diagnosis to the first endpoint occurrence or the last examination.

**Figure 1:**
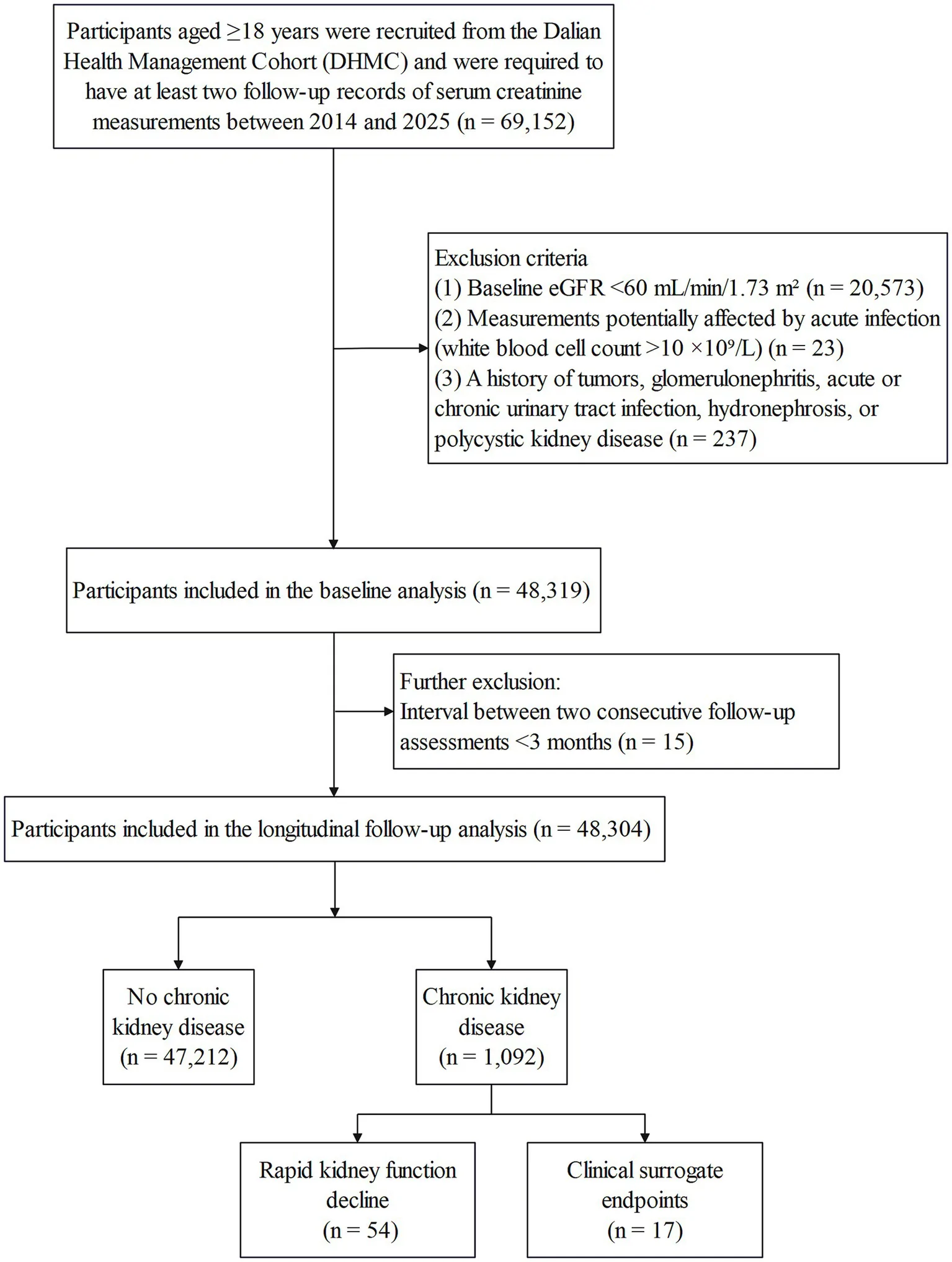
Flowchart of study participants.

### 2.2 Ethics statement

This study involved human participants and was conducted in accordance with the principles of the Declaration of Helsinki (15). The study was approved by the Ethics Review Committee of the Second Affiliated Hospital of Dalian Medical University (approval no.: KY2025-603-01). Considering the retrospective design and the use of anonymized data, the requirement for informed consent was waived by the Ethics Review Committee (16). All patient data were handled in accordance with strict confidentiality and data protection protocols.

### 2.3 Data collection

Participants were instructed to remove their shoes and wear light clothing, and height and weight were measured using standardized instruments. Blood pressure was measured with an electronic sphygmomanometer after ≥5 min of rest. Systolic blood pressure (SBP) and diastolic blood pressure (DBP) were assessed by trained nurses using standardized procedures. Waist circumference (WC) was measured at the midpoint between the lower margin of the rib cage and the iliac crest.

After ≥10 h of overnight fasting, venous blood samples were collected. Biochemical parameters were measured using a Cobas c501 automatic biochemical analyzer (Roche Diagnostics, Germany), including HDL-C, total cholesterol (TC), low-density lipoprotein cholesterol (LDL-C), TG, fasting plasma glucose (FPG), serum uric acid (SUA), urea, Scr, and alanine aminotransferase (ALT). Hematological parameters, including WBC count and differential counts (absolute lymphocytes, neutrophils, and monocytes), red blood cell (RBC) count, hemoglobin, and platelet count, were measured using an automated hematology analyzer. BMI was calculated as weight (kg) divided by height squared (m^2^), and WHtR was calculated as WC (cm) divided by height (cm). The formulas for the surrogate IR indices are provided in the Supplementary methods.

### 2.4 Outcome

Following previous studies, CKD was defined as having two consecutive eGFR values <60 mL/min/1.73 m^2^, with a time interval of at least 3 months between measurements (17). Rapid kidney function decline (RKFD) was defined as an annual eGFR decline ≥5 mL/min/1.73 m^2^, calculated as: (eGFR at CKD diagnosis − eGFR at follow-up) / follow-up duration (12). Clinical surrogate endpoints were defined as a ≥ 40% decline in eGFR from the value at CKD diagnosis (18) or a doubling of Scr (19) from the value at CKD diagnosis.

### 2.5 Covariates

The following baseline covariates were included: age, sex, smoking status, alcohol consumption, medication use, diabetes, dyslipidemia, hypertension, and baseline eGFR. Smoking status was assessed using a single question on ever or current smoking, with responses categorized as ever or never smokers (20). Alcohol consumption was assessed similarly and categorized as ever or never drinkers (20). Medication use at baseline was defined as the use of antihypertensive, lipid-lowering, or antidiabetic drugs (20). Hypertension was defined as SBP ≥ 140 mmHg, DBP ≥ 90 mmHg, current antihypertensive use, or self-reported hypertension (21). Diabetes was defined as antidiabetic medication or insulin use, a history of diabetes, FPG ≥ 7.0 mmol/L, or glycated hemoglobin A1c (HbA1c) ≥ 6.5% (22). Dyslipidemia was defined as TG > 1.7 mmol/L, TC > 5.2 mmol/L, LDL-C > 3.4 mmol/L, or HDL-C ≤ 1.0 mmol/L (23).

### 2.6 Statistical analysis

Continuous variables were assessed for normality using the Anderson–Darling test. As variables were non-normally distributed, they were presented as medians (interquartile range) and compared using the Wilcoxon rank-sum test. Categorical variables were presented as counts (percentages) and compared using the chi-square test. Multiple imputation was performed for missing data using “miceforest,” with the proportion of missing data shown in Supplementary Table 1 (13). Potential confounders were selected based on a directed acyclic graph (DAG) (Supplementary Figure 1).

Kaplan–Meier analysis was used to compare CKD incidence across quartiles of surrogate IR indices, and incidence density was calculated per 1,000 person-years. Associations between surrogate IR indices and CKD incidence were evaluated using multivariable Cox proportional hazards models, with hazard ratios (HRs) and 95% confidence intervals (CIs) reported. Owing to potential collinearity among surrogate IR indices, each index was analyzed separately in the regression models. Pairwise correlations among the 12 surrogate IR indices were additionally assessed using Spearman correlation coefficients to characterize the degree of overlap among the indices and were visualized using a correlation heatmap. Exposures were assessed in quartiles, as continuous variables (per unit increase), and additionally standardized (per standard deviation [per-SD] increase). The proportional hazards assumption was tested using Schoenfeld residuals, with no violations observed (Supplementary Table 2). Three sequential models were constructed: Model 1 was unadjusted; Model 2 was adjusted for age and sex; and Model 3 (fully adjusted model) was further adjusted for smoking status, alcohol consumption, medication use, diabetes, dyslipidemia, hypertension, and eGFR. Logistic regression was used for RKFD, and Cox proportional hazards models were used for surrogate endpoints. In both analyses, Model 1 was unadjusted; Model 2 was adjusted for age and sex; and Model 3 was additionally adjusted for eGFR, which was selected *a priori* to minimize overadjustment.

Dose–response relationships were assessed using restricted cubic splines (RCS) with four knots located at the 5th, 35th, 65th, and 95th percentiles. Segmented Cox regression was applied to evaluate threshold effects, with HRs estimated per-SD increase. Cut points were identified using maximally selected rank statistics and confirmed via the likelihood ratio test (LRT). Subgroup analyses were conducted according to age, sex, smoking status, alcohol consumption, BMI, hypertension, diabetes, and dyslipidemia.

Seven sensitivity analyses were performed. For incident CKD, we repeated the analyses by: (1) excluding participants with >2-year intervals between consecutive eGFR measurements; (2) excluding individuals receiving medications; (3) applying a stricter CKD definition requiring at least two consecutive eGFR values <45 mL/min/1.73 m^2^ measured ≥3 months apart (17); (4) further adjusting for SBP, FPG, and absolute monocyte count; and (5) excluding participants with BMI ≥ 28 kg/m^2^. Among participants who developed incident CKD, we further evaluated the associations between surrogate IR indices and: (6) RKFD after excluding participants with BMI ≥ 28 kg/m^2^; and (7) surrogate endpoints after excluding participants with BMI ≥ 28 kg/m^2^.

Discrimination was assessed using time-dependent receiver operating characteristic (ROC) curves, with area under the curve (AUC) estimated at 1–5 years, along with Harrell’s concordance index (Harrell’s C-index) and Uno’s C-index. Model calibration, clinical utility, and reclassification at 3 years were evaluated using decile-based calibration plots with locally weighted regression smoothing, decision curve analysis (DCA), and continuous net reclassification improvement (NRI) with integrated discrimination improvement (IDI). Prediction error was quantified using the time-dependent Brier score and summarized by the integrated Brier score (IBS). The baseline model included age, sex, smoking status, alcohol consumption, dyslipidemia, hypertension, diabetes, medication use, and baseline eGFR, while the extended model additionally incorporated TyG-WC. Internal validation was performed using bootstrap resampling (1,000 iterations) to obtain optimism-corrected performance estimates. Participants were stratified into high- and low-risk groups based on the median predicted 3-year risk, and Kaplan–Meier curves were used to assess risk discrimination.

Exploratory mediation analyses were conducted to assess whether absolute monocyte count partially mediates the association between high-level (≥75th percentile) values of surrogate IR indices and incident CKD; however, given the observational nature of the study, causal inference should be interpreted cautiously. Indirect effects with 95% confidence intervals were estimated using bootstrap resampling (1,000 iterations).

All statistical analyses were two-sided, with *p* < 0.05 considered statistically significant. Analyses were conducted using R version 4.4.1.

## 3 Results

### 3.1 Baseline characteristics

Among 48,304 participants, 1,092 incident CKD cases (2.26%) were identified over a median follow-up of 4.74 years, yielding an incidence rate of 4.56 per 1,000 person-years. Compared with participants without CKD, those who developed CKD exhibited less favorable cardiorenal metabolic profiles, including older age, a higher proportion of males, higher rates of smoking and alcohol consumption, and a greater comorbidity burden (diabetes, dyslipidemia, and hypertension). They also exhibited less favorable anthropometric and laboratory profiles, with higher SBP, DBP, body weight, WC, BMI, FPG, SUA, Scr, urea, TC, TG, and LDL-C and lower eGFR (all *p* < 0.001, Table 1).

**Table 1:** Baseline characteristics of participants by CKD status.

Variables	No CKD (*n* = 47,212)	CKD (*n* = 1,092)	Overall (*n* = 48,304)	*P*
Age (years)	41.00 (32.00, 51.00)	47.00 (36.00, 56.00)	41.00 (32.00, 51.00)	<0.001
Sex (%)				<0.001
Men	24327.00 (51.53%)	838.00 (76.74%)	25165.00 (52.10%)	
Women	22885.00 (48.47%)	254.00 (23.26%)	23139.00 (47.90%)	
Smoking status (%)	4145.00 (8.78%)	178.00 (16.30%)	4323.00 (8.95%)	<0.001
Alcohol consumption (%)	3824.00 (8.10%)	165.00 (15.11%)	3989.00 (8.26%)	<0.001
Medication use (%)	3783.00 (8.01%)	281.00 (25.73%)	4064.00 (8.41%)	<0.001
Diabetes (%)	2417.00 (5.12%)	201.00 (18.41%)	2618.00 (5.42%)	<0.001
Dyslipidemia (%)	14183.00 (30.04%)	500.00 (45.79%)	14683.00 (30.40%)	<0.001
Hypertension (%)	10417.00 (22.06%)	480.00 (43.96%)	10897.00 (22.56%)	<0.001
SBP (mmHg)	124.00 (114.00, 135.00)	134.00 (124.00, 148.00)	125.00 (115.00, 135.00)	<0.001
DBP (mmHg)	75.00 (69.00, 83.00)	82.00 (74.00, 90.00)	75.00 (69.00, 83.00)	<0.001
Height (cm)	169.00 (163.00, 175.00)	172.00 (166.00, 176.00)	169.00 (163.00, 175.00)	<0.001
Weight (kg)	68.00 (59.00, 78.00)	77.00 (68.00, 87.00)	68.00 (59.00, 78.80)	<0.001
WC (cm)	84.00 (76.00, 91.00)	92.00 (84.00, 98.00)	84.00 (76.00, 91.20)	<0.001
BMI (kg/m^2^)	23.95 (21.61, 26.44)	26.13 (23.81, 28.73)	24.01 (21.63, 26.49)	<0.001
WHtR	0.49 (0.46, 0.53)	0.53 (0.49, 0.57)	0.49 (0.46, 0.53)	<0.001
RBC (10^12^/L)	4.80 (4.48, 5.14)	5.03 (4.71, 5.29)	4.81 (4.48, 5.15)	<0.001
Hemoglobin (g/L)	145.00 (133.00, 157.00)	153.00 (143.00, 161.00)	145.00 (133.00, 157.00)	<0.001
WBC (10^9^/L)	5.94 (5.06, 6.98)	6.51 (5.50, 7.72)	5.96 (5.07, 7.00)	<0.001
Lymphocyte (10^9^/L)	2.00 (1.66, 2.38)	2.10 (1.74, 2.58)	2.00 (1.66, 2.39)	<0.001
Monocyte (10^9^/L)	0.32 (0.25, 0.40)	0.35 (0.27, 0.45)	0.32 (0.25, 0.40)	<0.001
Neutrophil (10^9^/L)	3.40 (2.76, 4.17)	3.76 (3.08, 4.59)	3.41 (2.76, 4.18)	<0.001
Platelets (10^9^/L)	236.00 (204.00, 272.00)	227.00 (196.00, 267.50)	236.00 (203.00, 272.00)	<0.001
ALT (U/L)	19.00 (13.67, 28.40)	24.01 (16.98, 37.85)	19.00 (13.75, 28.65)	<0.001
TC (mmol/L)	4.83 (4.27, 5.46)	5.02 (4.43, 5.66)	4.84 (4.28, 5.47)	<0.001
TG (mmol/L)	1.34 (0.96, 1.91)	1.70 (1.20, 2.45)	1.35 (0.96, 1.92)	<0.001
HDL-C (mmol/L)	1.31 (1.11, 1.54)	1.16 (1.01, 1.38)	1.30 (1.10, 1.54)	<0.001
LDL-C (mmol/L)	2.60 (2.15, 3.11)	2.70 (2.26, 3.19)	2.60 (2.15, 3.11)	<0.001
FPG (mmol/L)	5.43 (5.14, 5.80)	5.80 (5.31, 6.76)	5.44 (5.14, 5.81)	<0.001
SUA (μmol/L)	338.12 (275.94, 409.88)	372.35 (314.88, 440.39)	339.04 (276.57, 410.58)	<0.001
Urea (mmol/L)	4.80 (4.08, 5.61)	5.16 (4.40, 6.09)	4.81 (4.09, 5.62)	<0.001
Scr (μmol/L)	65.59 (55.21, 77.24)	72.05 (62.00, 82.05)	65.82 (55.31, 77.39)	<0.001
eGFR (mL/min/1.73 m^2^)	111.44 (102.78, 119.78)	107.22 (96.76, 115.66)	111.35 (102.67, 119.71)	<0.001
TyG	8.68 (8.31, 9.07)	9.01 (8.63, 9.49)	8.68 (8.32, 9.08)	<0.001
TG/HDL-C	2.32 (1.50, 3.70)	3.38 (2.17, 5.24)	2.34 (1.51, 3.74)	<0.001
METS-IR	35.41 (30.41, 41.09)	41.43 (35.81, 47.52)	35.54 (30.48, 41.25)	<0.001
TyG-BMI	208.37 (182.02, 237.47)	238.63 (208.97, 268.39)	208.97 (182.37, 238.32)	<0.001
TyG-WC	724.81 (635.74, 819.87)	829.87 (742.38, 916.27)	727.13 (637.08, 822.55)	<0.001
TyG-WHtR	4.29 (3.82, 4.80)	4.84 (4.35, 5.34)	4.31 (3.83, 4.81)	<0.001
METS-VF	6.21 (5.54, 6.74)	6.77 (6.31, 7.12)	6.23 (5.56, 6.75)	<0.001
LAP	28.56 (15.96, 49.40)	47.59 (28.08, 76.22)	28.88 (16.12, 49.92)	<0.001
CVAI	78.81 (39.82, 115.89)	120.03 (84.32, 150.15)	79.77 (40.45, 116.93)	<0.001
AIP	0.01 (−0.18, 0.21)	0.17 (−0.02, 0.36)	0.01 (−0.18, 0.21)	<0.001
CHG	5.20 (4.97, 5.44)	5.44 (5.20, 5.69)	5.21 (4.97, 5.45)	<0.001
RC	31.71 (24.75, 41.38)	37.90 (29.20, 51.24)	32.10 (24.75, 41.38)	<0.001

AIP, atherogenic index of plasma; ALT, alanine aminotransferase; BMI, body mass index; CHG, cholesterol–high-density lipoprotein–glucose index; CKD, chronic kidney disease; CVAI, Chinese visceral adiposity index; DBP, diastolic blood pressure; eGFR, estimated glomerular filtration rate; FPG, fasting plasma glucose; HDL-C, high-density lipoprotein cholesterol; LAP, lipid accumulation product; LDL-C, low-density lipoprotein cholesterol; METS-IR, metabolic score for insulin resistance; METS-VF, metabolic score for visceral fat; RBC, red blood cell count; RC, remnant cholesterol; SBP, systolic blood pressure; Scr, serum creatinine; SUA, serum uric acid; TC, total cholesterol; TG, triglycerides; TG/HDL-C, triglyceride-to-high-density lipoprotein cholesterol ratio; TyG, triglyceride–glucose index; TyG-BMI, triglyceride–glucose index combined with body mass index; TyG-WC, triglyceride–glucose index combined with waist circumference; TyG-WHtR, triglyceride–glucose index combined with waist-to-height ratio; WBC, white blood cell count; WC, waist circumference; WHtR, waist-to-height ratio.

Among patients with incident CKD, 54 (4.9%) experienced RKFD and 17 (1.6%) developed surrogate endpoint events. During a median follow-up of 2.8 years, the incidence rate of surrogate endpoint events was 9.83 per 1,000 person-years. Compared with non-progressors, patients progressing to RKFD or surrogate endpoint events exhibited less favorable cardiorenal metabolic profiles and a greater burden of comorbidities, including older age, smoking and alcohol consumption, diabetes, and hypertension. Renal function was poorer among progressors, as indicated by higher Scr, urea, and SUA levels and lower eGFR (all *p* < 0.05, Supplementary Tables 3, 4). In addition, surrogate IR indices (TyG, TG/HDL-C, METS-IR, TyG-BMI, TyG-WC, TyG-WHtR, METS-VF, LAP, CVAI, AIP, CHG, RC) were generally higher in groups with poorer renal outcomes, suggesting greater metabolic dysfunction across the evaluated outcome groups (Table 1 and Supplementary Tables 3, 4).

Pairwise analyses showed moderate-to-strong positive correlations among the 12 surrogate IR indices (Spearman ρ, 0.36–1.00; Supplementary Figure 2). Correlations were stronger among indices sharing metabolic or anthropometric components, particularly TyG-related adiposity indices, whereas RC was more weakly correlated with several measures. Notably, TG/HDL-C and AIP showed a perfect rank correlation, consistent with their closely related mathematical formulations. These findings indicate substantial but variable overlap in metabolic information across the surrogate indices.

### 3.2 Association between surrogate IR indices and incident CKD

Kaplan–Meier curves showed a graded increase in CKD risk across the IR quartiles, with the highest quartile showing the greatest risk (Figure 2). In fully adjusted Cox models, quartile-based analyses showed progressively higher incident CKD risk with increasing levels of the surrogate IR indices (all *P* for trend < 0.001). Among the indices, TyG-WC, METS-IR, and CVAI showed relatively stronger associations with CKD risk (HR 2.48, 95% CI 1.89–3.26; HR 2.41, 95% CI 1.89–3.08; and HR 2.39, 95% CI 1.79–3.18, respectively; all *p* < 0.001; Table 2).

**Figure 2:**
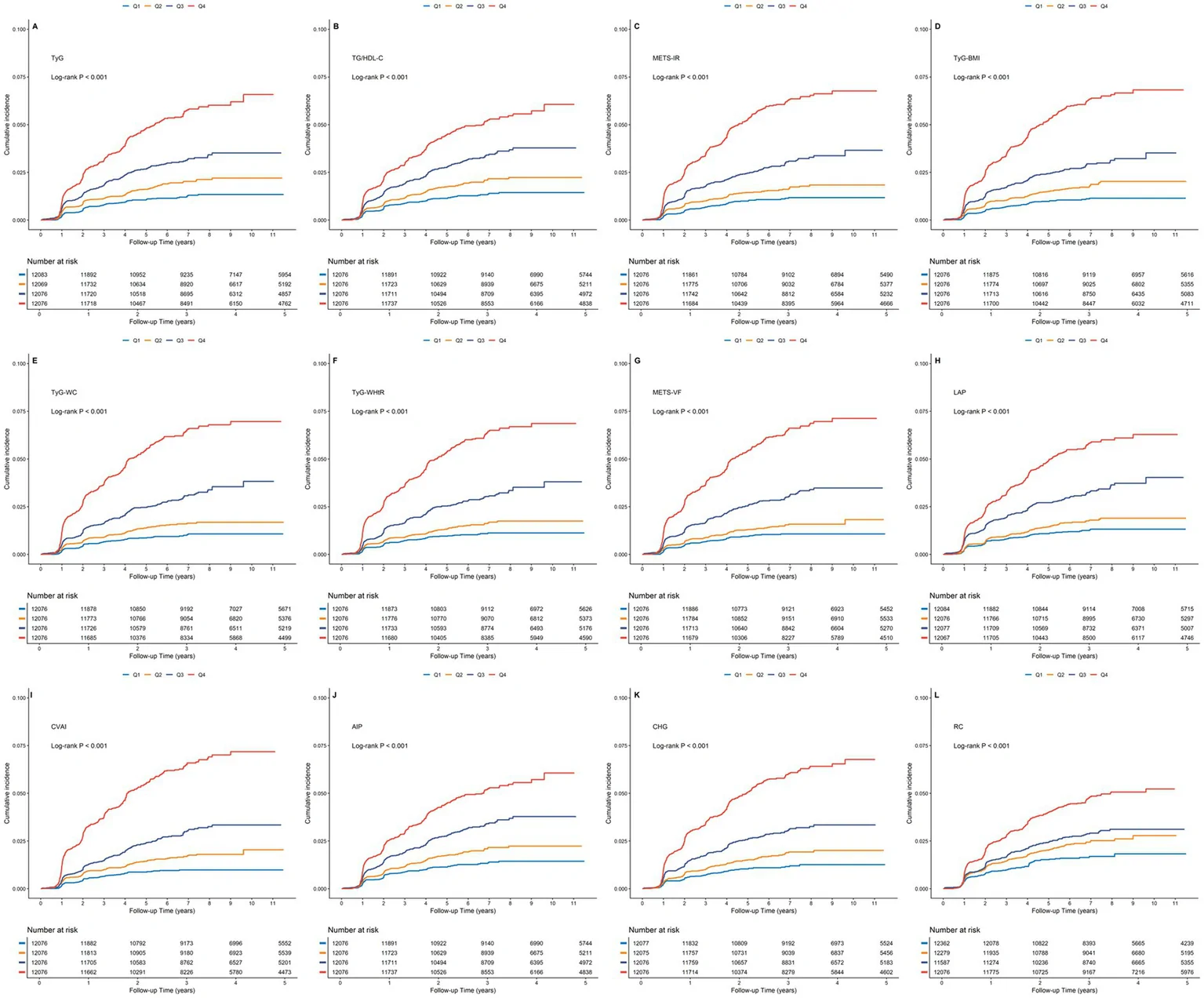
Kaplan–Meier curves for cumulative incidence of CKD across quartiles of surrogate indices of insulin resistance: **(A)** TyG; **(B)** TG/HDL-C; **(C)** METS-IR; **(D)** TyG-BMI; **(E)** TyG-WC; **(F)** TyG-WHtR; **(G)** METS-VF; **(H)** LAP; **(I)** CVAI; **(J)** AIP; **(K)** CHG; **(L)** RC. Abbreviations: AIP, atherogenic index of plasma; CHG, cholesterol–high-density lipoprotein–glucose index; CKD, chronic kidney disease; CVAI, Chinese visceral adiposity index; LAP, lipid accumulation product; METS-IR, metabolic score for insulin resistance; METS-VF, metabolic score for visceral fat; RC, remnant cholesterol; TG/HDL-C, triglyceride to high-density lipoprotein cholesterol ratio; TyG, triglyceride–glucose index; TyG-BMI, triglyceride–glucose index combined with body mass index; TyG-WC, triglyceride–glucose index combined with waist circumference; TyG-WHtR, triglyceride–glucose index combined with waist-to-height ratio.

**Table 2:** Associations of surrogate indices of insulin resistance with incident CKD.

Exposure	Levels	Events	PY	Incidence Rate	Model 1		Model 2		Model 3	
					HR (95% CI)	*P*	HR (95% CI)	*P*	HR (95% CI)	*P*
TyG	Continuous				2.33 (2.16–2.52)	<0.001	1.88 (1.72–2.05)	<0.001	1.63 (1.45–1.84)	<0.001
	Per 1-SD				1.65 (1.58–1.73)	<0.001	1.45 (1.38–1.53)	<0.001	1.34 (1.25–1.44)	<0.001
	Q1	121	61683.211	1.962	Reference	–	Reference	–	Reference	–
	Q2	181	57366.519	3.155	1.57 (1.25–1.98)	<0.001	1.26 (1.00–1.58)	0.054	1.21 (0.96–1.52)	0.113
	Q3	287	55555.479	5.166	2.54 (2.05–3.14)	<0.001	1.75 (1.41–2.17)	<0.001	1.60 (1.29–1.99)	<0.001
	Q4	503	55156.425	9.12	4.48 (3.68–5.47)	<0.001	2.62 (2.13–3.23)	<0.001	1.99 (1.57–2.53)	<0.001
	*P* for trend					<0.001		<0.001		<0.001
TG/HDL-C	Continuous				1.00 (1.00–1.00)	0.028	1.00 (1.00–1.00)	0.193	1.00 (1.00–1.00)	0.602
	Per 1-SD				1.02 (1.00–1.04)	0.028	1.02 (0.99–1.04)	0.193	1.01 (0.97–1.05)	0.602
	Q1	131	60539.696	2.164	Reference	–	Reference	–	Reference	–
	Q2	188	57422.139	3.274	1.49 (1.19–1.86)	<0.001	1.20 (0.96–1.50)	0.118	1.14 (0.91–1.42)	0.259
	Q3	303	56073.774	5.404	2.44 (1.99–2.99)	<0.001	1.65 (1.34–2.04)	<0.001	1.50 (1.21–1.85)	<0.001
	Q4	470	55726.024	8.434	3.80 (3.13–4.62)	<0.001	2.26 (1.85–2.77)	<0.001	1.92 (1.51–2.43)	<0.001
	*P* for trend					<0.001		<0.001		<0.001
METS-IR	Continuous				1.00 (1.00–1.00)	<0.001	1.00 (1.00–1.00)	<0.001	1.00 (1.00–1.00)	<0.001
	Per 1-SD				1.03 (1.02–1.04)	<0.001	1.03 (1.02–1.05)	<0.001	1.03 (1.02–1.05)	<0.001
	Q1	106	58801.937	1.803	Reference	–	Reference	–	Reference	–
	Q2	157	58414.936	2.688	1.49 (1.17–1.91)	0.001	1.10 (0.86–1.41)	0.452	1.09 (0.85–1.40)	0.509
	Q3	278	57653.956	4.822	2.67 (2.14–3.34)	<0.001	1.59 (1.25–2.01)	<0.001	1.46 (1.14–1.86)	0.002
	Q4	551	54890.804	10.038	5.50 (4.47–6.77)	<0.001	3.04 (2.43–3.82)	<0.001	2.41 (1.89–3.08)	<0.001
	*P* for trend					<0.001		<0.001		<0.001
TyG-BMI	Continuous				1.00 (1.00–1.00)	<0.001	1.00 (1.00–1.00)	<0.001	1.00 (1.00–1.00)	<0.001
	Per 1-SD				1.02 (1.02–1.03)	<0.001	1.03 (1.02–1.04)	<0.001	1.03 (1.01–1.04)	<0.001
	Q1	106	59706.722	1.775	Reference	–	Reference	–	Reference	–
	Q2	167	58248.746	2.867	1.60 (1.26–2.05)	<0.001	1.16 (0.91–1.49)	0.237	1.14 (0.89–1.46)	0.306
	Q3	267	56831.356	4.698	2.61 (2.09–3.27)	<0.001	1.52 (1.20–1.92)	<0.001	1.38 (1.09–1.76)	0.009
	Q4	552	54974.809	10.041	5.54 (4.50–6.82)	<0.001	3.00 (2.40–3.75)	<0.001	2.29 (1.80–2.92)	<0.001
	*P* for trend					<0.001		<0.001		<0.001
TyG-WC	Continuous				1.00 (1.00–1.00)	<0.001	1.00 (1.00–1.00)	<0.001	1.00 (1.00–1.00)	<0.001
	Per 1-SD				1.15 (1.14–1.17)	<0.001	1.16 (1.14–1.17)	<0.001	1.13 (1.10–1.15)	<0.001
	Q1	97	59901.645	1.619	Reference	–	Reference	–	Reference	–
	Q2	148	58437.014	2.533	1.55 (1.20–2.01)	<0.001	1.14 (0.88–1.49)	0.329	1.13 (0.87–1.48)	0.352
	Q3	274	57334.262	4.779	2.93 (2.32–3.69)	<0.001	1.68 (1.30–2.17)	<0.001	1.55 (1.19–2.02)	0.001
	Q4	573	54088.711	10.594	6.37 (5.14–7.90)	<0.001	3.27 (2.54–4.21)	<0.001	2.48 (1.89–3.26)	<0.001
	*P* for trend					<0.001		<0.001		<0.001
TyG-WHtR	Continuous				1.17 (1.15–1.18)	<0.001	1.18 (1.15–1.20)	<0.001	1.15 (1.12–1.18)	<0.001
	Per 1-SD				1.12 (1.11–1.13)	<0.001	1.13 (1.11–1.14)	<0.001	1.11 (1.09–1.13)	<0.001
	Q1	105	59784.415	1.756	Reference	–	Reference	–	Reference	–
	Q2	148	58387.201	2.535	1.43 (1.11–1.84)	0.005	1.06 (0.82–1.37)	0.652	1.07 (0.83–1.38)	0.609
	Q3	276	57143.351	4.830	2.72 (2.17–3.41)	<0.001	1.61 (1.27–2.05)	<0.001	1.53 (1.20–1.96)	<0.001
	Q4	563	54446.666	10.340	5.74 (4.66–7.07)	<0.001	3.01 (2.39–3.79)	<0.001	2.38 (1.85–3.06)	<0.001
	*P* for trend					<0.001		<0.001		<0.001
METS-VF	Continuous				1.41 (1.37–1.44)	<0.001	1.38 (1.32–1.44)	<0.001	1.33 (1.26–1.41)	<0.001
	Per 1-SD				1.34 (1.31–1.36)	<0.001	1.31 (1.27–1.36)	<0.001	1.28 (1.22–1.34)	<0.001
	Q1	101	58582.745	1.724	Reference	–	Reference	–	Reference	–
	Q2	142	59368.315	2.392	1.40 (1.08–1.81)	0.01	1.10 (0.85–1.43)	0.467	1.10 (0.84–1.43)	0.496
	Q3	280	57858.447	4.839	2.82 (2.25–3.54)	<0.001	1.73 (1.34–2.23)	<0.001	1.57 (1.21–2.05)	<0.001
	Q4	569	53952.126	10.546	6.03 (4.88–7.45)	<0.001	3.05 (2.34–3.99)	<0.001	2.27 (1.71–3.01)	<0.001
	*P* for trend					<0.001		<0.001		<0.001
LAP	Continuous				1.00 (1.00–1.00)	<0.001	1.00 (1.00–1.00)	<0.001	1.00 (1.00–1.00)	<0.001
	Per 1-SD				1.13 (1.11–1.15)	<0.001	1.12 (1.09–1.14)	<0.001	1.08 (1.05–1.11)	<0.001
	Q1	122	60398.757	2.020	Reference	–	Reference	–	Reference	–
	Q2	158	57893.788	2.729	1.33 (1.05–1.68)	0.018	1.01 (0.79–1.28)	0.956	0.98 (0.77–1.24)	0.842
	Q3	301	56372.536	5.339	2.59 (2.10–3.19)	<0.001	1.62 (1.30–2.01)	<0.001	1.48 (1.18–1.84)	<0.001
	Q4	511	55096.552	9.275	4.46 (3.66–5.44)	<0.001	2.48 (2.01–3.06)	<0.001	2.04 (1.61–2.58)	<0.001
	*P* for trend					<0.001		<0.001		<0.001
CVAI	Continuous				1.00 (1.00–1.00)	<0.001	1.00 (1.00–1.00)	<0.001	1.00 (1.00–1.00)	<0.001
	Per 1-SD				1.04 (1.04–1.05)	<0.001	1.05 (1.03–1.06)	<0.001	1.04 (1.03–1.06)	<0.001
	Q1	95	59173.931	1.605	Reference	–	Reference	–	Reference	–
	Q2	160	59387.194	2.694	1.68 (1.31–2.17)	<0.001	1.22 (0.94–1.59)	0.142	1.24 (0.95–1.61)	0.117
	Q3	259	57492.186	4.505	2.81 (2.22–3.55)	<0.001	1.55 (1.18–2.02)	0.002	1.43 (1.08–1.88)	0.012
	Q4	578	53708.322	10.762	6.57 (5.29–8.16)	<0.001	3.10 (2.37–4.07)	<0.001	2.39 (1.79–3.18)	<0.001
	*P* for trend					<0.001		<0.001		<0.001
AIP	Continuous				3.88 (3.34–4.50)	<0.001	2.61 (2.19–3.12)	<0.001	2.26 (1.79–2.86)	<0.001
	Per 1-SD				1.50 (1.44–1.57)	<0.001	1.34 (1.27–1.41)	<0.001	1.28 (1.19–1.37)	<0.001
	Q1	131	60539.696	2.164	Reference	–	Reference	–	Reference	–
	Q2	188	57422.139	3.274	1.49 (1.19–1.86)	<0.001	1.20 (0.96–1.50)	0.118	1.14 (0.91–1.42)	0.259
	Q3	303	56073.774	5.404	2.44 (1.99–2.99)	<0.001	1.65 (1.34–2.04)	<0.001	1.50 (1.21–1.85)	<0.001
	Q4	470	55726.024	8.434	3.80 (3.13–4.62)	<0.001	2.26 (1.85–2.77)	<0.001	1.92 (1.51–2.43)	<0.001
	*P* for trend					<0.001		<0.001		<0.001
CHG	Continuous				3.36 (3.09–3.66)	<0.001	2.74 (2.45–3.06)	<0.001	2.41 (2.07–2.79)	<0.001
	Per 1-SD				1.57 (1.52–1.62)	<0.001	1.46 (1.40–1.52)	<0.001	1.39 (1.31–1.47)	<0.001
	Q1	112	58607.06	1.911	Reference	–	Reference	–	Reference	–
	Q2	168	58654.855	2.864	1.51 (1.19–1.92)	<0.001	1.11 (0.87–1.42)	0.384	1.13 (0.89–1.45)	0.313
	Q3	279	57569.362	4.846	2.54 (2.04–3.17)	<0.001	1.53 (1.21–1.92)	<0.001	1.49 (1.18–1.89)	<0.001
	Q4	533	54930.356	9.703	5.06 (4.12–6.20)	<0.001	2.62 (2.10–3.27)	<0.001	2.15 (1.68–2.76)	<0.001
	*P* for trend					<0.001		<0.001		<0.001
RC	Continuous				1.01 (1.01–1.01)	<0.001	1.01 (1.01–1.01)	<0.001	1.01 (1.00–1.01)	<0.001
	Per 1-SD				1.20 (1.17–1.22)	<0.001	1.17 (1.13–1.21)	<0.001	1.13 (1.09–1.18)	<0.001
	Q1	152	53531.621	2.839	Reference	–	Reference	–	Reference	–
	Q2	228	57889.543	3.939	1.44 (1.17–1.77)	<0.001	1.32 (1.08–1.62)	0.008	1.39 (1.13–1.70)	0.002
	Q3	260	56941.848	4.566	1.70 (1.39–2.08)	<0.001	1.38 (1.13–1.69)	0.002	1.39 (1.14–1.70)	0.001
	Q4	452	61398.621	7.362	2.79 (2.32–3.35)	<0.001	2.00 (1.66–2.41)	<0.001	1.84 (1.50–2.25)	<0.001
	*P* for trend					<0.001		<0.001		<0.001

Incidence rates are reported per 1,000 person-years. Model 1: unadjusted; Model 2: adjusted for age and sex; Model 3: additionally adjusted for smoking status, alcohol consumption, medication use, diabetes, dyslipidemia, hypertension, and eGFR.

AIP, atherogenic index of plasma; CHG, cholesterol–high-density lipoprotein–glucose index; CI, confidence interval; CKD, chronic kidney disease; CVAI, Chinese visceral adiposity index; eGFR, estimated glomerular filtration rate; HR, hazard ratio; LAP, lipid accumulation product; METS-IR, metabolic score for insulin resistance; METS-VF, metabolic score for visceral fat; PY, person-years; RC, remnant cholesterol; SD, standard deviation; TG/HDL-C, triglyceride to high-density lipoprotein cholesterol ratio; TyG, triglyceride–glucose index; TyG-BMI, triglyceride–glucose index combined with body mass index; TyG-WC, triglyceride–glucose index combined with waist circumference; TyG-WHtR, triglyceride–glucose index combined with waist-to-height ratio.

The RCS analyses showed that, except for TyG and CHG, all indices demonstrated significant nonlinear associations with CKD risk (*P* for non-linearity < 0.05) (Figure 3). A significant threshold effect was observed for TyG-WC (*P* for LRT < 0.001), with an inflection point at 811.58. Below the threshold, each 1-SD increase in TyG-WC was associated with higher CKD risk (HR 1.71, 95% CI 1.47–1.98, *p* < 0.001), whereas above the threshold, the association remained significant but was attenuated (HR 1.10, 95% CI 1.07–1.13, *p* < 0.001; Supplementary Table 5).

**Figure 3:**
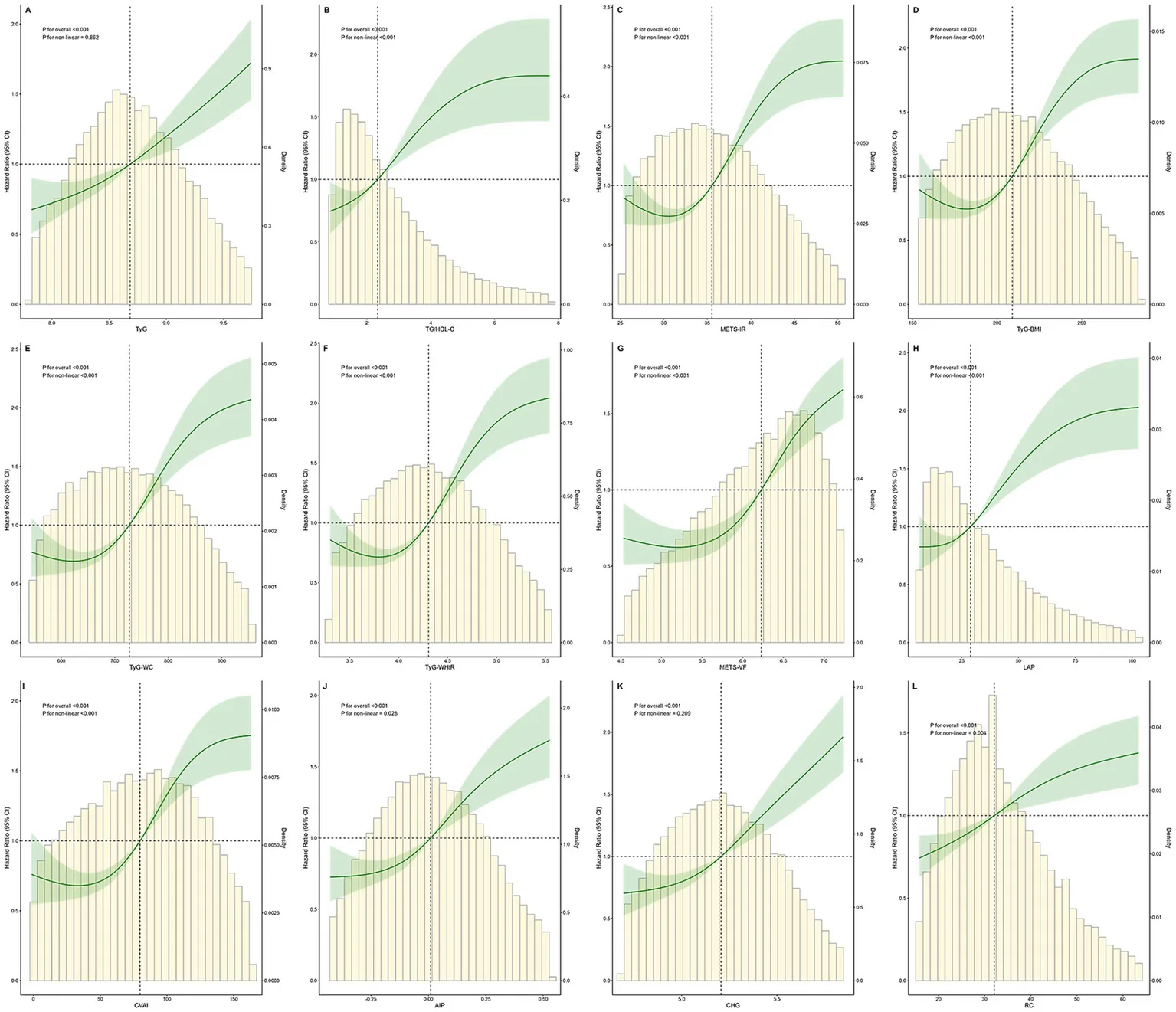
Restricted cubic spline analyses of associations between surrogate insulin resistance indices and incident CKD: **(A)** TyG; **(B) **TG/HDL-C; **(C)** METS-IR; **(D)** TyG-BMI; **(E)** TyG-WC; **(F)** TyG-WHtR; **(G)** METS-VF; **(H)** LAP; **(I)** CVAI; **(J)** AIP; **(K)** CHG; **(L)** RC. Restricted cubic spline models were adjusted for age, sex, smoking status, alcohol consumption, medication use, diabetes, dyslipidemia, hypertension, and eGFR, with all surrogate indices of insulin resistance standardized before modeling. AIP, atherogenic index of plasma; CHG, cholesterol–high-density lipoprotein–glucose index; CI, confidence interval; CKD, chronic kidney disease; CVAI, Chinese visceral adiposity index; eGFR, estimated glomerular filtration rate; HR, hazard ratio; LAP, lipid accumulation product; METS-IR, metabolic score for insulin resistance; METS-VF, metabolic score for visceral fat; RC, remnant cholesterol; TG/HDL-C, triglyceride to high-density lipoprotein cholesterol ratio; TyG, triglyceride–glucose index; TyG-BMI, triglyceride–glucose index combined with body mass index; TyG-WC, triglyceride–glucose index combined with waist circumference; TyG-WHtR, triglyceride–glucose index combined with waist-to-height ratio.

### 3.3 Association between surrogate IR indices and CKD progression

In fully adjusted models, CHG and TyG were significantly associated with RKFD, with ORs per 1-SD increase of 1.56 (95% CI: 1.23–1.96; *p* < 0.001) and 1.46 (95% CI: 1.12–1.90; *p* = 0.006), respectively (Supplementary Table 6). For the surrogate endpoints, TyG-BMI, TyG-WHtR, METS-IR, TyG-WC, CHG, and TyG were significantly associated with increased risk, with HRs per 1-SD increase of 2.55 (95% CI: 1.38–4.71; *p* = 0.003), 2.37 (95% CI: 1.30–4.32; *p* = 0.005), 2.36 (95% CI: 1.44–3.86; *p* < 0.001), 2.24 (95% CI: 1.20–4.18; *p* = 0.011), 1.94 (95% CI: 1.44–2.60; *p* < 0.001), and 1.70 (95% CI: 1.16–2.48; *p* = 0.007), respectively (Supplementary Table 7). RCS analyses demonstrated significant overall associations of TyG, TyG-WC, TyG-WHtR, and CHG with RKFD, and of METS-IR, TyG-WHtR, METS-VF, and CHG with surrogate endpoints (all *P* for overall association < 0.05). Most associations were approximately linear, except for TyG-WC and TyG-WHtR (RKFD) and METS-VF (surrogate endpoints) (Supplementary Figures 3, 4).

### 3.4 Subgroup analyses

Subgroup analyses showed that the association between TyG-WC and incident CKD was consistent across all prespecified subgroups, with no evidence of significant interactions (all *P* for interaction > 0.05). Participants in the highest TyG-WC quartile consistently exhibited markedly higher risk of CKD across all strata (Supplementary Figure 5).

### 3.5 Sensitivity analyses

Sensitivity analyses generally supported the robustness of the primary findings. After excluding participants with intervals >2 years between eGFR measurements, the association between TyG-WC and incident CKD was strengthened (Q4 vs. Q1: HR 3.44, 95% CI 2.42–4.88). Similar results were observed using a more stringent CKD definition (HR 3.04, 95% CI 1.66–5.39). Findings were also largely unchanged after excluding participants receiving medications and after further adjustment for SBP, FPG, and absolute monocyte count (Supplementary Tables 8–11). After excluding participants with BMI ≥ 28 kg/m^2^, associations with incident CKD generally persisted, although several effect estimates were attenuated; TyG-WC remained significantly associated with incident CKD (Q4 vs. Q1: adjusted HR 1.61, 95% CI 1.19–2.19; Supplementary Table 12). CHG and TyG also remained associated with RKFD (per-SD OR 1.63 and 1.50, respectively; Supplementary Table 13). For surrogate endpoints, several surrogate IR indices showed relatively large unadjusted effect estimates, although only eight events occurred (Supplementary Table 14).

### 3.6 Predictive performance

Discrimination performance was comparable across indices, with time-dependent AUCs ranging from 0.737 to 0.817 over 1–5 years (Figure 4 and Table 3). Among them, TyG-WC achieved the highest AUC at 1 year (0.817, 95% CI 0.776–0.851), with only modest differences thereafter. Harrell’s C-index and Uno’s C-index were similar across models, ranging from 0.730 to 0.750 and 0.722 to 0.730, respectively (Supplementary Table 15). Calibration showed generally close agreement between predicted and observed risks (Supplementary Figure 6), and DCA suggested potential clinical utility (Supplementary Figure 7). At 3 years, adding TyG-WC to the baseline model improved risk reclassification, as indicated by a positive continuous net reclassification improvement (NRI = 0.192, 95% CI 0.072–0.318, *p* = 0.012), with a small increase in discrimination (IDI = 0.001, *p* < 0.001). Prediction error was similar across models (IBS: 0.00723–0.00728; Supplementary Figure 8 and Supplementary Table 15). Internal validation using bootstrap resampling yielded similar optimism-corrected estimates. Kaplan–Meier curves for TyG-WC further demonstrated distinct risk stratification, with a higher cumulative incidence of CKD in the high-risk group (log-rank *p* < 0.001; Supplementary Figure 9).

**Figure 4:**
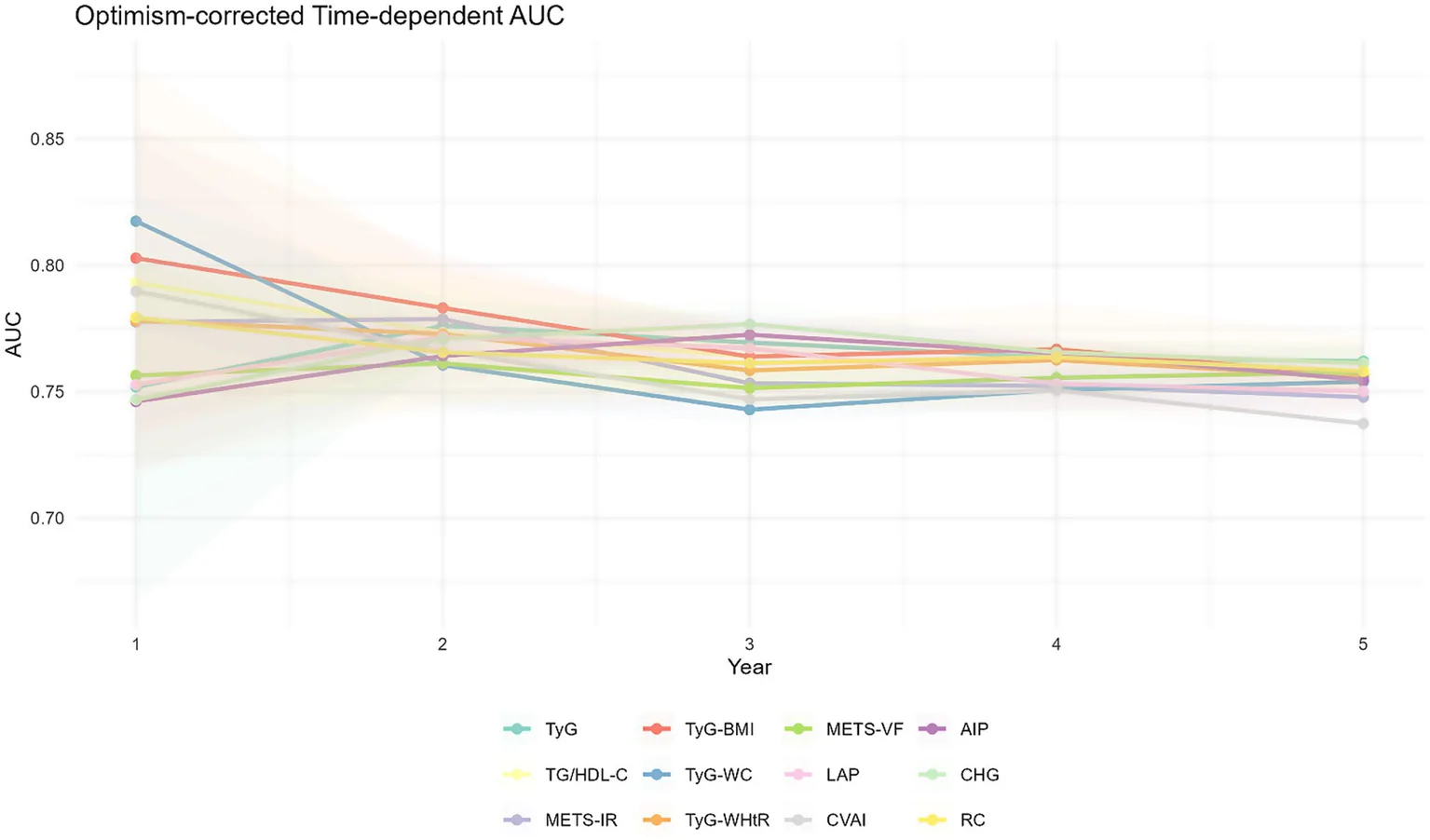
Time-dependent receiver operating characteristic curves for surrogate indices of insulin resistance in predicting incident CKD. Models were adjusted for age, sex, smoking status, alcohol consumption, medication use, diabetes, dyslipidemia, hypertension, and eGFR. AIP, atherogenic index of plasma; AUC, area under the curve; CHG, cholesterol–high-density lipoprotein–glucose index; CKD, chronic kidney disease; CVAI, Chinese visceral adiposity index; eGFR, estimated glomerular filtration rate; LAP, lipid accumulation product; METS-IR, metabolic score for insulin resistance; METS-VF, metabolic score for visceral fat; RC, remnant cholesterol; TG/HDL-C, triglyceride to high-density lipoprotein cholesterol ratio; TyG, triglyceride–glucose index; TyG-BMI, triglyceride–glucose index combined with body mass index; TyG-WC, triglyceride–glucose index combined with waist circumference; TyG-WHtR, triglyceride–glucose index combined with waist-to-height ratio.

**Table 3:** Time-dependent AUCs of surrogate indices of insulin resistance for incident CKD over 1–5 years.

Index	1 ~ year	2 ~ years	3 ~ years	4 ~ years	5 ~ years
TyG	0.752 (0.667–0.827)	0.776 (0.765–0.787)	0.769 (0.753–0.779)	0.764 (0.753–0.770)	0.762 (0.751–0.769)
TG/HDL-C	0.793 (0.749–0.820)	0.773 (0.750–0.799)	0.764 (0.749–0.778)	0.767 (0.765–0.769)	0.757 (0.753–0.759)
METS-IR	0.778 (0.738–0.804)	0.779 (0.767–0.786)	0.753 (0.750–0.758)	0.752 (0.747–0.759)	0.748 (0.742–0.752)
TyG-BMI	0.803 (0.734–0.855)	0.783 (0.763–0.804)	0.764 (0.756–0.774)	0.767 (0.762–0.770)	0.756 (0.752–0.762)
TyG-WC	0.817 (0.776–0.851)	0.761 (0.743–0.771)	0.743 (0.738–0.746)	0.751 (0.744–0.759)	0.754 (0.745–0.769)
TyG-WHtR	0.778 (0.719–0.878)	0.773 (0.745–0.799)	0.758 (0.745–0.777)	0.763 (0.742–0.785)	0.756 (0.742–0.772)
METS-VF	0.756 (0.723–0.802)	0.761 (0.759–0.764)	0.751 (0.742–0.761)	0.756 (0.747–0.763)	0.758 (0.752–0.763)
LAP	0.753 (0.732–0.779)	0.772 (0.766–0.778)	0.767 (0.760–0.778)	0.753 (0.743–0.765)	0.750 (0.741–0.757)
CVAI	0.790 (0.763–0.815)	0.766 (0.740–0.784)	0.747 (0.745–0.750)	0.751 (0.741–0.757)	0.737 (0.734–0.741)
AIP	0.746 (0.719–0.778)	0.764 (0.754–0.781)	0.773 (0.765–0.781)	0.765 (0.758–0.772)	0.755 (0.751–0.758)
CHG	0.747 (0.698–0.780)	0.771 (0.761–0.781)	0.777 (0.769–0.787)	0.766 (0.764–0.767)	0.761 (0.758–0.766)
RC	0.779 (0.746–0.799)	0.766 (0.744–0.794)	0.761 (0.752–0.769)	0.764 (0.756–0.770)	0.758 (0.749–0.769)

Models were adjusted for age, sex, smoking status, alcohol consumption, medication use, diabetes, dyslipidemia, hypertension, and eGFR.

AIP, atherogenic index of plasma; AUC, area under the curve; CHG, cholesterol–high-density lipoprotein–glucose index; CKD, chronic kidney disease; CVAI, Chinese visceral adiposity index; eGFR, estimated glomerular filtration rate; LAP, lipid accumulation product; METS-IR, metabolic score for insulin resistance; METS-VF, metabolic score for visceral fat; RC, remnant cholesterol; TG/HDL-C, triglyceride to high-density lipoprotein cholesterol ratio; TyG, triglyceride–glucose index; TyG-BMI, triglyceride–glucose index combined with body mass index; TyG-WC, triglyceride–glucose index combined with waist circumference; TyG-WHtR, triglyceride–glucose index combined with waist-to-height ratio.

### 3.7 Mediation analysis

Exploratory mediation analyses showed small but statistically significant indirect effects through absolute monocyte count, with estimated mediation proportions ranging from 3.64 to 5.23%. For TyG-WC, the estimated mediation proportion was 4.76% (95% CI 3.5–7.8%) (Supplementary Figure 10).

## 4 Discussion

In this large cohort of nearly 50,000 participants, surrogate IR indices were generally higher in groups with poorer renal outcomes, and quartile-based analyses showed progressively higher incident CKD risk with increasing levels of the evaluated indices. Beyond this overall association, the relationships between surrogate IR indices and subsequent renal deterioration varied across the evaluated renal outcomes. TyG-WC was relatively strongly and robustly associated with incident CKD, whereas CHG and TyG-BMI were more strongly associated with RKFD and adverse renal outcomes, respectively. These findings suggest that different surrogate IR indices may place varying emphasis on shared metabolic components across CKD onset and subsequent renal deterioration; however, these outcome-specific patterns should be considered exploratory and require further validation. Given the substantial correlations among surrogate IR indices, these measures should not be interpreted as independent biomarkers. Rather, differences in their formulations may place varying emphasis on shared metabolic and anthropometric components, potentially explaining the observed heterogeneity in associations across the CKD continuum. Among the evaluated indices, TyG-WC demonstrated a modest advantage in short-term risk prediction and risk stratification. Exploratory mediation analysis further suggested a small indirect effect through monocyte count in the association between TyG-WC and incident CKD.

Beyond the overall association between TyG-WC and incident CKD, threshold analysis identified an inflection point at 811.58, below which the risk of incident CKD increased more rapidly, whereas the association remained significant but attenuated above this threshold. This nonlinear pattern may reflect a dynamic relationship between central adiposity-related IR and renal susceptibility, whereby early increases in metabolic burden may induce disproportionate renal stress through hemodynamic alterations and glomerular hyperfiltration, whereas further increases may have smaller incremental effects once these pathways are substantially established (5, 6, 24). In addition, subclinical kidney dysfunction could itself influence metabolic indices, potentially contributing to attenuation of the exposure–risk gradient at higher TyG-WC levels. However, this inflection point should be interpreted as a statistical threshold rather than a biological cutoff, and both its biological basis and potential susceptibility to reverse causation require further investigation.

The stage-specific patterns of surrogate IR indices may arise from differences in how these correlated measures integrate glucose–lipid metabolism and adiposity-related information. TyG-WC integrates TG, FPG, and WC and may reflect visceral adiposity and early IR (25, 26). Visceral adiposity has been linked to nephron injury-related processes, including chronic inflammation, oxidative stress, and lipotoxicity (27), which may contribute to incident CKD through increased metabolic and hemodynamic stress. During CKD development, increased metabolic burden may induce glomerular hypertension and hyperfiltration, which are initially compensated by nephron hypertrophy and increased single-nephron filtration; however, persistent hyperfiltration may increase podocyte shear stress and promote structural injury (28, 29). CHG places greater emphasis on glucose- and lipid-related components. Previous studies have suggested that glucose–lipid metabolic disturbances may promote oxidative stress, mitochondrial dysfunction, apoptosis, and lipid accumulation, which are involved in renal tubular epithelial cell (RTEC) injury and renal functional decline (30). Given that tubular injury is increasingly recognized as an important contributor to CKD progression (31), these metabolic abnormalities may represent one potential pathway linking IR-related disturbances with subsequent renal deterioration. Consistent with this concept, the observed association between CHG and RKFD may reflect the potential relevance of glucose–lipid metabolic stress to tubular injury and functional decline (30, 32); however, these mechanisms were not directly measured in the present cohort and require further validation. With subsequent CKD progression, progressive nephron loss may lead to irreversible structural changes, including glomerulosclerosis, tubular atrophy, and interstitial fibrosis (33). These changes involve RTEC loss and maladaptive repair, with tubular hypertrophy and profibrotic factor release contributing to fibrosis progression (31). TyG-BMI incorporates overall adiposity and IR and may better reflect the cumulative effects of long-term metabolic burden and hemodynamic stress on renal remodeling. Prior research has shown that, compared with age-matched populations, patients with incident kidney failure experienced an approximately twofold greater increase in BMI, highlighting the potential role of systemic obesity in renal deterioration (34). Thus, the stronger association of TyG-BMI with adverse renal outcomes may reflect the cumulative metabolic burden associated with CKD progression. Ultimately, extensive nephron loss and reduced functional reserve impair renal filtration and metabolic capacity, leading to uremia (35).

Although the mechanisms discussed above provide biologically plausible explanations for how metabolic dysfunction may contribute to CKD onset and progression, the relationship between metabolic dysfunction and kidney disease may be bidirectional (5). CKD may itself promote systemic IR through chronic inflammation, oxidative stress, neurohormonal activation, and impaired insulin signaling (5, 36). In parallel, declining kidney function is associated with disturbances in lipid metabolism, including impaired clearance of TG-rich lipoproteins, accumulation of atherogenic remnant particles, and abnormalities in HDL metabolism (37, 38). These metabolic alterations may, in turn, affect several of the surrogate IR indices evaluated in this study. Therefore, the observed associations may reflect a complex bidirectional interplay whereby metabolic dysfunction contributes to renal injury, whereas renal dysfunction further exacerbates IR and dyslipidemia. Given the retrospective observational design, the possibility of such bidirectional relationships cannot be fully excluded.

To support the robustness of our findings, we implemented multiple design and analytical strategies to assess and minimize potential sources of bias. First, to reduce concerns regarding reverse causation, surrogate IR indices were assessed at baseline before incident CKD developed during follow-up, and participants with baseline renal impairment were excluded. Second, to assess the robustness of CKD ascertainment, we applied a more stringent CKD definition and excluded participants with long intervals between consecutive eGFR measurements to reduce the influence of physiological eGFR variability. We also examined potential bias related to age-related GFR decline and obesity-related differences in creatinine generation. CKD events occurred across a broad age range, with only 18.2% occurring among participants aged ≥60 years. Age was incorporated into the eGFR calculation and further adjusted for in multivariable models. Moreover, excluding participants with BMI ≥ 28 kg/m^2^ did not materially change the findings. Collectively, these analyses suggest that the observed associations were unlikely to be primarily explained by reverse causation, uncertainty in CKD ascertainment, age-related kidney function decline, or obesity-related differences in creatinine generation.

Our findings may have implications for risk-based assessment across the CKD continuum. Consistent with KDIGO 2024, which emphasizes risk assessment beyond eGFR alone (39), surrogate IR indices may facilitate earlier identification of high-risk individuals. The relatively strong association between TyG-WC and incident CKD suggests that visceral adiposity-related IR represents an early, modifiable risk state, where intervention should prioritize metabolic risk modification, including weight control, physical activity, dietary optimization, smoking avoidance, and blood pressure management. The relatively stronger association between CHG and RKFD suggests that glucose–lipid metabolic disturbances may be particularly relevant to subsequent renal deterioration. These findings may support closer monitoring of renal function and metabolic risk factors after CKD onset. The association between TyG-BMI and adverse renal outcomes may reflect the cumulative burden of long-term metabolic stress and could help identify individuals warranting closer risk assessment. These individuals may warrant closer risk assessment and follow-up, with attention to blood pressure control, metabolic risk factors, avoidance of potential nephrotoxic exposures, and timely multidisciplinary evaluation when appropriate. Collectively, these findings suggest that different surrogate IR indices may provide complementary information for risk assessment across CKD development and progression.

The study has several limitations. First, its single-center design and health examination-based population may have introduced selection bias and limited generalizability. Second, although combining cystatin C with Scr can improve eGFR accuracy, its cost is approximately 10-fold higher than that of Scr testing, limiting widespread clinical use. Although standardized pre-examination procedures and sensitivity analyses using a stricter CKD definition and excluding participants with obesity yielded generally consistent results, residual bias related to muscle mass, body size, dietary protein intake, and other non-GFR determinants of serum creatinine cannot be fully excluded. Third, the relatively small number of CKD progression events constrained covariate adjustment and reduced statistical power. Fourth, all-cause mortality data were unavailable, precluding competing risk analyses such as Fine–Gray models. Fifth, CKD progression was assessed using RKFD and surrogate clinical endpoints rather than CKD stages 1–5, limiting evaluation across finer disease stages. Finally, uACR was unavailable for over 99% of participants, consistent with its underuse in real-world practice (40), precluding its inclusion in the primary outcome definition. Future multicenter prospective studies should include broader populations, cystatin C where feasible, sufficient event numbers, mortality data, more granular CKD staging, and systematic uACR measurement.

### 4.1 Conclusion

In this large cohort study, surrogate IR indices were associated with an increased risk of incident CKD and were generally higher in groups with poorer renal outcomes, suggesting a complex interplay between metabolic dysfunction and kidney disease. Among the evaluated indices, TyG-WC showed a relatively strong association with incident CKD, with an inflection point at 811.58. CHG and TyG-BMI showed relatively stronger associations with subsequent RKFD and adverse renal outcomes, respectively. TyG-WC may aid early risk identification and stratification, while exploratory mediation analysis suggested a small indirect effect through absolute monocyte count.

## Data Availability

The raw data supporting the conclusions of this article will be made available by the authors, without undue reservation.
