# Supplementary material for: Stage-specific associations of nutrition-related surrogate insulin resistance indices across the chronic kidney disease continuum: evidence from a Chinese cohort
Source: Front Nutr. 2026 Oct 19;13:1943087. doi: 10.3389/fnut.2026.1943087 (PMC13724407; doi:10.3389/fnut.2026.1943087)
Supplement: Supplementary file 1 [file Data_Sheet_1.docx]

**Supplementary Materials**

**Supplementary Tables**

Supplementary Table 1. The percentage of missing data in the present study

Supplementary Table 2. Schoenfeld residual tests of the proportional hazards assumption in the fully adjusted Cox model

Supplementary Table 3. Baseline characteristics of participants with CKD by RKFD status

Supplementary Table 4. Baseline characteristics of participants with CKD by surrogate endpoint status

Supplementary Table 5. Threshold analysis of surrogate indices of insulin resistance for incident CKD

Supplementary Table 6. Associations of surrogate indices of insulin resistance with progression to RKFD in participants with CKD

Supplementary Table 7. Associations of surrogate indices of insulin resistance with progression to surrogate endpoints in participants with CKD

Supplementary Table 8. Associations of surrogate indices of insulin resistance with incident CKD after excluding participants with >2-year intervals between eGFR measurements

Supplementary Table 9. Associations of surrogate indices of insulin resistance with incident CKD after excluding participants receiving medications

Supplementary Table 10. Associations of surrogate indices of insulin resistance with incident CKD using a more stringent definition

Supplementary Table 11. Associations of surrogate indices of insulin resistance with incident CKD after further adjustment for for additional covariates

Supplementary Table 12. Associations of surrogate indices of insulin resistance with incident CKD after excluding participants with BMI ≥28 kg/m²

Supplementary Table 13. Associations of surrogate indices of insulin resistance with progression to RKFD in participants with CKD after excluding those with BMI ≥28 kg/m²

Supplementary Table 14. Associations of surrogate indices of insulin resistance with progression to surrogate endpoints in participants with incident CKD after excluding those with BMI ≥28 kg/m²

Supplementary Table 15. Discriminative performance and prediction error of surrogate indices of insulin resistance for incident CKD

**Supplementary Figures**

Supplementary Figure 1. Directed acyclic graph illustrating the assumed relationships between surrogate indices of insulin resistance and chronic kidney disease

Supplementary Figure 2. Pairwise Spearman correlations among the 12 surrogate insulin resistance indices.

Supplementary Figure 3. Restricted cubic spline analyses of associations between surrogate indices of insulin resistance and RKFD in participants with CKD

Supplementary Figure 4. Restricted cubic spline analyses of associations between surrogate indices of insulin resistance and surrogate endpoints in participants with CKD

Supplementary Figure 5. Subgroup analyses of the association between TyG-WC and incident CKD

Supplementary Figure 6. Calibration plots of surrogate indices of insulin resistance for incident CKD at 3 years

Supplementary Figure 7. Decision curve analysis of surrogate indices of insulin resistance for incident CKD at 3 years

Supplementary Figure 8. Time-dependent Brier scores of surrogate indices of insulin resistance for incident CKD

Supplementary Figure 9. Kaplan–Meier curves for risk stratification of incident CKD based on TyG-WC–derived median predicted 3-year risk

Supplementary Figure 10. Mediation analysis of monocytes in the associations between surrogate indices of insulin resistance and incident CKD

**Supplementary Methods**

The following formulas were used to calculate surrogate indices of insulin resistance:

TyG = ln [TG (mg/dL) × FPG (mg/dL) / 2] [1]

TG/HDL-C ratio = TG (mg/dL) / HDL-C (mg/dL) [2]

METS-IR = ln [2 × FPG (mg/dL) + TG (mg/dL)] × BMI (kg/m²) / ln [HDL-C (mg/dL)] [3]

TyG-BMI = TyG × BMI (kg/m²) [1]

TyG-WC = TyG × WC (cm) [1]

TyG-WHtR = TyG × (WC (cm) / Height (cm)) [4]

METS-VF = 4.466 + 0.011 × [ln (METS-IR)]³ + 3.239 × [ln (WHtR)]³ + 0.319 × Sex + 0.594 × ln [Age (years)], where Sex was coded as male = 1 and female = 0 [5]

LAP (male) = [WC (cm) − 65] × TG (mmol/L) [1]

LAP (female) = [WC (cm) − 58] × TG (mmol/L) [1]

CVAI (male) = −267.93 + 0.68 × Age (years) + 0.03 × BMI (kg/m²) + 4.00 × WC (cm) + 22.00 × log10 [TG (mmol/L)] − 16.32 × HDL-C (mmol/L) [1]

CVAI (female) = −187.32 + 1.71 × Age (years) + 4.23 × BMI (kg/m²) + 1.12 × WC (cm) + 39.76 × log10 [TG (mmol/L)] − 11.66 × HDL-C (mmol/L) [1]

AIP = log10 [TG (mmol/L) / HDL-C (mmol/L)] [1]

CHG = ln [TC (mg/dL) × FPG (mg/dL) / (2 × HDL-C (mg/dL))] [6]

RC = TC (mg/dL) − HDL-C (mg/dL) − LDL-C (mg/dL) [7]

Unit conversions were as follows: cholesterol (TC, HDL-C, LDL-C), 1 mmol/L = 38.67 mg/dL; triglycerides (TG), 1 mmol/L = 88.57 mg/dL; FPG, 1 mmol/L = 18 mg/dL.

**References**

[1]	Liu S, Sun H, Liu J, Wang G. Accessing the relationship between six surrogate insulin resistance indexes and the incidence of rapid kidney function decline and the progression to chronic kidney disease among middle-aged and older adults in China: Results from the China health and retirement longitudinal study. Diabetes Research and Clinical Practice 2024;212:111705. https://doi.org/10.1016/j.diabres.2024.111705.

[2]	Wang X, Lin R, Ji X, Xu Z, Chang H, Gu X, et al. Insulin resistance mediates the association between adiposity markers and incident chronic kidney disease: Findings from the UK Biobank prospective cohort study. Diabetes Research and Clinical Practice 2025;225:112255. https://doi.org/10.1016/j.diabres.2025.112255.

[3]	Bello-Chavolla OY, Almeda-Valdes P, Gomez-Velasco D, Viveros-Ruiz T, Cruz-Bautista I, Romo-Romo A, et al. METS-IR, a novel score to evaluate insulin sensitivity, is predictive of visceral adiposity and incident type 2 diabetes. Eur J Endocrinol 2018;178:533–44. https://doi.org/10.1530/EJE-17-0883.

[4]	Dou J, Guo F, Guo C, Wu R-Y, Hu Y-W, Sun X-L, et al. Triglyceride glucose-waist height ratio index is a promising and robust glucose and lipid metabolism indicator for predicting long-term type 2 diabetes: insights from a Japanese prospective cohort data from 12 years. Diabetes Research and Clinical Practice 2025;227:112398. https://doi.org/10.1016/j.diabres.2025.112398.

[5]	Bello-Chavolla OY, Antonio-Villa NE, Vargas-Vázquez A, Viveros-Ruiz TL, Almeda-Valdes P, Gomez-Velasco D, et al. Metabolic Score for Visceral Fat (METS-VF), a novel estimator of intra-abdominal fat content and cardio-metabolic health. Clinical Nutrition 2020;39:1613–21. https://doi.org/10.1016/j.clnu.2019.07.012.

[6]	Liu H, Tang G, Yuan Y, Gu P, Wang A, Zhao S, et al. Cholesterol, high-density lipoprotein, and glucose index is associated with renal survival in patients with IgA nephropathy. Front Endocrinol 2025;16. https://doi.org/10.3389/fendo.2025.1643360.

[7]	Li Y, Zeng Q, Peng D, Hu P, Luo J, Zheng K, et al. Association of remnant cholesterol with insulin resistance and type 2 diabetes: mediation analyses from NHANES 1999–2020. Lipids Health Dis 2024;23:404. https://doi.org/10.1186/s12944-024-02393-6.

Supplementary Table 1. The percentage of missing data in the present study

| Variable | Total | Missing | MissPct (%) |
| --- | --- | --- | --- |
| WC | 48304 | 3736 | 7.73 |
| Weight | 48304 | 3736 | 7.73 |
| Height | 48304 | 3724 | 7.71 |
| Urea | 48304 | 3171 | 6.56 |
| DBP | 48304 | 2244 | 4.65 |
| SBP | 48304 | 2243 | 4.64 |
| HDL-C | 48304 | 1237 | 2.56 |
| LDL-C | 48304 | 1177 | 2.44 |
| TC | 48304 | 1087 | 2.25 |
| TG | 48304 | 1089 | 2.25 |
| FPG | 48304 | 826 | 1.71 |
| ALT | 48304 | 539 | 1.12 |
| RBC | 48304 | 494 | 1.02 |
| Hemoglobin | 48304 | 494 | 1.02 |
| WBC | 48304 | 494 | 1.02 |
| Lymphocyte | 48304 | 494 | 1.02 |
| Monocyte | 48304 | 494 | 1.02 |
| Neutrophil | 48304 | 494 | 1.02 |
| Platelets | 48304 | 494 | 1.02 |
| SUA | 48304 | 168 | 0.35 |
| Age | 48304 | 0 | 0 |
| Sex | 48304 | 0 | 0 |
| Smoking status | 48304 | 0 | 0 |
| Alcohol consumption | 48304 | 0 | 0 |
| Medication use | 48304 | 0 | 0 |
| Diabetes | 48304 | 0 | 0 |
| Dyslipidemia | 48304 | 0 | 0 |
| Hypertension | 48304 | 0 | 0 |
| Scr | 48304 | 0 | 0 |
| eGFR | 48304 | 0 | 0 |

Abbreviations: ALT, alanine aminotransferase; DBP, diastolic blood pressure; eGFR, estimated glomerular filtration rate; FPG, fasting plasma glucose; HDL-C, high-density lipoprotein cholesterol; LDL-C, low-density lipoprotein cholesterol; RBC, red blood cell count; Scr, serum creatinine; SBP, systolic blood pressure; SUA, serum uric acid; TC, total cholesterol; TG, triglycerides; WBC, white blood cell count; WC, waist circumference

Supplementary Table 2. Schoenfeld residual tests of the proportional hazards assumption in the fully adjusted Cox model

| Exposure | Analysis | Model | Term | Chisq | DF | *P* |
| --- | --- | --- | --- | --- | --- | --- |
| TyG | Continuous | Model3 | GLOBAL | 15.771 | 10 | 0.106 |
| TyG | Quartile | Model3 | GLOBAL | 20.159 | 12 | 0.064 |
| TG/HDL-C | Continuous | Model3 | GLOBAL | 13.732 | 10 | 0.186 |
| TG/HDL-C | Quartile | Model3 | GLOBAL | 17.541 | 12 | 0.130 |
| METS-IR | Continuous | Model3 | GLOBAL | 13.737 | 10 | 0.185 |
| METS-IR | Quartile | Model3 | GLOBAL | 18.044 | 12 | 0.114 |
| TyG-BMI | Continuous | Model3 | GLOBAL | 13.797 | 10 | 0.182 |
| TyG-BMI | Quartile | Model3 | GLOBAL | 19.326 | 12 | 0.081 |
| TyG-WC | Continuous | Model3 | GLOBAL | 13.708 | 10 | 0.187 |
| TyG-WC | Quartile | Model3 | GLOBAL | 17.408 | 12 | 0.135 |
| TyG-WHtR | Continuous | Model3 | GLOBAL | 13.825 | 10 | 0.181 |
| TyG-WHtR | Quartile | Model3 | GLOBAL | 18.356 | 12 | 0.105 |
| METS-VF | Continuous | Model3 | GLOBAL | 13.818 | 10 | 0.181 |
| METS-VF | Quartile | Model3 | GLOBAL | 15.631 | 12 | 0.209 |
| LAP | Continuous | Model3 | GLOBAL | 14.188 | 10 | 0.165 |
| LAP | Quartile | Model3 | GLOBAL | 20.031 | 12 | 0.067 |
| CVAI | Continuous | Model3 | GLOBAL | 13.863 | 10 | 0.179 |
| CVAI | Quartile | Model3 | GLOBAL | 20.564 | 12 | 0.069 |
| AIP | Continuous | Model3 | GLOBAL | 14.978 | 10 | 0.133 |
| AIP | Quartile | Model3 | GLOBAL | 17.541 | 12 | 0.130 |
| CHG | Continuous | Model3 | GLOBAL | 19.190 | 10 | 0.068 |
| CHG | Quartile | Model3 | GLOBAL | 18.744 | 12 | 0.095 |
| RC | Continuous | Model3 | GLOBAL | 14.590 | 10 | 0.148 |
| RC | Quartile | Model3 | GLOBAL | 18.906 | 12 | 0.091 |

*Note:* Model 3 was adjusted for age, sex, smoking status, alcohol consumption, medication use, diabetes, dyslipidemia, hypertension, and eGFR. GLOBAL denotes the global test of the proportional hazards assumption based on Schoenfeld residuals.

Abbreviations: AIP, atherogenic index of plasma; CHG, cholesterol–high-density lipoprotein–glucose index; Chisq, chi‑square statistic; CVAI, Chinese visceral adiposity index; DF, degrees of freedom; LAP, lipid accumulation product; METS‑IR, metabolic score for insulin resistance; METS‑VF, metabolic score for visceral fat; RC, remnant cholesterol; TG/HDL‑C, triglyceride‑to‑high‑density lipoprotein cholesterol ratio; TyG, triglyceride–glucose index; TyG-BMI, triglyceride–glucose index combined with body mass index; TyG-WC, triglyceride–glucose index combined with waist circumference; TyG-WHtR, triglyceride–glucose index combined with waist-to-height ratio

Supplementary Table 3. Baseline characteristics of participants with CKD by RKFD status

| Variables | CKD with no RKFD progression (n = 1038) | CKD with RKFD progression (n = 54) | Overall (n = 1092) | *P* |
| --- | --- | --- | --- | --- |
| Age (years) | 47.00 (36.00, 56.00) | 51.00 (46.00, 59.00) | 47.00 (36.00, 56.00) | 0.013 |
| Sex (%) |  |  |  | 0.398 |
| Men | 794.00 (76.49%) | 44.00 (81.48%) | 838.00 (76.74%) |  |
| Women | 244.00 (23.51%) | 10.00 (18.52%) | 254.00 (23.26%) |  |
| Smoking status (%) | 157.00 (15.13%) | 21.00 (38.89%) | 178.00 (16.30%) | <0.001 |
| Alcohol consumption (%) | 142.00 (13.68%) | 23.00 (42.59%) | 165.00 (15.11%) | <0.001 |
| Medication use (%) | 260.00 (25.05%) | 21.00 (38.89%) | 281.00 (25.73%) | 0.023 |
| Diabetes (%) | 178.00 (17.15%) | 23.00 (42.59%) | 201.00 (18.41%) | <0.001 |
| Dyslipidemia (%) | 471.00 (45.38%) | 29.00 (53.70%) | 500.00 (45.79%) | 0.231 |
| Hypertension (%) | 449.00 (43.26%) | 31.00 (57.41%) | 480.00 (43.96%) | 0.041 |
| FPG (mmol/L) | 5.76 (5.31, 6.65) | 6.96 (5.34, 10.50) | 5.80 (5.31, 6.76) | <0.001 |
| SUA (μmol/L) | 372.25 (314.81, 438.60) | 396.22 (316.84, 497.68) | 372.35 (314.88, 440.39) | 0.163 |
| Urea (mmol/L) | 5.14 (4.37, 6.04) | 5.42 (4.86, 6.40) | 5.16 (4.40, 6.09) | 0.075 |
| Scr (μmol/L) | 71.91 (61.63, 81.96) | 75.69 (68.93, 87.87) | 72.05 (62.00, 82.05) | 0.029 |
| eGFR (mL/min/1.73 m²) | 107.45 (97.39, 115.90) | 101.00 (89.36, 109.90) | 107.22 (96.76, 115.66) | 0.005 |
| TyG | 9.00 (8.62, 9.47) | 9.35 (8.76, 9.80) | 9.01 (8.63, 9.49) | 0.007 |
| TG/HDL-C | 3.36 (2.15, 5.18) | 3.97 (2.47, 6.22) | 3.38 (2.17, 5.24) | 0.092 |
| METS-IR | 41.23 (35.71, 47.26) | 44.32 (37.12, 49.40) | 41.43 (35.81, 47.52) | 0.044 |
| TyG-BMI | 238.23 (208.71, 267.66) | 249.39 (227.92, 280.79) | 238.63 (208.97, 268.39) | 0.060 |
| TyG-WC | 828.31 (741.01, 911.70) | 885.09 (790.04, 963.99) | 829.87 (742.38, 916.27) | 0.013 |
| TyG-WHtR | 4.83 (4.35, 5.30) | 5.06 (4.64, 5.73) | 4.84 (4.35, 5.34) | 0.026 |
| METS-VF | 6.77 (6.30, 7.12) | 6.93 (6.59, 7.19) | 6.77 (6.31, 7.12) | 0.050 |
| LAP | 47.22 (28.08, 76.03) | 53.63 (32.34, 86.95) | 47.59 (28.08, 76.22) | 0.312 |
| CVAI | 119.72 (84.22, 149.20) | 138.81 (92.41, 159.97) | 120.03 (84.32, 150.15) | 0.046 |
| AIP | 0.17 (-0.03, 0.35) | 0.24 (0.03, 0.43) | 0.17 (-0.02, 0.36) | 0.092 |
| CHG | 5.44 (5.20, 5.67) | 5.57 (5.30, 6.07) | 5.44 (5.20, 5.69) | 0.016 |
| RC | 37.90 (29.00, 51.43) | 38.48 (29.39, 49.11) | 37.90 (29.20, 51.24) | 0.784 |

Abbreviations: AIP, atherogenic index of plasma; CHG, cholesterol–high-density lipoprotein–glucose index; CKD, chronic kidney disease; CVAI, Chinese visceral adiposity index; FPG, fasting plasma glucose; LAP, lipid accumulation product; METS‑IR, metabolic score for insulin resistance; METS‑VF, metabolic score for visceral fat; RC, remnant cholesterol; RKFD, rapid kidney function decline; Scr, serum creatinine; SUA, serum uric acid; TG/HDL‑C, triglyceride‑to‑high‑density lipoprotein cholesterol ratio; TyG, triglyceride–glucose index; TyG-BMI, triglyceride–glucose index combined with body mass index; TyG-WC, triglyceride–glucose index combined with waist circumference; TyG-WHtR, triglyceride–glucose index combined with waist-to-height ratio; eGFR, estimated glomerular filtration rate

Supplementary Table 4. Baseline characteristics of participants with CKD by surrogate endpoint status

| Variables | CKD with no endpoint progression (n = 1075) | CKD with endpoint progression (n = 17) | Overall (n = 1092) | *P* |
| --- | --- | --- | --- | --- |
| Age (years) | 47.00 (36.00, 56.00) | 54.00 (51.00, 65.00) | 47.00 (36.00, 56.00) | 0.005 |
| Sex (%) |  |  |  | 0.143 |
| Men | 822.00 (76.47%) | 16.00 (94.12%) | 838.00 (76.74%) |  |
| Women | 253.00 (23.53%) | 1.00 (5.88%) | 254.00 (23.26%) |  |
| Smoking status (%) | 163.00 (15.16%) | 15.00 (88.24%) | 178.00 (16.30%) | <0.001 |
| Alcohol consumption (%) | 151.00 (14.05%) | 14.00 (82.35%) | 165.00 (15.11%) | <0.001 |
| Medication use (%) | 273.00 (25.40%) | 8.00 (47.06%) | 281.00 (25.73%) | 0.052 |
| Diabetes (%) | 190.00 (17.67%) | 11.00 (64.71%) | 201.00 (18.41%) | <0.001 |
| Dyslipidemia (%) | 489.00 (45.49%) | 11.00 (64.71%) | 500.00 (45.79%) | 0.115 |
| Hypertension (%) | 468.00 (43.53%) | 12.00 (70.59%) | 480.00 (43.96%) | 0.026 |
| FPG (mmol/L) | 5.77 (5.31, 6.69) | 11.07 (7.69, 16.92) | 5.80 (5.31, 6.76) | <0.001 |
| SUA (μmol/L) | 372.28 (314.71, 439.13) | 497.68 (339.57, 565.79) | 372.35 (314.88, 440.39) | 0.016 |
| Urea (mmol/L) | 5.15 (4.38, 6.04) | 6.19 (5.10, 7.30) | 5.16 (4.40, 6.09) | <0.001 |
| Scr (μmol/L) | 72.00 (61.96, 81.96) | 82.00 (74.64, 92.59) | 72.05 (62.00, 82.05) | 0.003 |
| eGFR (mL/min/1.73 m²) | 107.42 (97.22, 115.81) | 87.71 (82.01, 102.28) | 107.22 (96.76, 115.66) | <0.001 |
| TyG | 9.01 (8.62, 9.47) | 9.89 (9.31, 10.41) | 9.01 (8.63, 9.49) | <0.001 |
| TG/HDL-C | 3.37 (2.16, 5.18) | 6.43 (3.10, 7.67) | 3.38 (2.17, 5.24) | 0.012 |
| METS-IR | 41.25 (35.71, 47.27) | 48.62 (44.21, 52.37) | 41.43 (35.81, 47.52) | <0.001 |
| TyG-BMI | 238.35 (208.54, 267.34) | 270.04 (247.46, 283.30) | 238.63 (208.97, 268.39) | 0.002 |
| TyG-WC | 829.14 (740.85, 913.45) | 937.42 (856.05, 1008.29) | 829.87 (742.38, 916.27) | <0.001 |
| TyG-WHtR | 4.83 (4.34, 5.30) | 5.45 (5.07, 5.83) | 4.84 (4.35, 5.34) | <0.001 |
| METS-VF | 6.77 (6.30, 7.12) | 7.18 (6.74, 7.41) | 6.77 (6.31, 7.12) | 0.003 |
| LAP | 47.26 (27.93, 76.03) | 75.45 (40.61, 93.80) | 47.59 (28.08, 76.22) | 0.048 |
| CVAI | 119.84 (84.05, 149.74) | 144.77 (118.27, 159.97) | 120.03 (84.32, 150.15) | 0.015 |
| AIP | 0.17 (-0.03, 0.35) | 0.45 (0.13, 0.53) | 0.17 (-0.02, 0.36) | 0.012 |
| CHG | 5.44 (5.20, 5.68) | 6.12 (5.56, 6.97) | 5.44 (5.20, 5.69) | <0.001 |
| RC | 37.51 (29.00, 50.66) | 49.88 (42.92, 61.49) | 37.90 (29.20, 51.24) | 0.009 |

Abbreviations: AIP, atherogenic index of plasma; CHG, cholesterol–high-density lipoprotein–glucose index; CKD, chronic kidney disease; CVAI, Chinese visceral adiposity index; eGFR, estimated glomerular filtration rate; FPG, fasting plasma glucose; LAP, lipid accumulation product; METS‑IR, metabolic score for insulin resistance; METS‑VF, metabolic score for visceral fat; RC, remnant cholesterol; Scr, serum creatinine; SUA, serum uric acid; TG/HDL‑C, triglyceride‑to‑high‑density lipoprotein cholesterol ratio; TyG, triglyceride–glucose index; TyG-BMI, triglyceride–glucose index combined with body mass index; TyG-WC, triglyceride–glucose index combined with waist circumference; TyG-WHtR, triglyceride–glucose index combined with waist-to-height ratio

Supplementary Table 5. Threshold analysis of surrogate indices of insulin resistance for incident CKD

| Exposure | Inflection point | Below threshold HR per SD (95% CI) | Below *P* | Above threshold HR per SD (95% CI) | Above *P* | *P* for LRT |
| --- | --- | --- | --- | --- | --- | --- |
| TG/HDL-C | 3.29 | 103.29 (23.24, 459.06) | <0.001 | 1.00 (0.94, 1.06) | 0.971 | <0.001 |
| METS-IR | 43.52 | 3.33 (2.53, 4.38) | <0.001 | 1.02 (0.99, 1.05) | 0.160 | <0.001 |
| TyG-BMI | 252.68 | 2.44 (1.98, 2.99) | <0.001 | 1.02 (0.99, 1.04) | 0.152 | <0.001 |
| TyG-WC | 811.58 | 1.71 (1.47, 1.98) | <0.001 | 1.1 (1.07, 1.13) | <0.001 | <0.001 |
| TyG-WHtR | 4.98 | 1.66 (1.46, 1.87) | <0.001 | 1.08 (1.04, 1.11) | <0.001 | <0.001 |
| METS-VF | 7.05 | 1.53 (1.35, 1.72) | <0.001 | 1.19 (1.08, 1.31) | <0.001 | 0.001 |
| LAP | 42.21 | 2.58 (1.93, 3.43) | <0.001 | 1.06 (1.02, 1.1) | 0.002 | <0.001 |
| CVAI | 117.98 | 1.73 (1.47, 2.05) | <0.001 | 1.03 (1.01, 1.05) | 0.005 | <0.001 |
| AIP | 0.16 | 1.41 (1.24, 1.62) | <0.001 | 1.21 (1.09, 1.33) | <0.001 | 0.080 |
| RC | 51.04 | 1.38 (1.23, 1.55) | <0.001 | 1.08 (1.02, 1.14) | 0.007 | <0.001 |

*Notes:* Restricted cubic spline models were adjusted for age, sex, smoking status, alcohol consumption, medication use, diabetes, dyslipidemia, hypertension, and eGFR. All surrogate indices of insulin resistance were standardized prior to modeling. Threshold analyses were performed using the fully adjusted models.

Abbreviations: AIP, atherogenic index of plasma; CI, confidence interval; CKD, chronic kidney disease; CVAI, Chinese visceral adiposity index; eGFR, estimated glomerular filtration rate; HR, hazard ratio; LAP, lipid accumulation product; LRT, likelihood ratio test; METS-IR, metabolic score for insulin resistance; METS-VF, metabolic score for visceral fat; RC, remnant cholesterol; SD, standard deviation; TG/HDL-C, triglyceride to high-density lipoprotein cholesterol ratio; TyG-BMI, triglyceride–glucose index combined with body mass index; TyG-WC, triglyceride–glucose index combined with waist circumference; TyG-WHtR, triglyceride–glucose index combined with waist-to-height ratio

Supplementary Table 6. Associations of surrogate indices of insulin resistance with progression to RKFD in participants with CKD

| Exposure | Levels | Model 1 | | Model 2 | | Model 3 | |
| --- | --- | --- | --- | --- | --- | --- | --- |
|  |  | OR (95% CI) | *P* | OR (95% CI) | *P* | OR (95% CI) | *P* |
| TyG | Continuous | 1.83 (1.25-2.69) | 0.002 | 1.72 (1.16-2.57) | 0.008 | 1.77 (1.18-2.64) | 0.006 |
|  | Per 1-SD | 1.49 (1.16-1.92) | 0.002 | 1.43 (1.10-1.87) | 0.008 | 1.46 (1.12-1.90) | 0.006 |
| TG/HDL-C | Continuous | 1.01 (0.96-1.06) | 0.710 | 1.01 (0.96-1.06) | 0.767 | 1.01 (0.96-1.06) | 0.700 |
|  | Per 1-SD | 1.05 (0.83-1.33) | 0.710 | 1.04 (0.81-1.33) | 0.767 | 1.05 (0.82-1.34) | 0.700 |
| METS-IR | Continuous | 1.02 (1.00-1.05) | 0.077 | 1.02 (1.00-1.06) | 0.096 | 1.03 (1.00-1.06) | 0.081 |
|  | Per 1-SD | 1.26 (0.98-1.62) | 0.077 | 1.26 (0.96-1.65) | 0.096 | 1.28 (0.97-1.68) | 0.081 |
| TyG-BMI | Continuous | 1.00 (1.00-1.01) | 0.138 | 1.00 (1.00-1.01) | 0.200 | 1.00 (1.00-1.01) | 0.192 |
|  | Per 1-SD | 1.22 (0.94-1.59) | 0.138 | 1.20 (0.91-1.60) | 0.200 | 1.21 (0.91-1.61) | 0.192 |
| TyG-WC | Continuous | 1.00 (1.00-1.00) | 0.035 | 1.00 (1.00-1.00) | 0.092 | 1.00 (1.00-1.00) | 0.078 |
|  | Per 1-SD | 1.34 (1.02-1.76) | 0.035 | 1.30 (0.96-1.75) | 0.092 | 1.32 (0.97-1.79) | 0.078 |
| TyG-WHtR | Continuous | 1.50 (1.05-2.15) | 0.026 | 1.38 (0.94-2.02) | 0.104 | 1.40 (0.95-2.07) | 0.089 |
|  | Per 1-SD | 1.36 (1.04-1.77) | 0.026 | 1.27 (0.95-1.69) | 0.104 | 1.29 (0.96-1.72) | 0.089 |
| METS-VF | Continuous | 1.35 (0.88-2.09) | 0.174 | 1.03 (0.60-1.76) | 0.920 | 1.03 (0.60-1.77) | 0.909 |
|  | Per 1-SD | 1.24 (0.91-1.69) | 0.174 | 1.02 (0.69-1.50) | 0.920 | 1.02 (0.69-1.51) | 0.909 |
| LAP | Continuous | 1.00 (1.00-1.01) | 0.612 | 1.00 (1.00-1.01) | 0.753 | 1.00 (1.00-1.01) | 0.728 |
|  | Per 1-SD | 1.07 (0.83-1.38) | 0.612 | 1.04 (0.80-1.36) | 0.753 | 1.05 (0.80-1.37) | 0.728 |
| CVAI | Continuous | 1.00 (1.00-1.01) | 0.106 | 1.00 (1.00-1.01) | 0.493 | 1.00 (1.00-1.01) | 0.484 |
|  | Per 1-SD | 1.26 (0.95-1.68) | 0.106 | 1.12 (0.80-1.57) | 0.493 | 1.13 (0.80-1.58) | 0.484 |
| AIP | Continuous | 1.86 (0.79-4.38) | 0.156 | 1.76 (0.71-4.36) | 0.219 | 1.82 (0.73-4.53) | 0.201 |
|  | Per 1-SD | 1.21 (0.93-1.58) | 0.156 | 1.19 (0.90-1.57) | 0.219 | 1.20 (0.91-1.59) | 0.201 |
| CHG | Continuous | 2.48 (1.57-3.93) | <0.001 | 2.33 (1.45-3.75) | <0.001 | 2.55 (1.56-4.15) | <0.001 |
|  | Per 1-SD | 1.54 (1.24-1.91) | <0.001 | 1.49 (1.19-1.87) | <0.001 | 1.56 (1.23-1.96) | <0.001 |
| RC | Continuous | 1.00 (0.99-1.01) | 0.957 | 1.00 (0.99-1.01) | 0.908 | 1.00 (0.99-1.01) | 0.991 |
|  | Per 1-SD | 0.99 (0.75-1.31) | 0.957 | 0.98 (0.74-1.31) | 0.908 | 1.00 (0.75-1.33) | 0.991 |

*Notes:* Model 1 was unadjusted; Model 2 was adjusted for age and sex; Model 3 was further adjusted for eGFR.

Abbreviations: AIP, atherogenic index of plasma; CHG, cholesterol–high-density lipoprotein–glucose index; CI, confidence interval; CKD, chronic kidney disease; CVAI, Chinese visceral adiposity index; eGFR, estimated glomerular filtration rate; LAP, lipid accumulation product; METS-IR, metabolic score for insulin resistance; METS-VF, metabolic score for visceral fat; OR, odds ratio; RC, remnant cholesterol; RKFD, rapid kidney function decline; SD, standard deviation; TG/HDL-C, triglyceride-to-high-density lipoprotein cholesterol ratio; TyG, triglyceride–glucose index; TyG-BMI, triglyceride–glucose index combined with body mass index; TyG-WC, triglyceride–glucose index combined with waist circumference; TyG-WHtR, triglyceride–glucose index combined with waist-to-height ratio

Supplementary Table 7. Associations of surrogate indices of insulin resistance with progression to surrogate endpoints in participants with CKD

| Exposure | Levels | Model 1 | | Model 2 | | Model 3 | |
| --- | --- | --- | --- | --- | --- | --- | --- |
|  |  | HR (95% CI) | *P* | HR (95% CI) | *P* | HR (95% CI) | *P* |
| TyG | Continuous | 2.66 (1.53-4.63) | <0.001 | 2.31 (1.32-4.02) | 0.003 | 2.21 (1.25-3.92) | 0.007 |
|  | Per 1-SD | 1.92 (1.33-2.77) | <0.001 | 1.74 (1.21-2.52) | 0.003 | 1.70 (1.16-2.48) | 0.007 |
| TG/HDL-C | Continuous | 1.03 (0.97-1.10) | 0.321 | 1.04 (0.97-1.11) | 0.240 | 1.04 (0.97-1.11) | 0.245 |
|  | Per 1-SD | 1.15 (0.87-1.53) | 0.321 | 1.20 (0.89-1.62) | 0.240 | 1.21 (0.88-1.66) | 0.245 |
| METS-IR | Continuous | 1.07 (1.02-1.12) | 0.003 | 1.09 (1.03-1.14) | 0.002 | 1.10 (1.04-1.16) | <0.001 |
|  | Per 1-SD | 1.87 (1.24-2.81) | 0.003 | 2.15 (1.34-3.46) | 0.002 | 2.36 (1.44-3.86) | <0.001 |
| TyG-BMI | Continuous | 1.02 (1.00-1.03) | 0.004 | 1.02 (1.01-1.03) | 0.003 | 1.02 (1.01-1.03) | 0.003 |
|  | Per 1-SD | 2.03 (1.26-3.28) | 0.004 | 2.44 (1.34-4.43) | 0.003 | 2.55 (1.38-4.71) | 0.003 |
| TyG-WC | Continuous | 1.01 (1.00-1.01) | 0.002 | 1.01 (1.00-1.01) | 0.008 | 1.01 (1.00-1.01) | 0.011 |
|  | Per 1-SD | 2.40 (1.38-4.17) | 0.002 | 2.29 (1.24-4.22) | 0.008 | 2.24 (1.20-4.18) | 0.011 |
| TyG-WHtR | Continuous | 3.29 (1.64-6.63) | <0.001 | 3.05 (1.42-6.57) | 0.004 | 3.08 (1.41-6.75) | 0.005 |
|  | Per 1-SD | 2.49 (1.46-4.26) | <0.001 | 2.35 (1.31-4.23) | 0.004 | 2.37 (1.30-4.32) | 0.005 |
| METS-VF | Continuous | 5.05 (1.37-18.57) | 0.015 | 2.64 (0.60-11.66) | 0.200 | 3.14 (0.66-14.91) | 0.150 |
|  | Per 1-SD | 3.21 (1.26-8.18) | 0.015 | 2.01 (0.69-5.85) | 0.200 | 2.28 (0.74-6.99) | 0.150 |
| LAP | Continuous | 1.01 (1.00-1.01) | 0.256 | 1.01 (1.00-1.02) | 0.302 | 1.00 (0.99-1.02) | 0.396 |
|  | Per 1-SD | 1.27 (0.84-1.90) | 0.256 | 1.27 (0.81-1.98) | 0.302 | 1.23 (0.76-1.97) | 0.396 |
| CVAI | Continuous | 1.02 (1.00-1.03) | 0.016 | 1.01 (1.00-1.03) | 0.169 | 1.01 (1.00-1.03) | 0.159 |
|  | Per 1-SD | 2.23 (1.16-4.28) | 0.016 | 1.70 (0.80-3.61) | 0.169 | 1.75 (0.80-3.80) | 0.159 |
| AIP | Continuous | 3.63 (1.00-13.19) | 0.050 | 3.99 (0.97-16.46) | 0.056 | 3.84 (0.88-16.78) | 0.074 |
|  | Per 1-SD | 1.50 (1.00-2.24) | 0.050 | 1.54 (0.99-2.40) | 0.056 | 1.52 (0.96-2.41) | 0.074 |
| CHG | Continuous | 4.24 (2.36-7.59) | <0.001 | 4.09 (2.22-7.54) | <0.001 | 4.01 (2.16-7.45) | <0.001 |
|  | Per 1-SD | 1.99 (1.51-2.63) | <0.001 | 1.96 (1.46-2.62) | <0.001 | 1.94 (1.44-2.60) | <0.001 |
| RC | Continuous | 1.01 (1.00-1.02) | 0.254 | 1.01 (1.00-1.02) | 0.147 | 1.01 (1.00-1.02) | 0.090 |
|  | Per 1-SD | 1.17 (0.89-1.54) | 0.254 | 1.23 (0.93-1.63) | 0.147 | 1.28 (0.96-1.70) | 0.090 |

*Notes:* Model 1 was unadjusted; Model 2 was adjusted for age and sex; Model 3 was further adjusted for eGFR. Surrogate endpoints were defined as either a 40% decline in eGFR or a doubling of serum creatinine.

Abbreviations: AIP, atherogenic index of plasma; CHG, cholesterol–high-density lipoprotein–glucose index; CI, confidence interval; CKD, chronic kidney disease; CVAI, Chinese visceral adiposity index; eGFR, estimated glomerular filtration rate; HR, hazard ratio; LAP, lipid accumulation product; METS-IR, metabolic score for insulin resistance; METS-VF, metabolic score for visceral fat; RC, remnant cholesterol; SD, standard deviation; TG/HDL-C, triglyceride to high-density lipoprotein cholesterol ratio; TyG, triglyceride–glucose index; TyG-BMI, triglyceride–glucose index combined with body mass index; TyG-WC, triglyceride–glucose index combined with waist circumference; TyG-WHtR, triglyceride–glucose index combined with waist-to-height ratio

Supplementary Table 8. Associations of surrogate indices of insulin resistance with incident CKD after excluding participants with >2-year intervals between eGFR measurements

| Exposure | Levels | Events | PY | Incidence Rate | Model 1 | | Model 2 | | Model 3 | |
| --- | --- | --- | --- | --- | --- | --- | --- | --- | --- | --- |
|  |  |  |  |  | HR (95% CI) | *P* | HR (95% CI) | *P* | HR (95% CI) | *P* |
| TyG | Continuous |  |  |  | 2.50 (2.28-2.73) | <0.001 | 2.01 (1.81-2.23) | <0.001 | 1.82 (1.58-2.09) | <0.001 |
|  | Per 1-SD |  |  |  | 1.72 (1.63-1.81) | <0.001 | 1.51 (1.42-1.61) | <0.001 | 1.42 (1.31-1.55) | <0.001 |
|  | Q1 | 68 | 61393.435 | 1.108 | Reference | — | Reference | — | Reference | — |
|  | Q2 | 125 | 57178.001 | 2.186 | 1.93 (1.44-2.60) | <0.001 | 1.51 (1.13-2.04) | 0.006 | 1.45 (1.08-1.95) | 0.014 |
|  | Q3 | 198 | 55399.917 | 3.574 | 3.13 (2.37-4.12) | <0.001 | 2.08 (1.58-2.76) | <0.001 | 1.91 (1.44-2.54) | <0.001 |
|  | Q4 | 377 | 55095.68 | 6.843 | 5.99 (4.62-7.75) | <0.001 | 3.36 (2.57-4.39) | <0.001 | 2.68 (1.98-3.62) | <0.001 |
|  | *P* for trend |  |  |  |  | <0.001 |  | <0.001 |  | <0.001 |
| TG/HDL-C | Continuous |  |  |  | 1.00 (1.00-1.00) | 0.057 | 1.00 (1.00-1.00) | 0.239 | 1.00 (1.00-1.00) | 0.572 |
|  | Per 1-SD |  |  |  | 1.02 (1.00-1.04) | 0.057 | 1.02 (0.99-1.05) | 0.239 | 1.01 (0.97-1.06) | 0.572 |
|  | Q1 | 78 | 60292.239 | 1.294 | Reference | — | Reference | — | Reference | — |
|  | Q2 | 132 | 57195.192 | 2.308 | 1.76 (1.33-2.32) | <0.001 | 1.39 (1.05-1.84) | 0.021 | 1.31 (0.99-1.74) | 0.057 |
|  | Q3 | 217 | 55904.228 | 3.882 | 2.94 (2.27-3.80) | <0.001 | 1.94 (1.49-2.53) | <0.001 | 1.76 (1.35-2.30) | <0.001 |
|  | Q4 | 341 | 55675.374 | 6.125 | 4.63 (3.62-5.93) | <0.001 | 2.69 (2.08-3.47) | <0.001 | 2.34 (1.74-3.15) | <0.001 |
|  | *P* for trend |  |  |  |  | <0.001 |  | <0.001 |  | <0.001 |
| METS-IR | Continuous |  |  |  | 1.00 (1.00-1.00) | <0.001 | 1.00 (1.00-1.00) | <0.001 | 1.00 (1.00-1.00) | <0.001 |
|  | Per 1-SD |  |  |  | 1.03 (1.02-1.04) | <0.001 | 1.04 (1.02-1.05) | <0.001 | 1.03 (1.01-1.05) | <0.001 |
|  | Q1 | 60 | 58518.805 | 1.025 | Reference | — | Reference | — | Reference | — |
|  | Q2 | 106 | 58176.155 | 1.822 | 1.78 (1.30-2.44) | <0.001 | 1.28 (0.93-1.77) | 0.128 | 1.27 (0.92-1.75) | 0.151 |
|  | Q3 | 203 | 57500.478 | 3.530 | 3.44 (2.58-4.59) | <0.001 | 1.98 (1.46-2.67) | <0.001 | 1.80 (1.32-2.45) | <0.001 |
|  | Q4 | 399 | 54871.595 | 7.272 | 7.03 (5.36-9.22) | <0.001 | 3.77 (2.82-5.05) | <0.001 | 3.00 (2.20-4.10) | <0.001 |
|  | *P* for trend |  |  |  |  | <0.001 |  | <0.001 |  | <0.001 |
| TyG-BMI | Continuous |  |  |  | 1.00 (1.00-1.00) | <0.001 | 1.00 (1.00-1.00) | <0.001 | 1.00 (1.00-1.00) | <0.001 |
|  | Per 1-SD |  |  |  | 1.02 (1.02-1.03) | <0.001 | 1.03 (1.02-1.04) | <0.001 | 1.03 (1.01-1.04) | <0.001 |
|  | Q1 | 59 | 59420.923 | 0.993 | Reference | — | Reference | — | Reference | — |
|  | Q2 | 115 | 58017.065 | 1.982 | 1.98 (1.45-2.72) | <0.001 | 1.39 (1.01-1.91) | 0.042 | 1.36 (0.99-1.87) | 0.061 |
|  | Q3 | 198 | 56617.304 | 3.497 | 3.49 (2.61-4.66) | <0.001 | 1.93 (1.43-2.62) | <0.001 | 1.74 (1.28-2.38) | <0.001 |
|  | Q4 | 396 | 55011.742 | 7.198 | 7.13 (5.42-9.37) | <0.001 | 3.69 (2.76-4.93) | <0.001 | 2.80 (2.05-3.81) | <0.001 |
|  | *P* for trend |  |  |  |  | <0.001 |  | <0.001 |  | <0.001 |
| TyG-WC | Continuous |  |  |  | 1.00 (1.00-1.00) | <0.001 | 1.00 (1.00-1.00) | <0.001 | 1.00 (1.00-1.00) | <0.001 |
|  | Per 1-SD |  |  |  | 1.16 (1.14-1.17) | <0.001 | 1.16 (1.14-1.18) | <0.001 | 1.13 (1.11-1.16) | <0.001 |
|  | Q1 | 51 | 59613.967 | 0.856 | Reference | — | Reference | — | Reference | — |
|  | Q2 | 103 | 58164.523 | 1.771 | 2.06 (1.47-2.88) | <0.001 | 1.48 (1.05-2.09) | 0.026 | 1.48 (1.05-2.09) | 0.027 |
|  | Q3 | 191 | 57205.801 | 3.339 | 3.88 (2.85-5.28) | <0.001 | 2.16 (1.54-3.02) | <0.001 | 2.00 (1.42-2.82) | <0.001 |
|  | Q4 | 423 | 54082.742 | 7.821 | 8.95 (6.69-11.97) | <0.001 | 4.44 (3.19-6.18) | <0.001 | 3.44 (2.42-4.88) | <0.001 |
|  | *P* for trend |  |  |  |  | <0.001 |  | <0.001 |  | <0.001 |
| TyG-WHtR | Continuous |  |  |  | 1.17 (1.15-1.19) | <0.001 | 1.18 (1.16-1.21) | <0.001 | 1.16 (1.13-1.19) | <0.001 |
|  | Per 1-SD |  |  |  | 1.12 (1.11-1.14) | <0.001 | 1.13 (1.11-1.15) | <0.001 | 1.11 (1.09-1.14) | <0.001 |
|  | Q1 | 59 | 59512.431 | 0.991 | Reference | — | Reference | — | Reference | — |
|  | Q2 | 99 | 58122.337 | 1.703 | 1.71 (1.24-2.35) | 0.001 | 1.22 (0.88-1.70) | 0.230 | 1.24 (0.89-1.72) | 0.204 |
|  | Q3 | 192 | 57013.385 | 3.368 | 3.37 (2.51-4.51) | <0.001 | 1.89 (1.39-2.57) | <0.001 | 1.81 (1.32-2.48) | <0.001 |
|  | Q4 | 418 | 54418.881 | 7.681 | 7.59 (5.78-9.96) | <0.001 | 3.71 (2.76-5.00) | <0.001 | 3.02 (2.19-4.15) | <0.001 |
|  | *P* for trend |  |  |  |  | <0.001 |  | <0.001 |  | <0.001 |
| METS-VF | Continuous |  |  |  | 1.42 (1.38-1.45) | <0.001 | 1.40 (1.33-1.46) | <0.001 | 1.36 (1.28-1.44) | <0.001 |
|  | Per 1-SD |  |  |  | 1.34 (1.31-1.37) | <0.001 | 1.33 (1.28-1.38) | <0.001 | 1.30 (1.23-1.37) | <0.001 |
|  | Q1 | 60 | 58281.911 | 1.029 | Reference | — | Reference | — | Reference | — |
|  | Q2 | 92 | 59098.61 | 1.557 | 1.52 (1.10-2.11) | 0.011 | 1.17 (0.84-1.63) | 0.366 | 1.17 (0.83-1.63) | 0.371 |
|  | Q3 | 194 | 57744.21 | 3.360 | 3.28 (2.46-4.39) | <0.001 | 1.90 (1.38-2.63) | <0.001 | 1.74 (1.25-2.43) | <0.001 |
|  | Q4 | 422 | 53942.302 | 7.823 | 7.52 (5.74-9.86) | <0.001 | 3.47 (2.49-4.85) | <0.001 | 2.61 (1.84-3.71) | <0.001 |
|  | *P* for trend |  |  |  |  | <0.001 |  | <0.001 |  | <0.001 |
| LAP | Continuous |  |  |  | 1.00 (1.00-1.00) | <0.001 | 1.00 (1.00-1.00) | <0.001 | 1.00 (1.00-1.00) | <0.001 |
|  | Per 1-SD |  |  |  | 1.13 (1.11-1.15) | <0.001 | 1.12 (1.09-1.15) | <0.001 | 1.09 (1.05-1.13) | <0.001 |
|  | Q1 | 63 | 60157.34 | 1.047 | Reference | — | Reference | — | Reference | — |
|  | Q2 | 121 | 57661.047 | 2.098 | 1.98 (1.46-2.68) | <0.001 | 1.45 (1.07-1.97) | 0.018 | 1.40 (1.03-1.91) | 0.032 |
|  | Q3 | 209 | 56121.256 | 3.724 | 3.49 (2.63-4.63) | <0.001 | 2.08 (1.56-2.78) | <0.001 | 1.89 (1.40-2.53) | <0.001 |
|  | Q4 | 375 | 55127.389 | 6.802 | 6.34 (4.85-8.28) | <0.001 | 3.36 (2.54-4.44) | <0.001 | 2.81 (2.07-3.82) | <0.001 |
|  | *P* for trend |  |  |  |  | <0.001 |  | <0.001 |  | <0.001 |
| CVAI | Continuous |  |  |  | 1.00 (1.00-1.00) | <0.001 | 1.00 (1.00-1.00) | <0.001 | 1.00 (1.00-1.00) | <0.001 |
|  | Per 1-SD |  |  |  | 1.04 (1.04-1.05) | <0.001 | 1.05 (1.03-1.06) | <0.001 | 1.04 (1.03-1.06) | <0.001 |
|  | Q1 | 54 | 58897.147 | 0.917 | Reference | — | Reference | — | Reference | — |
|  | Q2 | 109 | 59079.069 | 1.845 | 2.02 (1.46-2.80) | <0.001 | 1.39 (0.99-1.95) | 0.056 | 1.41 (1.00-1.98) | 0.049 |
|  | Q3 | 185 | 57303.638 | 3.228 | 3.53 (2.60-4.78) | <0.001 | 1.79 (1.27-2.52) | <0.001 | 1.64 (1.16-2.33) | 0.005 |
|  | Q4 | 420 | 53787.18 | 7.809 | 8.38 (6.31-11.13) | <0.001 | 3.56 (2.53-5.02) | <0.001 | 2.72 (1.89-3.90) | <0.001 |
|  | *P* for trend |  |  |  |  | <0.001 |  | <0.001 |  | <0.001 |
| AIP | Continuous |  |  |  | 4.18 (3.52-4.96) | <0.001 | 2.85 (2.32-3.50) | <0.001 | 2.60 (1.98-3.42) | <0.001 |
|  | Per 1-SD |  |  |  | 1.54 (1.46-1.62) | <0.001 | 1.37 (1.29-1.46) | <0.001 | 1.33 (1.23-1.45) | <0.001 |
|  | Q1 | 78 | 60292.239 | 1.294 | Reference | — | Reference | — | Reference | — |
|  | Q2 | 132 | 57195.192 | 2.308 | 1.76 (1.33-2.32) | <0.001 | 1.39 (1.05-1.84) | 0.021 | 1.31 (0.99-1.74) | 0.057 |
|  | Q3 | 217 | 55904.228 | 3.882 | 2.94 (2.27-3.80) | <0.001 | 1.94 (1.49-2.53) | <0.001 | 1.76 (1.35-2.30) | <0.001 |
|  | Q4 | 341 | 55675.374 | 6.125 | 4.63 (3.62-5.93) | <0.001 | 2.69 (2.08-3.47) | <0.001 | 2.34 (1.74-3.15) | <0.001 |
|  | *P* for trend |  |  |  |  | <0.001 |  | <0.001 |  | <0.001 |
| CHG | Continuous |  |  |  | 3.54 (3.21-3.90) | <0.001 | 2.90 (2.56-3.30) | <0.001 | 2.64 (2.22-3.13) | <0.001 |
|  | Per 1-SD |  |  |  | 1.60 (1.54-1.66) | <0.001 | 1.49 (1.42-1.56) | <0.001 | 1.43 (1.35-1.53) | <0.001 |
|  | Q1 | 69 | 58331.549 | 1.183 | Reference | — | Reference | — | Reference | — |
|  | Q2 | 114 | 58399.028 | 1.952 | 1.66 (1.23-2.24) | <0.001 | 1.19 (0.88-1.61) | 0.271 | 1.21 (0.89-1.64) | 0.226 |
|  | Q3 | 192 | 57442.456 | 3.342 | 2.84 (2.15-3.73) | <0.001 | 1.62 (1.22-2.16) | <0.001 | 1.60 (1.20-2.15) | 0.002 |
|  | Q4 | 393 | 54894 | 7.159 | 6.05 (4.68-7.81) | <0.001 | 2.94 (2.23-3.87) | <0.001 | 2.48 (1.82-3.36) | <0.001 |
|  | *P* for trend |  |  |  |  | <0.001 |  | <0.001 |  | <0.001 |
| RC | Continuous |  |  |  | 1.01 (1.01-1.01) | <0.001 | 1.01 (1.01-1.01) | <0.001 | 1.01 (1.00-1.01) | <0.001 |
|  | Per 1-SD |  |  |  | 1.19 (1.15-1.23) | <0.001 | 1.16 (1.11-1.20) | <0.001 | 1.12 (1.07-1.18) | <0.001 |
|  | Q1 | 109 | 53432.918 | 2.040 | Reference | — | Reference | — | Reference | — |
|  | Q2 | 154 | 57743.107 | 2.667 | 1.35 (1.06-1.73) | 0.017 | 1.22 (0.96-1.57) | 0.105 | 1.30 (1.02-1.67) | 0.034 |
|  | Q3 | 192 | 56781.861 | 3.381 | 1.74 (1.38-2.20) | <0.001 | 1.37 (1.09-1.74) | 0.008 | 1.40 (1.10-1.78) | 0.005 |
|  | Q4 | 313 | 61109.146 | 5.122 | 2.68 (2.15-3.33) | <0.001 | 1.85 (1.49-2.31) | <0.001 | 1.71 (1.35-2.17) | <0.001 |
|  | *P* for trend |  |  |  |  | <0.001 |  | <0.001 |  | <0.001 |

*Notes:* Incidence rates are presented per 1,000 person-years. Model 1 was unadjusted; Model 2 was adjusted for age and sex; Model 3 was further adjusted for smoking status, alcohol consumption, medication use, diabetes, dyslipidemia, hypertension, and eGFR.

Abbreviations: AIP, atherogenic index of plasma; CHG, cholesterol–high-density lipoprotein–glucose index; CI, confidence interval; CKD, chronic kidney disease; CVAI, Chinese visceral adiposity index; eGFR, estimated glomerular filtration rate; HR, hazard ratio; LAP, lipid accumulation product; METS-IR, metabolic score for insulin resistance; METS-VF, metabolic score for visceral fat; PY, person-years; RC, remnant cholesterol; SD, standard deviation; TG/HDL-C, triglyceride to high-density lipoprotein cholesterol ratio; TyG, triglyceride–glucose index; TyG-BMI, triglyceride–glucose index combined with body mass index; TyG-WC, triglyceride–glucose index combined with waist circumference; TyG-WHtR, triglyceride–glucose index combined with waist-to-height ratio

Supplementary Table 9. Associations of surrogate indices of insulin resistance with incident CKD after excluding participants receiving medications

| Exposure | Levels | Events | PY | Incidence Rate | Model 1 | | Model 2 | | Model 3 | |
| --- | --- | --- | --- | --- | --- | --- | --- | --- | --- | --- |
|  |  |  |  |  | HR (95% CI) | *P* | HR (95% CI) | *P* | HR (95% CI) | *P* |
| TyG | Continuous |  |  |  | 2.35 (2.14-2.57) | <0.001 | 1.94 (1.75-2.16) | <0.001 | 1.76 (1.53-2.02) | <0.001 |
|  | Per 1-SD |  |  |  | 1.64 (1.55-1.73) | <0.001 | 1.47 (1.38-1.56) | <0.001 | 1.38 (1.28-1.50) | <0.001 |
|  | Q1 | 101 | 56640.611 | 1.783 | Reference | — | Reference | — | Reference | — |
|  | Q2 | 136 | 52951.91 | 2.568 | 1.41 (1.09-1.82) | 0.010 | 1.18 (0.91-1.54) | 0.200 | 1.14 (0.88-1.48) | 0.310 |
|  | Q3 | 208 | 51045.643 | 4.075 | 2.20 (1.73-2.79) | <0.001 | 1.63 (1.28-2.08) | <0.001 | 1.50 (1.17-1.92) | 0.001 |
|  | Q4 | 366 | 51176.148 | 7.152 | 3.87 (3.10-4.82) | <0.001 | 2.49 (1.98-3.14) | <0.001 | 1.97 (1.51-2.57) | <0.001 |
|  | *P* for trend |  |  |  |  | <0.001 |  | <0.001 |  | <0.001 |
| TG/HDL-C | Continuous |  |  |  | 1.00 (1.00-1.00) | 0.057 | 1.00 (1.00-1.00) | 0.265 | 1.00 (1.00-1.00) | 0.687 |
|  | Per 1-SD |  |  |  | 1.02 (1.00-1.04) | 0.057 | 1.02 (0.99-1.04) | 0.265 | 1.01 (0.97-1.05) | 0.687 |
|  | Q1 | 101 | 55568.516 | 1.818 | Reference | — | Reference | — | Reference | — |
|  | Q2 | 135 | 52934.673 | 2.550 | 1.38 (1.07-1.79) | 0.014 | 1.16 (0.90-1.50) | 0.261 | 1.11 (0.86-1.45) | 0.413 |
|  | Q3 | 219 | 51965.191 | 4.214 | 2.27 (1.79-2.87) | <0.001 | 1.64 (1.29-2.09) | <0.001 | 1.52 (1.19-1.94) | <0.001 |
|  | Q4 | 356 | 51345.933 | 6.933 | 3.72 (2.98-4.64) | <0.001 | 2.37 (1.87-2.99) | <0.001 | 2.03 (1.54-2.66) | <0.001 |
|  | *P* for trend |  |  |  |  | <0.001 |  | <0.001 |  | <0.001 |
| METS-IR | Continuous |  |  |  | 1.00 (1.00-1.00) | <0.001 | 1.00 (1.00-1.00) | <0.001 | 1.00 (1.00-1.00) | 0.002 |
|  | Per 1-SD |  |  |  | 1.03 (1.02-1.04) | <0.001 | 1.03 (1.02-1.05) | <0.001 | 1.03 (1.01-1.05) | 0.002 |
|  | Q1 | 96 | 53857.124 | 1.782 | Reference | — | Reference | — | Reference | — |
|  | Q2 | 113 | 53925.681 | 2.095 | 1.18 (0.90-1.55) | 0.233 | 0.94 (0.71-1.23) | 0.639 | 0.93 (0.71-1.23) | 0.609 |
|  | Q3 | 204 | 52944.838 | 3.853 | 2.16 (1.70-2.76) | <0.001 | 1.42 (1.10-1.84) | 0.007 | 1.35 (1.04-1.75) | 0.025 |
|  | Q4 | 398 | 51086.67 | 7.791 | 4.33 (3.47-5.41) | <0.001 | 2.63 (2.06-3.37) | <0.001 | 2.23 (1.71-2.91) | <0.001 |
|  | *P* for trend |  |  |  |  | <0.001 |  | <0.001 |  | <0.001 |
| TyG-BMI | Continuous |  |  |  | 1.00 (1.00-1.00) | <0.001 | 1.00 (1.00-1.00) | <0.001 | 1.00 (1.00-1.00) | <0.001 |
|  | Per 1-SD |  |  |  | 1.02 (1.01-1.03) | <0.001 | 1.03 (1.01-1.04) | <0.001 | 1.02 (1.01-1.04) | <0.001 |
|  | Q1 | 95 | 54741.967 | 1.735 | Reference | — | Reference | — | Reference | — |
|  | Q2 | 131 | 53545.888 | 2.446 | 1.40 (1.08-1.82) | 0.012 | 1.10 (0.84-1.43) | 0.499 | 1.07 (0.82-1.40) | 0.614 |
|  | Q3 | 190 | 52558.203 | 3.615 | 2.06 (1.61-2.63) | <0.001 | 1.33 (1.03-1.73) | 0.031 | 1.23 (0.94-1.60) | 0.125 |
|  | Q4 | 395 | 50968.254 | 7.750 | 4.38 (3.50-5.48) | <0.001 | 2.61 (2.05-3.34) | <0.001 | 2.10 (1.62-2.73) | <0.001 |
|  | *P* for trend |  |  |  |  | <0.001 |  | <0.001 |  | <0.001 |
| TyG-WC | Continuous |  |  |  | 1.00 (1.00-1.01) | <0.001 | 1.00 (1.00-1.00) | <0.001 | 1.00 (1.00-1.00) | <0.001 |
|  | Per 1-SD |  |  |  | 1.79 (1.70-1.88) | <0.001 | 1.65 (1.54-1.76) | <0.001 | 1.55 (1.43-1.68) | <0.001 |
|  | Q1 | 92 | 54907.117 | 1.676 | Reference | — | Reference | — | Reference | — |
|  | Q2 | 114 | 53601.099 | 2.127 | 1.26 (0.96-1.66) | 0.100 | 1.00 (0.75-1.33) | 0.992 | 0.98 (0.74-1.30) | 0.901 |
|  | Q3 | 186 | 52796.563 | 3.523 | 2.08 (1.62-2.68) | <0.001 | 1.35 (1.02-1.78) | 0.035 | 1.27 (0.96-1.69) | 0.099 |
|  | Q4 | 419 | 50509.535 | 8.295 | 4.84 (3.86-6.06) | <0.001 | 2.80 (2.14-3.68) | <0.001 | 2.27 (1.69-3.04) | <0.001 |
|  | *P* for trend |  |  |  |  | <0.001 |  | <0.001 |  | <0.001 |
| TyG-WHtR | Continuous |  |  |  | 1.18 (1.16-1.20) | <0.001 | 1.19 (1.16-1.22) | <0.001 | 1.18 (1.15-1.22) | <0.001 |
|  | Per 1-SD |  |  |  | 1.12 (1.11-1.14) | <0.001 | 1.13 (1.11-1.15) | <0.001 | 1.12 (1.10-1.15) | <0.001 |
|  | Q1 | 96 | 54850.315 | 1.750 | Reference | — | Reference | — | Reference | — |
|  | Q2 | 114 | 53607.052 | 2.127 | 1.20 (0.92-1.58) | 0.181 | 0.96 (0.73-1.27) | 0.770 | 0.95 (0.72-1.25) | 0.708 |
|  | Q3 | 203 | 52792.395 | 3.845 | 2.17 (1.70-2.77) | <0.001 | 1.44 (1.11-1.87) | 0.006 | 1.38 (1.06-1.79) | 0.017 |
|  | Q4 | 398 | 50564.551 | 7.871 | 4.39 (3.51-5.49) | <0.001 | 2.61 (2.04-3.35) | <0.001 | 2.16 (1.65-2.83) | <0.001 |
|  | *P* for trend |  |  |  |  | <0.001 |  | <0.001 |  | <0.001 |
| METS-VF | Continuous |  |  |  | 1.39 (1.35-1.44) | <0.001 | 1.36 (1.29-1.43) | <0.001 | 1.33 (1.25-1.42) | <0.001 |
|  | Per 1-SD |  |  |  | 1.32 (1.29-1.35) | <0.001 | 1.29 (1.24-1.35) | <0.001 | 1.27 (1.21-1.34) | <0.001 |
|  | Q1 | 90 | 53483.095 | 1.683 | Reference | — | Reference | — | Reference | — |
|  | Q2 | 119 | 54529.01 | 2.182 | 1.31 (1.00-1.72) | 0.053 | 1.10 (0.83-1.46) | 0.498 | 1.07 (0.81-1.43) | 0.619 |
|  | Q3 | 199 | 53555.861 | 3.716 | 2.23 (1.74-2.86) | <0.001 | 1.54 (1.16-2.04) | 0.003 | 1.42 (1.06-1.88) | 0.017 |
|  | Q4 | 403 | 50246.346 | 8.020 | 4.72 (3.75-5.93) | <0.001 | 2.79 (2.09-3.74) | <0.001 | 2.23 (1.64-3.02) | <0.001 |
|  | *P* for trend |  |  |  |  | <0.001 |  | <0.001 |  | <0.001 |
| LAP | Continuous |  |  |  | 1.00 (1.00-1.00) | <0.001 | 1.00 (1.00-1.00) | <0.001 | 1.00 (1.00-1.00) | <0.001 |
|  | Per 1-SD |  |  |  | 1.13 (1.11-1.15) | <0.001 | 1.12 (1.10-1.14) | <0.001 | 1.10 (1.07-1.14) | <0.001 |
|  | Q1 | 103 | 55392.596 | 1.859 | Reference | — | Reference | — | Reference | — |
|  | Q2 | 123 | 53390.095 | 2.304 | 1.22 (0.94-1.59) | 0.135 | 0.99 (0.76-1.30) | 0.964 | 0.97 (0.75-1.27) | 0.846 |
|  | Q3 | 204 | 51922.939 | 3.929 | 2.07 (1.63-2.62) | <0.001 | 1.44 (1.13-1.84) | 0.004 | 1.36 (1.06-1.74) | 0.015 |
|  | Q4 | 381 | 51108.684 | 7.455 | 3.90 (3.14-4.85) | <0.001 | 2.41 (1.91-3.05) | <0.001 | 2.06 (1.59-2.68) | <0.001 |
|  | *P* for trend |  |  |  |  | <0.001 |  | <0.001 |  | <0.001 |
| CVAI | Continuous |  |  |  | 1.00 (1.00-1.00) | <0.001 | 1.00 (1.00-1.00) | <0.001 | 1.00 (1.00-1.00) | <0.001 |
|  | Per 1-SD |  |  |  | 1.04 (1.03-1.05) | <0.001 | 1.04 (1.03-1.06) | <0.001 | 1.04 (1.02-1.06) | <0.001 |
|  | Q1 | 86 | 54082.009 | 1.590 | Reference | — | Reference | — | Reference | — |
|  | Q2 | 127 | 54517.091 | 2.330 | 1.47 (1.12-1.94) | 0.006 | 1.17 (0.88-1.56) | 0.269 | 1.14 (0.85-1.52) | 0.377 |
|  | Q3 | 194 | 53014.749 | 3.659 | 2.31 (1.79-2.97) | <0.001 | 1.47 (1.10-1.97) | 0.010 | 1.34 (0.99-1.80) | 0.058 |
|  | Q4 | 404 | 50200.464 | 8.048 | 4.99 (3.95-6.29) | <0.001 | 2.77 (2.06-3.73) | <0.001 | 2.17 (1.59-2.98) | <0.001 |
|  | *P* for trend |  |  |  |  | <0.001 |  | <0.001 |  | <0.001 |
| AIP | Continuous |  |  |  | 4.01 (3.38-4.74) | <0.001 | 2.81 (2.30-3.44) | <0.001 | 2.46 (1.89-3.20) | <0.001 |
|  | Per 1-SD |  |  |  | 1.51 (1.44-1.59) | <0.001 | 1.36 (1.28-1.45) | <0.001 | 1.31 (1.21-1.42) | <0.001 |
|  | Q1 | 101 | 55568.516 | 1.818 | Reference | — | Reference | — | Reference | — |
|  | Q2 | 135 | 52934.673 | 2.550 | 1.38 (1.07-1.79) | 0.014 | 1.16 (0.90-1.50) | 0.261 | 1.11 (0.86-1.45) | 0.413 |
|  | Q3 | 219 | 51965.191 | 4.214 | 2.27 (1.79-2.87) | <0.001 | 1.64 (1.29-2.09) | <0.001 | 1.52 (1.19-1.94) | <0.001 |
|  | Q4 | 356 | 51345.933 | 6.933 | 3.72 (2.98-4.64) | <0.001 | 2.37 (1.87-2.99) | <0.001 | 2.03 (1.54-2.66) | <0.001 |
|  | *P* for trend |  |  |  |  | <0.001 |  | <0.001 |  | <0.001 |
| CHG | Continuous |  |  |  | 3.33 (3.01-3.68) | <0.001 | 2.81 (2.49-3.18) | <0.001 | 2.62 (2.23-3.07) | <0.001 |
|  | Per 1-SD |  |  |  | 1.54 (1.49-1.60) | <0.001 | 1.45 (1.39-1.52) | <0.001 | 1.41 (1.33-1.50) | <0.001 |
|  | Q1 | 91 | 53748.42 | 1.693 | Reference | — | Reference | — | Reference | — |
|  | Q2 | 132 | 53772.228 | 2.455 | 1.46 (1.12-1.91) | 0.005 | 1.17 (0.89-1.54) | 0.251 | 1.18 (0.90-1.55) | 0.227 |
|  | Q3 | 193 | 53234.643 | 3.625 | 2.15 (1.68-2.76) | <0.001 | 1.44 (1.11-1.87) | 0.006 | 1.41 (1.08-1.85) | 0.011 |
|  | Q4 | 395 | 51059.023 | 7.736 | 4.56 (3.63-5.73) | <0.001 | 2.70 (2.10-3.46) | <0.001 | 2.35 (1.77-3.10) | <0.001 |
|  | *P* for trend |  |  |  |  | <0.001 |  | <0.001 |  | <0.001 |
| RC | Continuous |  |  |  | 1.01 (1.01-1.01) | <0.001 | 1.01 (1.01-1.01) | <0.001 | 1.01 (1.01-1.01) | <0.001 |
|  | Per 1-SD |  |  |  | 1.20 (1.17-1.23) | <0.001 | 1.18 (1.14-1.22) | <0.001 | 1.15 (1.10-1.20) | <0.001 |
|  | Q1 | 108 | 48327.543 | 2.235 | Reference | — | Reference | — | Reference | — |
|  | Q2 | 169 | 54183.997 | 3.119 | 1.45 (1.14-1.84) | 0.003 | 1.36 (1.06-1.73) | 0.014 | 1.35 (1.06-1.73) | 0.014 |
|  | Q3 | 200 | 53618.571 | 3.730 | 1.77 (1.40-2.23) | <0.001 | 1.49 (1.18-1.89) | <0.001 | 1.42 (1.12-1.80) | 0.004 |
|  | Q4 | 334 | 55684.202 | 5.998 | 2.89 (2.33-3.60) | <0.001 | 2.18 (1.75-2.72) | <0.001 | 1.89 (1.49-2.40) | <0.001 |
|  | *P* for trend |  |  |  |  | <0.001 |  | <0.001 |  | <0.001 |

*Notes:* Incidence rates are presented per 1,000 person-years. Model 1 was unadjusted; Model 2 was adjusted for age and sex; Model 3 was further adjusted for smoking status, alcohol consumption, medication use, diabetes, dyslipidemia, hypertension, and eGFR.

Abbreviations: AIP, atherogenic index of plasma; CHG, cholesterol–high-density lipoprotein–glucose index; CI, confidence interval; CKD, chronic kidney disease; CVAI, Chinese visceral adiposity index; eGFR, estimated glomerular filtration rate; HR, hazard ratio; LAP, lipid accumulation product; METS-IR, metabolic score for insulin resistance; METS-VF, metabolic score for visceral fat; PY, person-years; RC, remnant cholesterol; SD, standard deviation; TG/HDL-C, triglyceride to high-density lipoprotein cholesterol ratio; TyG, triglyceride–glucose index; TyG-BMI, triglyceride–glucose index combined with body mass index; TyG-WC, triglyceride–glucose index combined with waist circumference; TyG-WHtR, triglyceride–glucose index combined with waist-to-height ratio

Supplementary Table 10. Associations of surrogate indices of insulin resistance with incident CKD using a more stringent definition

| Exposure | Levels | Events | PY | Incidence Rate | Model 1 | | Model 2 | | Model 3 | |
| --- | --- | --- | --- | --- | --- | --- | --- | --- | --- | --- |
|  |  |  |  |  | HR (95% CI) | *P* | HR (95% CI) | *P* | HR (95% CI) | *P* |
| TyG | Continuous |  |  |  | 2.84 (2.46-3.28) | <0.001 | 2.49 (2.11-2.93) | <0.001 | 2.04 (1.64-2.53) | <0.001 |
|  | Per 1-SD |  |  |  | 1.86 (1.71-2.02) | <0.001 | 1.72 (1.56-1.89) | <0.001 | 1.53 (1.34-1.74) | <0.001 |
|  | Q1 | 26 | 63908.843 | 0.407 | Reference | — | Reference | — | Reference | — |
|  | Q2 | 42 | 59679.463 | 0.704 | 1.78 (1.09-2.91) | 0.020 | 1.42 (0.87-2.33) | 0.163 | 1.33 (0.81-2.19) | 0.252 |
|  | Q3 | 54 | 58558.808 | 0.922 | 2.37 (1.48-3.78) | <0.001 | 1.66 (1.03-2.67) | 0.038 | 1.46 (0.90-2.36) | 0.126 |
|  | Q4 | 137 | 59073.127 | 2.319 | 5.93 (3.89-9.01) | <0.001 | 3.62 (2.33-5.60) | <0.001 | 2.47 (1.49-4.08) | <0.001 |
|  | *P* for trend |  |  |  |  | <0.001 |  | <0.001 |  | <0.001 |
| TG/HDL-C | Continuous |  |  |  | 1.00 (1.00-1.00) | 0.085 | 1.00 (1.00-1.00) | 0.107 | 1.00 (1.00-1.00) | 0.229 |
|  | Per 1-SD |  |  |  | 1.03 (1.00-1.06) | 0.085 | 1.03 (0.99-1.06) | 0.107 | 1.03 (0.98-1.08) | 0.229 |
|  | Q1 | 33 | 62605.651 | 0.527 | Reference | — | Reference | — | Reference | — |
|  | Q2 | 50 | 59908.612 | 0.835 | 1.61 (1.04-2.51) | 0.033 | 1.33 (0.85-2.07) | 0.210 | 1.21 (0.78-1.89) | 0.390 |
|  | Q3 | 63 | 59146.516 | 1.065 | 2.07 (1.36-3.16) | <0.001 | 1.50 (0.97-2.30) | 0.065 | 1.25 (0.81-1.94) | 0.310 |
|  | Q4 | 113 | 59559.463 | 1.897 | 3.68 (2.50-5.43) | <0.001 | 2.49 (1.66-3.73) | <0.001 | 1.70 (1.05-2.76) | 0.030 |
|  | *P* for trend |  |  |  |  | <0.001 |  | <0.001 |  | 0.036 |
| METS-IR | Continuous |  |  |  | 1.00 (1.00-1.00) | 0.002 | 1.00 (1.00-1.00) | 0.002 | 1.00 (1.00-1.00) | 0.019 |
|  | Per 1-SD |  |  |  | 1.03 (1.01-1.05) | 0.002 | 1.04 (1.01-1.06) | 0.002 | 1.03 (1.01-1.06) | 0.019 |
|  | Q1 | 26 | 60627.796 | 0.429 | Reference | — | Reference | — | Reference | — |
|  | Q2 | 42 | 60582.933 | 0.693 | 1.61 (0.99-2.63) | 0.056 | 1.22 (0.74-2.01) | 0.428 | 1.20 (0.73-1.98) | 0.468 |
|  | Q3 | 60 | 60681.083 | 0.989 | 2.29 (1.45-3.63) | <0.001 | 1.48 (0.91-2.40) | 0.111 | 1.26 (0.77-2.07) | 0.351 |
|  | Q4 | 131 | 59328.429 | 2.208 | 5.17 (3.39-7.87) | <0.001 | 3.32 (2.10-5.23) | <0.001 | 2.18 (1.33-3.59) | 0.002 |
|  | *P* for trend |  |  |  |  | <0.001 |  | <0.001 |  | <0.001 |
| TyG-BMI | Continuous |  |  |  | 1.00 (1.00-1.00) | 0.002 | 1.00 (1.00-1.00) | 0.002 | 1.00 (1.00-1.00) | 0.027 |
|  | Per 1-SD |  |  |  | 1.02 (1.01-1.04) | 0.002 | 1.03 (1.01-1.04) | 0.002 | 1.03 (1.00-1.05) | 0.027 |
|  | Q1 | 20 | 61656.943 | 0.324 | Reference | — | Reference | — | Reference | — |
|  | Q2 | 45 | 60354.952 | 0.746 | 2.32 (1.37-3.92) | 0.002 | 1.71 (1.00-2.92) | 0.048 | 1.66 (0.97-2.84) | 0.063 |
|  | Q3 | 66 | 59887.288 | 1.102 | 3.43 (2.08-5.66) | <0.001 | 2.12 (1.26-3.57) | 0.005 | 1.80 (1.05-3.06) | 0.031 |
|  | Q4 | 128 | 59321.058 | 2.158 | 6.75 (4.21-10.81) | <0.001 | 4.10 (2.49-6.75) | <0.001 | 2.62 (1.53-4.49) | <0.001 |
|  | *P* for trend |  |  |  |  | <0.001 |  | <0.001 |  | <0.001 |
| TyG-WC | Continuous |  |  |  | 1.00 (1.00-1.00) | <0.001 | 1.00 (1.00-1.00) | <0.001 | 1.00 (1.00-1.00) | <0.001 |
|  | Per 1-SD |  |  |  | 1.16 (1.14-1.19) | <0.001 | 1.16 (1.13-1.20) | <0.001 | 1.13 (1.09-1.18) | <0.001 |
|  | Q1 | 19 | 61768.157 | 0.308 | Reference | — | Reference | — | Reference | — |
|  | Q2 | 39 | 60599.215 | 0.644 | 2.10 (1.22-3.64) | 0.008 | 1.62 (0.93-2.84) | 0.089 | 1.59 (0.91-2.80) | 0.105 |
|  | Q3 | 66 | 60415.329 | 1.092 | 3.57 (2.14-5.95) | <0.001 | 2.36 (1.36-4.08) | 0.002 | 2.04 (1.16-3.59) | 0.013 |
|  | Q4 | 135 | 58437.54 | 2.310 | 7.69 (4.76-12.43) | <0.001 | 4.81 (2.79-8.28) | <0.001 | 3.04 (1.66-5.39) | <0.001 |
|  | *P* for trend |  |  |  |  | <0.001 |  | <0.001 |  | <0.001 |
| TyG-WHtR | Continuous |  |  |  | 1.17 (1.14-1.21) | <0.001 | 1.19 (1.14-1.23) | <0.001 | 1.16 (1.11-1.22) | <0.001 |
|  | Per 1-SD |  |  |  | 1.13 (1.10-1.15) | <0.001 | 1.13 (1.10-1.16) | <0.001 | 1.12 (1.08-1.15) | <0.001 |
|  | Q1 | 19 | 61834.275 | 0.307 | Reference | — | Reference | — | Reference | — |
|  | Q2 | 34 | 60578.172 | 0.561 | 1.84 (1.05-3.23) | 0.033 | 1.40 (0.79-2.48) | 0.244 | 1.41 (0.79-2.49) | 0.241 |
|  | Q3 | 63 | 60126.422 | 1.048 | 3.44 (2.06-5.75) | <0.001 | 2.16 (1.26-3.70) | 0.005 | 1.97 (1.14-3.43) | 0.016 |
|  | Q4 | 143 | 58681.372 | 2.437 | 8.12 (5.03-13.10) | <0.001 | 4.47 (2.66-7.51) | <0.001 | 2.99 (1.73-5.33) | <0.001 |
|  | *P* for trend |  |  |  |  | <0.001 |  | <0.001 |  | <0.001 |
| METS-VF | Continuous |  |  |  | 1.41 (1.34-1.47) | <0.001 | 1.39 (1.28-1.50) | <0.001 | 1.34 (1.20-1.50) | <0.001 |
|  | Per 1-SD |  |  |  | 1.34 (1.29-1.39) | <0.001 | 1.32 (1.24-1.41) | <0.001 | 1.28 (1.17-1.41) | <0.001 |
|  | Q1 | 21 | 60466.957 | 0.347 | Reference | — | Reference | — | Reference | — |
|  | Q2 | 36 | 61631.491 | 0.584 | 1.66 (0.97-2.85) | 0.064 | 1.31 (0.76-2.27) | 0.338 | 1.29 (0.74-2.25) | 0.361 |
|  | Q3 | 66 | 60817.078 | 1.085 | 3.09 (1.89-5.06) | <0.001 | 1.98 (1.15-3.41) | 0.013 | 1.66 (0.95-2.91) | 0.075 |
|  | Q4 | 136 | 58304.716 | 2.333 | 6.78 (4.28-10.73) | <0.001 | 3.38 (1.92-5.96) | <0.001 | 2.15 (1.18-3.93) | 0.013 |
|  | *P* for trend |  |  |  |  | <0.001 |  | <0.001 |  | 0.006 |
| LAP | Continuous |  |  |  | 1.00 (1.00-1.00) | <0.001 | 1.00 (1.00-1.00) | <0.001 | 1.00 (1.00-1.00) | <0.001 |
|  | Per 1-SD |  |  |  | 1.15 (1.12-1.18) | <0.001 | 1.15 (1.11-1.19) | <0.001 | 1.12 (1.07-1.17) | <0.001 |
|  | Q1 | 20 | 62530.914 | 0.320 | Reference | — | Reference | — | Reference | — |
|  | Q2 | 49 | 60102.777 | 0.815 | 2.60 (1.54-4.37) | <0.001 | 1.94 (1.15-3.28) | 0.013 | 1.82 (1.08-3.09) | 0.026 |
|  | Q3 | 68 | 59553.839 | 1.142 | 3.65 (2.22-6.00) | <0.001 | 2.28 (1.37-3.80) | 0.002 | 1.91 (1.13-3.22) | 0.016 |
|  | Q4 | 122 | 59032.711 | 2.067 | 6.65 (4.14-10.67) | <0.001 | 3.85 (2.35-6.30) | <0.001 | 2.56 (1.49-4.40) | <0.001 |
|  | *P* for trend |  |  |  |  | <0.001 |  | <0.001 |  | 0.001 |
| CVAI | Continuous |  |  |  | 1.00 (1.00-1.00) | <0.001 | 1.00 (1.00-1.00) | <0.001 | 1.00 (1.00-1.00) | 0.006 |
|  | Per 1-SD |  |  |  | 1.04 (1.03-1.06) | <0.001 | 1.04 (1.02-1.07) | <0.001 | 1.04 (1.01-1.07) | 0.006 |
|  | Q1 | 16 | 61079.104 | 0.262 | Reference | — | Reference | — | Reference | — |
|  | Q2 | 41 | 61614.782 | 0.665 | 2.52 (1.41-4.49) | 0.002 | 1.82 (1.01-3.30) | 0.048 | 1.83 (1.00-3.32) | 0.048 |
|  | Q3 | 70 | 60408.455 | 1.159 | 4.40 (2.55-7.57) | <0.001 | 2.58 (1.42-4.71) | 0.002 | 2.23 (1.20-4.14) | 0.011 |
|  | Q4 | 132 | 58117.901 | 2.271 | 8.81 (5.24-14.79) | <0.001 | 4.44 (2.41-8.21) | <0.001 | 2.88 (1.50-5.53) | 0.001 |
|  | *P* for trend |  |  |  |  | <0.001 |  | <0.001 |  | 0.001 |
| AIP | Continuous |  |  |  | 4.37 (3.24-5.90) | <0.001 | 3.58 (2.52-5.07) | <0.001 | 2.76 (1.72-4.42) | <0.001 |
|  | Per 1-SD |  |  |  | 1.56 (1.43-1.71) | <0.001 | 1.47 (1.32-1.63) | <0.001 | 1.36 (1.18-1.57) | <0.001 |
|  | Q1 | 33 | 62605.651 | 0.527 | Reference | — | Reference | — | Reference | — |
|  | Q2 | 50 | 59908.612 | 0.835 | 1.61 (1.04-2.51) | 0.033 | 1.33 (0.85-2.07) | 0.210 | 1.21 (0.78-1.89) | 0.390 |
|  | Q3 | 63 | 59146.516 | 1.065 | 2.07 (1.36-3.16) | <0.001 | 1.50 (0.97-2.30) | 0.065 | 1.25 (0.81-1.94) | 0.310 |
|  | Q4 | 113 | 59559.463 | 1.897 | 3.68 (2.50-5.43) | <0.001 | 2.49 (1.66-3.73) | <0.001 | 1.70 (1.05-2.76) | 0.030 |
|  | *P* for trend |  |  |  |  | <0.001 |  | <0.001 |  | 0.036 |
| CHG | Continuous |  |  |  | 4.19 (3.60-4.87) | <0.001 | 3.82 (3.18-4.59) | <0.001 | 3.05 (2.34-3.97) | <0.001 |
|  | Per 1-SD |  |  |  | 1.71 (1.61-1.81) | <0.001 | 1.65 (1.54-1.77) | <0.001 | 1.52 (1.37-1.67) | <0.001 |
|  | Q1 | 28 | 60461.416 | 0.463 | Reference | — | Reference | — | Reference | — |
|  | Q2 | 30 | 61111.615 | 0.491 | 1.05 (0.63-1.76) | 0.854 | 0.79 (0.47-1.34) | 0.384 | 0.82 (0.48-1.38) | 0.447 |
|  | Q3 | 65 | 60533.179 | 1.074 | 2.31 (1.48-3.60) | <0.001 | 1.48 (0.93-2.36) | 0.096 | 1.41 (0.88-2.28) | 0.154 |
|  | Q4 | 136 | 59114.031 | 2.301 | 4.97 (3.31-7.46) | <0.001 | 2.78 (1.78-4.34) | <0.001 | 1.97 (1.19-3.26) | 0.008 |
|  | *P* for trend |  |  |  |  | <0.001 |  | <0.001 |  | <0.001 |
| RC | Continuous |  |  |  | 1.01 (1.01-1.01) | <0.001 | 1.01 (1.01-1.01) | <0.001 | 1.01 (1.00-1.01) | <0.001 |
|  | Per 1-SD |  |  |  | 1.21 (1.16-1.26) | <0.001 | 1.20 (1.13-1.27) | <0.001 | 1.16 (1.08-1.24) | <0.001 |
|  | Q1 | 43 | 55439.368 | 0.776 | Reference | — | Reference | — | Reference | — |
|  | Q2 | 49 | 60576.776 | 0.809 | 1.00 (0.66-1.50) | 0.996 | 0.89 (0.59-1.33) | 0.561 | 1.00 (0.66-1.51) | 0.996 |
|  | Q3 | 63 | 59726.971 | 1.055 | 1.28 (0.87-1.88) | 0.219 | 0.96 (0.65-1.41) | 0.824 | 0.97 (0.65-1.43) | 0.866 |
|  | Q4 | 104 | 65477.126 | 1.588 | 1.88 (1.32-2.69) | <0.001 | 1.25 (0.87-1.79) | 0.231 | 0.99 (0.67-1.47) | 0.953 |
|  | *P* for trend |  |  |  |  | <0.001 |  | 0.098 |  | 0.924 |

*Notes:* Incidence rates are presented per 1,000 person-years. Model 1 was unadjusted; Model 2 was adjusted for age and sex; Model 3 was further adjusted for smoking status, alcohol consumption, medication use, diabetes, dyslipidemia, hypertension, and eGFR. A stricter definition of CKD was defined as having at least two consecutive eGFR values <45 mL/min/1.73 m², with an interval of ≥3 months between measurements.

Abbreviations: AIP, atherogenic index of plasma; CHG, cholesterol–high-density lipoprotein–glucose index; CI, confidence interval; CKD, chronic kidney disease; CVAI, Chinese visceral adiposity index; eGFR, estimated glomerular filtration rate; HR, hazard ratio; LAP, lipid accumulation product; METS-IR, metabolic score for insulin resistance; METS-VF, metabolic score for visceral fat; PY, person-years; RC, remnant cholesterol; SD, standard deviation; TG/HDL-C, triglyceride to high-density lipoprotein cholesterol ratio; TyG, triglyceride–glucose index; TyG-BMI, triglyceride–glucose index combined with body mass index; TyG-WC, triglyceride–glucose index combined with waist circumference; TyG-WHtR, triglyceride–glucose index combined with waist-to-height ratio

Supplementary Table 11. Associations of surrogate indices of insulin resistance with incident CKD after further adjustment for for additional covariates

| Exposure | Levels | Events | PY | Incidence Rate | Model 1 | | Model 2 | | Model 3 | |
| --- | --- | --- | --- | --- | --- | --- | --- | --- | --- | --- |
|  |  |  |  |  | HR (95% CI) | *P* | HR (95% CI) | *P* | HR (95% CI) | *P* |
| TyG | Continuous |  |  |  | 2.33 (2.16-2.52) | <0.001 | 1.88 (1.72-2.05) | <0.001 | 1.33 (1.17-1.53) | <0.001 |
|  | Per 1-SD |  |  |  | 1.65 (1.58-1.73) | <0.001 | 1.45 (1.38-1.53) | <0.001 | 1.19 (1.10-1.29) | <0.001 |
|  | Q1 | 121 | 61683.211 | 1.962 | Reference | — | Reference | — | Reference | — |
|  | Q2 | 181 | 57366.519 | 3.155 | 1.57 (1.25-1.98) | <0.001 | 1.26 (1.00-1.58) | 0.054 | 1.19 (0.94-1.50) | 0.146 |
|  | Q3 | 287 | 55555.479 | 5.166 | 2.54 (2.05-3.14) | <0.001 | 1.75 (1.41-2.17) | <0.001 | 1.54 (1.23-1.91) | <0.001 |
|  | Q4 | 503 | 55156.425 | 9.120 | 4.48 (3.68-5.47) | <0.001 | 2.62 (2.13-3.23) | <0.001 | 1.67 (1.31-2.13) | <0.001 |
|  | *P* for trend |  |  |  |  | <0.001 |  | <0.001 |  | <0.001 |
| TG/HDL-C | Continuous |  |  |  | 1.00 (1.00-1.00) | 0.028 | 1.00 (1.00-1.00) | 0.193 | 1.00 (1.00-1.00) | 0.891 |
|  | Per 1-SD |  |  |  | 1.02 (1.00-1.04) | 0.028 | 1.02 (0.99-1.04) | 0.193 | 1.00 (0.95-1.06) | 0.891 |
|  | Q1 | 131 | 60539.696 | 2.164 | Reference | — | Reference | — | Reference | — |
|  | Q2 | 188 | 57422.139 | 3.274 | 1.49 (1.19-1.86) | <0.001 | 1.20 (0.96-1.50) | 0.118 | 1.11 (0.88-1.39) | 0.379 |
|  | Q3 | 303 | 56073.774 | 5.404 | 2.44 (1.99-2.99) | <0.001 | 1.65 (1.34-2.04) | <0.001 | 1.42 (1.15-1.76) | 0.001 |
|  | Q4 | 470 | 55726.024 | 8.434 | 3.80 (3.13-4.62) | <0.001 | 2.26 (1.85-2.77) | <0.001 | 1.73 (1.36-2.20) | <0.001 |
|  | *P* for trend |  |  |  |  | <0.001 |  | <0.001 |  | <0.001 |
| METS-IR | Continuous |  |  |  | 1.00 (1.00-1.00) | <0.001 | 1.00 (1.00-1.00) | <0.001 | 1.00 (1.00-1.00) | <0.001 |
|  | Per 1-SD |  |  |  | 1.03 (1.02-1.04) | <0.001 | 1.03 (1.02-1.05) | <0.001 | 1.03 (1.01-1.05) | <0.001 |
|  | Q1 | 106 | 58801.937 | 1.803 | Reference | — | Reference | — | Reference | — |
|  | Q2 | 157 | 58414.936 | 2.688 | 1.49 (1.17-1.91) | 0.001 | 1.10 (0.86-1.41) | 0.452 | 1.06 (0.83-1.37) | 0.627 |
|  | Q3 | 278 | 57653.956 | 4.822 | 2.67 (2.14-3.34) | <0.001 | 1.59 (1.25-2.01) | <0.001 | 1.39 (1.09-1.77) | 0.008 |
|  | Q4 | 551 | 54890.804 | 10.038 | 5.50 (4.47-6.77) | <0.001 | 3.04 (2.43-3.82) | <0.001 | 2.12 (1.66-2.72) | <0.001 |
|  | *P* for trend |  |  |  |  | <0.001 |  | <0.001 |  | <0.001 |
| TyG-BMI | Continuous |  |  |  | 1.00 (1.00-1.00) | <0.001 | 1.00 (1.00-1.00) | <0.001 | 1.00 (1.00-1.00) | <0.001 |
|  | Per 1-SD |  |  |  | 1.02 (1.02-1.03) | <0.001 | 1.03 (1.02-1.04) | <0.001 | 1.02 (1.01-1.04) | <0.001 |
|  | Q1 | 106 | 59706.722 | 1.775 | Reference | — | Reference | — | Reference | — |
|  | Q2 | 167 | 58248.746 | 2.867 | 1.60 (1.26-2.05) | <0.001 | 1.16 (0.91-1.49) | 0.237 | 1.11 (0.87-1.43) | 0.407 |
|  | Q3 | 267 | 56831.356 | 4.698 | 2.61 (2.09-3.27) | <0.001 | 1.52 (1.20-1.92) | <0.001 | 1.33 (1.04-1.70) | 0.021 |
|  | Q4 | 552 | 54974.809 | 10.041 | 5.54 (4.50-6.82) | <0.001 | 3.00 (2.40-3.75) | <0.001 | 2.03 (1.59-2.60) | <0.001 |
|  | *P* for trend |  |  |  |  | <0.001 |  | <0.001 |  | <0.001 |
| TyG-WC | Continuous |  |  |  | 1.00 (1.00-1.00) | <0.001 | 1.00 (1.00-1.00) | <0.001 | 1.00 (1.00-1.00) | <0.001 |
|  | Per 1-SD |  |  |  | 1.15 (1.14-1.17) | <0.001 | 1.16 (1.14-1.17) | <0.001 | 1.12 (1.09-1.15) | <0.001 |
|  | Q1 | 97 | 59901.645 | 1.619 | Reference | — | Reference | — | Reference | — |
|  | Q2 | 148 | 58437.014 | 2.533 | 1.55 (1.20-2.01) | <0.001 | 1.14 (0.88-1.49) | 0.329 | 1.09 (0.84-1.43) | 0.504 |
|  | Q3 | 274 | 57334.262 | 4.779 | 2.93 (2.32-3.69) | <0.001 | 1.68 (1.30-2.17) | <0.001 | 1.47 (1.13-1.91) | 0.004 |
|  | Q4 | 573 | 54088.711 | 10.594 | 6.37 (5.14-7.90) | <0.001 | 3.27 (2.54-4.21) | <0.001 | 2.14 (1.63-2.82) | <0.001 |
|  | *P* for trend |  |  |  |  | <0.001 |  | <0.001 |  | <0.001 |
| TyG-WHtR | Continuous |  |  |  | 1.17 (1.15-1.18) | <0.001 | 1.18 (1.15-1.20) | <0.001 | 1.14 (1.11-1.18) | <0.001 |
|  | Per 1-SD |  |  |  | 1.12 (1.11-1.13) | <0.001 | 1.13 (1.11-1.14) | <0.001 | 1.10 (1.08-1.13) | <0.001 |
|  | Q1 | 105 | 59784.415 | 1.756 | Reference | — | Reference | — | Reference | — |
|  | Q2 | 148 | 58387.201 | 2.535 | 1.43 (1.11-1.84) | 0.005 | 1.06 (0.82-1.37) | 0.652 | 1.04 (0.80-1.34) | 0.776 |
|  | Q3 | 276 | 57143.351 | 4.830 | 2.72 (2.17-3.41) | <0.001 | 1.61 (1.27-2.05) | <0.001 | 1.47 (1.15-1.88) | 0.002 |
|  | Q4 | 563 | 54446.666 | 10.34 | 5.74 (4.66-7.07) | <0.001 | 3.01 (2.39-3.79) | <0.001 | 2.07 (1.61-2.67) | <0.001 |
|  | *P* for trend |  |  |  |  | <0.001 |  | <0.001 |  | <0.001 |
| METS-VF | Continuous |  |  |  | 1.41 (1.37-1.44) | <0.001 | 1.38 (1.32-1.44) | <0.001 | 1.33 (1.25-1.41) | <0.001 |
|  | Per 1-SD |  |  |  | 1.34 (1.31-1.36) | <0.001 | 1.31 (1.27-1.36) | <0.001 | 1.27 (1.21-1.34) | <0.001 |
|  | Q1 | 101 | 58582.745 | 1.724 | Reference | — | Reference | — | Reference | — |
|  | Q2 | 142 | 59368.315 | 2.392 | 1.40 (1.08-1.81) | 0.01 | 1.10 (0.85-1.43) | 0.467 | 1.06 (0.81-1.38) | 0.663 |
|  | Q3 | 280 | 57858.447 | 4.839 | 2.82 (2.25-3.54) | <0.001 | 1.73 (1.34-2.23) | <0.001 | 1.49 (1.15-1.94) | 0.003 |
|  | Q4 | 569 | 53952.126 | 10.546 | 6.03 (4.88-7.45) | <0.001 | 3.05 (2.34-3.99) | <0.001 | 2.06 (1.56-2.74) | <0.001 |
|  | *P* for trend |  |  |  |  | <0.001 |  | <0.001 |  | <0.001 |
| LAP | Continuous |  |  |  | 1.00 (1.00-1.00) | <0.001 | 1.00 (1.00-1.00) | <0.001 | 1.00 (1.00-1.00) | <0.001 |
|  | Per 1-SD |  |  |  | 1.13 (1.11-1.15) | <0.001 | 1.12 (1.09-1.14) | <0.001 | 1.06 (1.03-1.09) | <0.001 |
|  | Q1 | 122 | 60398.757 | 2.020 | Reference | — | Reference | — | Reference | — |
|  | Q2 | 158 | 57893.788 | 2.729 | 1.33 (1.05-1.68) | 0.018 | 1.01 (0.79-1.28) | 0.956 | 0.95 (0.74-1.20) | 0.647 |
|  | Q3 | 301 | 56372.536 | 5.339 | 2.59 (2.10-3.19) | <0.001 | 1.62 (1.30-2.01) | <0.001 | 1.41 (1.13-1.76) | 0.003 |
|  | Q4 | 511 | 55096.552 | 9.275 | 4.46 (3.66-5.44) | <0.001 | 2.48 (2.01-3.06) | <0.001 | 1.84 (1.46-2.33) | <0.001 |
|  | *P* for trend |  |  |  |  | <0.001 |  | <0.001 |  | <0.001 |
| CVAI | Continuous |  |  |  | 1.00 (1.00-1.00) | <0.001 | 1.00 (1.00-1.00) | <0.001 | 1.00 (1.00-1.00) | <0.001 |
|  | Per 1-SD |  |  |  | 1.04 (1.04-1.05) | <0.001 | 1.05 (1.03-1.06) | <0.001 | 1.04 (1.03-1.06) | <0.001 |
|  | Q1 | 95 | 59173.931 | 1.605 | Reference | — | Reference | — | Reference | — |
|  | Q2 | 160 | 59387.194 | 2.694 | 1.68 (1.31-2.17) | <0.001 | 1.22 (0.94-1.59) | 0.142 | 1.21 (0.92-1.57) | 0.168 |
|  | Q3 | 259 | 57492.186 | 4.505 | 2.81 (2.22-3.55) | <0.001 | 1.55 (1.18-2.02) | 0.002 | 1.35 (1.03-1.78) | 0.032 |
|  | Q4 | 578 | 53708.322 | 10.762 | 6.57 (5.29-8.16) | <0.001 | 3.10 (2.37-4.07) | <0.001 | 2.18 (1.63-2.90) | <0.001 |
|  | *P* for trend |  |  |  |  | <0.001 |  | <0.001 |  | <0.001 |
| AIP | Continuous |  |  |  | 3.88 (3.34-4.50) | <0.001 | 2.61 (2.19-3.12) | <0.001 | 1.93 (1.52-2.45) | <0.001 |
|  | Per 1-SD |  |  |  | 1.50 (1.44-1.57) | <0.001 | 1.34 (1.27-1.41) | <0.001 | 1.22 (1.14-1.31) | <0.001 |
|  | Q1 | 131 | 60539.696 | 2.164 | Reference | — | Reference | — | Reference | — |
|  | Q2 | 188 | 57422.139 | 3.274 | 1.49 (1.19-1.86) | <0.001 | 1.20 (0.96-1.50) | 0.118 | 1.11 (0.88-1.39) | 0.379 |
|  | Q3 | 303 | 56073.774 | 5.404 | 2.44 (1.99-2.99) | <0.001 | 1.65 (1.34-2.04) | <0.001 | 1.42 (1.15-1.76) | 0.001 |
|  | Q4 | 470 | 55726.024 | 8.434 | 3.80 (3.13-4.62) | <0.001 | 2.26 (1.85-2.77) | <0.001 | 1.73 (1.36-2.20) | <0.001 |
|  | *P* for trend |  |  |  |  | <0.001 |  | <0.001 |  | <0.001 |
| CHG | Continuous |  |  |  | 3.36 (3.09-3.66) | <0.001 | 2.74 (2.45-3.06) | <0.001 | 1.90 (1.55-2.33) | <0.001 |
|  | Per 1-SD |  |  |  | 1.57 (1.52-1.62) | <0.001 | 1.46 (1.40-1.52) | <0.001 | 1.27 (1.18-1.37) | <0.001 |
|  | Q1 | 112 | 58607.06 | 1.911 | Reference | — | Reference | — | Reference | — |
|  | Q2 | 168 | 58654.855 | 2.864 | 1.51 (1.19-1.92) | <0.001 | 1.11 (0.87-1.42) | 0.384 | 1.10 (0.86-1.41) | 0.438 |
|  | Q3 | 279 | 57569.362 | 4.846 | 2.54 (2.04-3.17) | <0.001 | 1.53 (1.21-1.92) | <0.001 | 1.39 (1.10-1.77) | 0.006 |
|  | Q4 | 533 | 54930.356 | 9.703 | 5.06 (4.12-6.20) | <0.001 | 2.62 (2.10-3.27) | <0.001 | 1.76 (1.36-2.27) | <0.001 |
|  | *P* for trend |  |  |  |  | <0.001 |  | <0.001 |  | <0.001 |
| RC | Continuous |  |  |  | 1.01 (1.01-1.01) | <0.001 | 1.01 (1.01-1.01) | <0.001 | 1.00 (1.00-1.01) | <0.001 |
|  | Per 1-SD |  |  |  | 1.20 (1.17-1.22) | <0.001 | 1.17 (1.13-1.21) | <0.001 | 1.09 (1.05-1.13) | <0.001 |
|  | Q1 | 152 | 53531.621 | 2.839 | Reference | — | Reference | — | Reference | — |
|  | Q2 | 228 | 57889.543 | 3.939 | 1.44 (1.17-1.77) | <0.001 | 1.32 (1.08-1.62) | 0.008 | 1.38 (1.12-1.69) | 0.002 |
|  | Q3 | 260 | 56941.848 | 4.566 | 1.70 (1.39-2.08) | <0.001 | 1.38 (1.13-1.69) | 0.002 | 1.35 (1.10-1.65) | 0.004 |
|  | Q4 | 452 | 61398.621 | 7.362 | 2.79 (2.32-3.35) | <0.001 | 2.00 (1.66-2.41) | <0.001 | 1.73 (1.41-2.12) | <0.001 |
|  | *P* for trend |  |  |  |  | <0.001 |  | <0.001 |  | <0.001 |

*Notes:* Incidence rates are presented per 1,000 person-years. Model 1 was unadjusted; Model 2 was adjusted for age and sex; Model 3 was further adjusted for smoking status, alcohol consumption, medication use, diabetes, dyslipidemia, hypertension, SBP, FPG, absolute monocyte count, and eGFR.

Abbreviations: AIP, atherogenic index of plasma; CHG, cholesterol–high-density lipoprotein–glucose index; CI, confidence interval; CKD, chronic kidney disease; CVAI, Chinese visceral adiposity index; eGFR, estimated glomerular filtration rate; FPG, fasting plasma glucose; HR, hazard ratio; LAP, lipid accumulation product; METS-IR, metabolic score for insulin resistance; METS-VF, metabolic score for visceral fat; PY, person-years; RC, remnant cholesterol; SBP, systolic blood pressure; SD, standard deviation; TG/HDL-C, triglyceride to high-density lipoprotein cholesterol ratio; TyG, triglyceride–glucose index; TyG-BMI, triglyceride–glucose index combined with body mass index; TyG-WC, triglyceride–glucose index combined with waist circumference; TyG-WHtR, triglyceride–glucose index combined with waist-to-height ratio

Supplementary Table 12. Associations of surrogate indices of insulin resistance with incident CKD after excluding participants with BMI ≥28 kg/m²

| Exposure | Levels | Events | PY | Incidence  Rate | Model 1 | | Model 2 | | Model 3 | |
| --- | --- | --- | --- | --- | --- | --- | --- | --- | --- | --- |
|  |  |  |  |  | HR (95% CI) | *P* | HR (95% CI) | *P* | HR (95% CI) | *P* |
| TyG | Continuous |  |  |  | 2.26 (2.04-2.49) | <0.001 | 1.72 (1.54-1.93) | <0.001 | 1.56 (1.35-1.82) | <0.001 |
|  | Per 1-SD |  |  |  | 1.59 (1.50-1.68) | <0.001 | 1.36 (1.28-1.46) | <0.001 | 1.29 (1.18-1.41) | <0.001 |
|  | Q1 | 98 | 52941.722 | 1.851 | Reference | — | Reference | — | Reference | — |
|  | Q2 | 138 | 49479.735 | 2.789 | 1.47 (1.14-1.91) | 0.003 | 1.19 (0.92-1.54) | 0.195 | 1.16 (0.89-1.51) | 0.260 |
|  | Q3 | 185 | 47715.213 | 3.877 | 2.02 (1.58-2.58) | <0.001 | 1.38 (1.08-1.78) | 0.011 | 1.29 (1.01-1.66) | 0.046 |
|  | Q4 | 332 | 47656.331 | 6.967 | 3.63 (2.90-4.55) | <0.001 | 2.05 (1.62-2.60) | <0.001 | 1.70 (1.30-2.22) | <0.001 |
|  | *P* for trend |  |  |  |  | <0.001 |  | <0.001 |  | <0.001 |
| TG/HDL-C | Continuous |  |  |  | 1.00 (1.00-1.00) | 0.036 | 1.00 (1.00-1.00) | 0.283 | 1.00 (1.00-1.01) | 0.627 |
|  | Per 1-SD |  |  |  | 1.03 (1.00-1.05) | 0.036 | 1.02 (0.98-1.05) | 0.283 | 1.01 (0.97-1.06) | 0.627 |
|  | Q1 | 109 | 51926.454 | 2.099 | Reference | — | Reference | — | Reference | — |
|  | Q2 | 130 | 49271.689 | 2.638 | 1.24 (0.96-1.59) | 0.103 | 1.00 (0.77-1.29) | 0.993 | 0.97 (0.75-1.26) | 0.834 |
|  | Q3 | 204 | 48355.794 | 4.219 | 1.97 (1.56-2.48) | <0.001 | 1.35 (1.06-1.71) | 0.014 | 1.27 (1.00-1.61) | 0.049 |
|  | Q4 | 310 | 48239.062 | 6.426 | 2.99 (2.41-3.72) | <0.001 | 1.73 (1.38-2.18) | <0.001 | 1.62 (1.25-2.11) | <0.001 |
|  | *P* for trend |  |  |  |  | <0.001 |  | <0.001 |  | <0.001 |
| METS-IR | Continuous |  |  |  | 1.00 (1.00-1.00) | <0.001 | 1.00 (1.00-1.00) | <0.001 | 1.00 (1.00-1.00) | 0.006 |
|  | Per 1-SD |  |  |  | 1.04 (1.03-1.05) | <0.001 | 1.03 (1.02-1.05) | <0.001 | 1.03 (1.01-1.04) | 0.006 |
|  | Q1 | 93 | 50084.513 | 1.857 | Reference | — | Reference | — | Reference | — |
|  | Q2 | 116 | 50410.344 | 2.301 | 1.25 (0.95-1.64) | 0.115 | 0.92 (0.70-1.22) | 0.569 | 0.91 (0.69-1.20) | 0.516 |
|  | Q3 | 206 | 49118.249 | 4.194 | 2.26 (1.77-2.88) | <0.001 | 1.32 (1.02-1.71) | 0.035 | 1.22 (0.94-1.59) | 0.131 |
|  | Q4 | 338 | 48179.894 | 7.015 | 3.76 (2.99-4.74) | <0.001 | 1.87 (1.45-2.41) | <0.001 | 1.53 (1.16-2.01) | 0.002 |
|  | *P* for trend |  |  |  |  | <0.001 |  | <0.001 |  | <0.001 |
| TyG-BMI | Continuous |  |  |  | 1.02 (1.02-1.02) | <0.001 | 1.01 (1.01-1.01) | <0.001 | 1.01 (1.01-1.01) | <0.001 |
|  | Per 1-SD |  |  |  | 1.77 (1.65-1.90) | <0.001 | 1.42 (1.31-1.55) | <0.001 | 1.29 (1.17-1.41) | <0.001 |
|  | Q1 | 89 | 51049.271 | 1.743 | Reference | — | Reference | — | Reference | — |
|  | Q2 | 112 | 49905.464 | 2.244 | 1.28 (0.97-1.69) | 0.082 | 0.95 (0.71-1.25) | 0.697 | 0.93 (0.70-1.23) | 0.603 |
|  | Q3 | 210 | 48962.892 | 4.289 | 2.44 (1.90-3.12) | <0.001 | 1.42 (1.09-1.84) | 0.008 | 1.34 (1.03-1.74) | 0.032 |
|  | Q4 | 342 | 47875.373 | 7.144 | 4.03 (3.19-5.09) | <0.001 | 2.01 (1.56-2.59) | <0.001 | 1.62 (1.23-2.12) | <0.001 |
|  | *P* for trend |  |  |  |  | <0.001 |  | <0.001 |  | <0.001 |
| TyG-WC | Continuous |  |  |  | 1.00 (1.00-1.00) | <0.001 | 1.00 (1.00-1.00) | <0.001 | 1.00 (1.00-1.00) | <0.001 |
|  | Per 1-SD |  |  |  | 1.12 (1.11-1.14) | <0.001 | 1.11 (1.09-1.14) | <0.001 | 1.09 (1.05-1.12) | <0.001 |
|  | Q1 | 88 | 51230.885 | 1.718 | Reference | — | Reference | — | Reference | — |
|  | Q2 | 100 | 50059.780 | 1.998 | 1.15 (0.87-1.54) | 0.325 | 0.84 (0.63-1.13) | 0.248 | 0.84 (0.63-1.13) | 0.251 |
|  | Q3 | 186 | 49040.945 | 3.793 | 2.19 (1.70-2.82) | <0.001 | 1.19 (0.90-1.58) | 0.226 | 1.14 (0.85-1.52) | 0.383 |
|  | Q4 | 379 | 47461.389 | 7.985 | 4.56 (3.62-5.75) | <0.001 | 1.99 (1.50-2.65) | <0.001 | 1.61 (1.19-2.19) | 0.002 |
|  | *P* for trend |  |  |  |  | <0.001 |  | <0.001 |  | <0.001 |
| TyG-WHtR | Continuous |  |  |  | 1.17 (1.15-1.20) | <0.001 | 1.17 (1.13-1.20) | <0.001 | 1.13 (1.08-1.18) | <0.001 |
|  | Per 1-SD |  |  |  | 1.10 (1.09-1.12) | <0.001 | 1.10 (1.08-1.12) | <0.001 | 1.08 (1.05-1.11) | <0.001 |
|  | Q1 | 91 | 51182.703 | 1.778 | Reference | — | Reference | — | Reference | — |
|  | Q2 | 107 | 50130.859 | 2.134 | 1.19 (0.90-1.57) | 0.221 | 0.88 (0.66-1.17) | 0.368 | 0.89 (0.67-1.18) | 0.419 |
|  | Q3 | 191 | 48933.657 | 3.903 | 2.17 (1.69-2.78) | <0.001 | 1.25 (0.96-1.63) | 0.098 | 1.24 (0.94-1.62) | 0.127 |
|  | Q4 | 364 | 47545.780 | 7.656 | 4.23 (3.36-5.32) | <0.001 | 1.97 (1.52-2.56) | <0.001 | 1.65 (1.24-2.19) | <0.001 |
|  | *P* for trend |  |  |  |  | <0.001 |  | <0.001 |  | <0.001 |
| METS-VF | Continuous |  |  |  | 1.56 (1.50-1.62) | <0.001 | 1.37 (1.23-1.52) | <0.001 | 1.25 (1.10-1.42) | <0.001 |
|  | Per 1-SD |  |  |  | 1.43 (1.39-1.48) | <0.001 | 1.29 (1.18-1.40) | <0.001 | 1.19 (1.08-1.32) | <0.001 |
|  | Q1 | 82 | 49866.495 | 1.644 | Reference | — | Reference | — | Reference | — |
|  | Q2 | 107 | 50674.191 | 2.112 | 1.30 (0.97-1.73) | 0.078 | 0.99 (0.74-1.33) | 0.960 | 1.02 (0.76-1.36) | 0.921 |
|  | Q3 | 184 | 50007.471 | 3.679 | 2.26 (1.74-2.93) | <0.001 | 1.26 (0.94-1.69) | 0.119 | 1.24 (0.92-1.67) | 0.159 |
|  | Q4 | 380 | 47244.843 | 8.043 | 4.86 (3.83-6.17) | <0.001 | 1.93 (1.40-2.64) | <0.001 | 1.61 (1.15-2.24) | 0.005 |
|  | *P* for trend |  |  |  |  | <0.001 |  | <0.001 |  | <0.001 |
| LAP | Continuous |  |  |  | 1.00 (1.00-1.01) | <0.001 | 1.00 (1.00-1.00) | <0.001 | 1.00 (1.00-1.00) | 0.005 |
|  | Per 1-SD |  |  |  | 1.15 (1.12-1.18) | <0.001 | 1.11 (1.07-1.15) | <0.001 | 1.08 (1.02-1.13) | 0.005 |
|  | Q1 | 101 | 51702.878 | 1.953 | Reference | — | Reference | — | Reference | — |
|  | Q2 | 129 | 49916.409 | 2.584 | 1.31 (1.01-1.70) | 0.044 | 0.99 (0.76-1.29) | 0.938 | 0.98 (0.75-1.27) | 0.859 |
|  | Q3 | 199 | 48342.155 | 4.116 | 2.06 (1.62-2.62) | <0.001 | 1.28 (1.00-1.64) | 0.051 | 1.19 (0.93-1.53) | 0.172 |
|  | Q4 | 324 | 47831.559 | 6.774 | 3.38 (2.71-4.23) | <0.001 | 1.77 (1.39-2.24) | <0.001 | 1.55 (1.19-2.01) | 0.001 |
|  | *P* for trend |  |  |  |  | <0.001 |  | <0.001 |  | <0.001 |
| CVAI | Continuous |  |  |  | 1.01 (1.01-1.01) | <0.001 | 1.01 (1.00-1.01) | <0.001 | 1.00 (1.00-1.01) | <0.001 |
|  | Per 1-SD |  |  |  | 1.44 (1.40-1.49) | <0.001 | 1.31 (1.22-1.41) | <0.001 | 1.21 (1.11-1.33) | <0.001 |
|  | Q1 | 84 | 50299.987 | 1.670 | Reference | — | Reference | — | Reference | — |
|  | Q2 | 115 | 50951.862 | 2.257 | 1.36 (1.03-1.80) | 0.032 | 0.93 (0.69-1.25) | 0.633 | 0.95 (0.71-1.28) | 0.749 |
|  | Q3 | 177 | 49583.193 | 3.570 | 2.14 (1.65-2.78) | <0.001 | 1.03 (0.76-1.39) | 0.855 | 1.01 (0.74-1.37) | 0.972 |
|  | Q4 | 377 | 46957.959 | 8.028 | 4.75 (3.75-6.02) | <0.001 | 1.70 (1.24-2.32) | <0.001 | 1.42 (1.02-1.98) | 0.038 |
|  | *P* for trend |  |  |  |  | <0.001 |  | <0.001 |  | 0.004 |
| AIP | Continuous |  |  |  | 3.57 (2.93-4.36) | <0.001 | 2.15 (1.71-2.72) | <0.001 | 2.07 (1.53-2.80) | <0.001 |
|  | Per 1-SD |  |  |  | 1.44 (1.36-1.53) | <0.001 | 1.25 (1.17-1.33) | <0.001 | 1.23 (1.13-1.34) | <0.001 |
|  | Q1 | 109 | 51926.454 | 2.099 | Reference | — | Reference | — | Reference | — |
|  | Q2 | 130 | 49271.689 | 2.638 | 1.24 (0.96-1.59) | 0.103 | 1.00 (0.77-1.29) | 0.993 | 0.97 (0.75-1.26) | 0.834 |
|  | Q3 | 204 | 48355.794 | 4.219 | 1.97 (1.56-2.48) | <0.001 | 1.35 (1.06-1.71) | 0.014 | 1.27 (1.00-1.61) | 0.049 |
|  | Q4 | 310 | 48239.062 | 6.426 | 2.99 (2.41-3.72) | <0.001 | 1.73 (1.38-2.18) | <0.001 | 1.62 (1.25-2.11) | <0.001 |
|  | *P* for trend |  |  |  |  | <0.001 |  | <0.001 |  | <0.001 |
| CHG | Continuous |  |  |  | 3.49 (3.11-3.90) | <0.001 | 2.65 (2.28-3.07) | <0.001 | 2.41 (1.99-2.92) | <0.001 |
|  | Per 1-SD |  |  |  | 1.56 (1.50-1.62) | <0.001 | 1.41 (1.34-1.49) | <0.001 | 1.37 (1.28-1.46) | <0.001 |
|  | Q1 | 86 | 50188.712 | 1.714 | Reference | — | Reference | — | Reference | — |
|  | Q2 | 126 | 49948.644 | 2.523 | 1.48 (1.13-1.95) | 0.005 | 1.11 (0.84-1.46) | 0.474 | 1.14 (0.86-1.51) | 0.360 |
|  | Q3 | 186 | 49787.364 | 3.736 | 2.19 (1.70-2.83) | <0.001 | 1.30 (1.00-1.70) | 0.054 | 1.33 (1.01-1.74) | 0.040 |
|  | Q4 | 355 | 47868.280 | 7.416 | 4.33 (3.42-5.47) | <0.001 | 2.12 (1.64-2.74) | <0.001 | 1.91 (1.43-2.54) | <0.001 |
|  | *P* for trend |  |  |  |  | <0.001 |  | <0.001 |  | <0.001 |
| RC | Continuous |  |  |  | 1.01 (1.01-1.01) | <0.001 | 1.01 (1.00-1.01) | <0.001 | 1.01 (1.00-1.01) | <0.001 |
|  | Per 1-SD |  |  |  | 1.17 (1.13-1.20) | <0.001 | 1.13 (1.08-1.18) | <0.001 | 1.12 (1.07-1.17) | <0.001 |
|  | Q1 | 112 | 44914.220 | 2.494 | Reference | — | Reference | — | Reference | — |
|  | Q2 | 159 | 48723.933 | 3.263 | 1.35 (1.06-1.72) | 0.014 | 1.25 (0.98-1.60) | 0.067 | 1.31 (1.03-1.67) | 0.029 |
|  | Q3 | 202 | 51972.090 | 3.887 | 1.64 (1.30-2.07) | <0.001 | 1.34 (1.06-1.69) | 0.013 | 1.38 (1.09-1.75) | 0.006 |
|  | Q4 | 280 | 52182.757 | 5.366 | 2.31 (1.86-2.88) | <0.001 | 1.63 (1.30-2.03) | <0.001 | 1.62 (1.27-2.05) | <0.001 |
|  | *P* for trend |  |  |  |  | <0.001 |  | <0.001 |  | <0.001 |

*Notes:* After excluding 7,110 participants with obesity (BMI ≥28 kg/m²), 41,194 participants remained in the analysis, of whom 753 developed incident CKD. Incidence rates are presented per 1,000 person-years. Model 1 was unadjusted; Model 2 was adjusted for age and sex; Model 3 was further adjusted for smoking status, alcohol consumption, medication use, diabetes, dyslipidemia, hypertension, and eGFR.

Abbreviations: AIP, atherogenic index of plasma; BMI, body mass index; CHG, cholesterol–high-density lipoprotein–glucose index; CI, confidence interval; CKD, chronic kidney disease; CVAI, Chinese visceral adiposity index; eGFR, estimated glomerular filtration rate; HR, hazard ratio; LAP, lipid accumulation product; METS-IR, metabolic score for insulin resistance; METS-VF, metabolic score for visceral fat; PY, person-years; RC, remnant cholesterol; SD, standard deviation; TG/HDL-C, triglyceride to high-density lipoprotein cholesterol ratio; TyG, triglyceride–glucose index; TyG-BMI, triglyceride–glucose index combined with body mass index; TyG-WC, triglyceride–glucose index combined with waist circumference; TyG-WHtR, triglyceride–glucose index combined with waist-to-height ratio

Supplementary Table 13. Associations of surrogate indices of insulin resistance with progression to RKFD in participants with CKD after excluding those with BMI ≥28 kg/m²

| Exposure | Levels | Model 1 | | Model 2 | | Model 3 | |
| --- | --- | --- | --- | --- | --- | --- | --- |
|  |  | OR(95%CI) | *P* | OR(95%CI) | *P* | OR(95%CI) | *P* |
| TyG | Continuous | 1.96 (1.20-3.19) | 0.007 | 1.83 (1.09-3.05) | 0.021 | 1.86 (1.12-3.10) | 0.017 |
|  | Per 1-SD | 1.55 (1.13-2.13) | 0.007 | 1.48 (1.06-2.07) | 0.021 | 1.50 (1.07-2.09) | 0.017 |
| TG/HDL-C | Continuous | 1.03 (0.97-1.09) | 0.321 | 1.03 (0.97-1.09) | 0.418 | 1.03 (0.97-1.09) | 0.355 |
|  | Per 1-SD | 1.13 (0.89-1.43) | 0.321 | 1.11 (0.86-1.42) | 0.418 | 1.13 (0.87-1.45) | 0.355 |
| METS-IR | Continuous | 1.04 (0.99-1.09) | 0.091 | 1.03 (0.98-1.09) | 0.212 | 1.04 (0.98-1.09) | 0.180 |
|  | Per 1-SD | 1.32 (0.96-1.81) | 0.091 | 1.25 (0.88-1.78) | 0.212 | 1.28 (0.89-1.83) | 0.180 |
| TyG-BMI | Continuous | 1.01 (1.00-1.02) | 0.206 | 1.00 (0.99-1.02) | 0.470 | 1.00 (0.99-1.02) | 0.466 |
|  | Per 1-SD | 1.26 (0.88-1.82) | 0.206 | 1.16 (0.78-1.71) | 0.470 | 1.16 (0.78-1.72) | 0.466 |
| TyG-WC | Continuous | 1.00 (1.00-1.01) | 0.064 | 1.00 (1.00-1.01) | 0.231 | 1.00 (1.00-1.01) | 0.213 |
|  | Per 1-SD | 1.42 (0.98-2.05) | 0.064 | 1.30 (0.85-2.00) | 0.231 | 1.32 (0.85-2.04) | 0.213 |
| TyG-WHtR | Continuous | 1.61 (0.90-2.86) | 0.107 | 1.38 (0.72-2.68) | 0.335 | 1.42 (0.73-2.76) | 0.304 |
|  | Per 1-SD | 1.34 (0.94-1.92) | 0.107 | 1.22 (0.81-1.84) | 0.335 | 1.24 (0.82-1.88) | 0.304 |
| METS-VF | Continuous | 1.16 (0.69-1.94) | 0.583 | 0.71 (0.36-1.43) | 0.339 | 0.71 (0.35-1.43) | 0.339 |
|  | Per 1-SD | 1.11 (0.77-1.61) | 0.583 | 0.79 (0.48-1.29) | 0.339 | 0.78 (0.48-1.29) | 0.339 |
| LAP | Continuous | 1.00 (0.99-1.01) | 0.550 | 1.00 (0.99-1.01) | 0.841 | 1.00 (0.99-1.01) | 0.842 |
|  | Per 1-SD | 1.10 (0.80-1.52) | 0.550 | 1.04 (0.74-1.46) | 0.841 | 1.04 (0.73-1.46) | 0.842 |
| CVAI | Continuous | 1.01 (1.00-1.01) | 0.210 | 1.00 (0.99-1.01) | 0.841 | 1.00 (0.99-1.01) | 0.852 |
|  | Per 1-SD | 1.27 (0.87-1.84) | 0.210 | 1.05 (0.64-1.73) | 0.841 | 1.05 (0.63-1.73) | 0.852 |
| AIP | Continuous | 2.41 (0.79-7.32) | 0.120 | 2.06 (0.64-6.70) | 0.228 | 2.16 (0.65-7.11) | 0.206 |
|  | Per 1-SD | 1.30 (0.93-1.81) | 0.120 | 1.24 (0.87-1.77) | 0.228 | 1.26 (0.88-1.80) | 0.206 |
| CHG | Continuous | 2.73 (1.55-4.80) | <0.001 | 2.57 (1.43-4.64) | 0.002 | 2.83 (1.54-5.18) | <0.001 |
|  | Per 1-SD | 1.61 (1.23-2.09) | <0.001 | 1.56 (1.18-2.06) | 0.002 | 1.63 (1.23-2.17) | <0.001 |
| RC | Continuous | 1.00 (0.99-1.02) | 0.505 | 1.00 (0.99-1.02) | 0.580 | 1.00 (0.99-1.02) | 0.513 |
|  | Per 1-SD | 1.11 (0.82-1.49) | 0.505 | 1.09 (0.80-1.48) | 0.580 | 1.11 (0.81-1.51) | 0.513 |

*Notes:* After excluding participants with BMI ≥28 kg/m², the analysis was restricted to participants who developed incident CKD, among whom 33 subsequently progressed to RKFD. Model 1 was unadjusted; Model 2 was adjusted for age and sex; Model 3 was further adjusted for eGFR.

Abbreviations: AIP, atherogenic index of plasma; BMI, body mass index; CHG, cholesterol–high-density lipoprotein–glucose index; CI, confidence interval; CKD, chronic kidney disease; CVAI, Chinese visceral adiposity index; eGFR, estimated glomerular filtration rate; LAP, lipid accumulation product; METS-IR, metabolic score for insulin resistance; METS-VF, metabolic score for visceral fat; OR, odds ratio; RC, remnant cholesterol; RKFD, rapid kidney function decline; SD, standard deviation; TG/HDL-C, triglyceride-to-high-density lipoprotein cholesterol ratio; TyG, triglyceride–glucose index; TyG-BMI, triglyceride–glucose index combined with body mass index; TyG-WC, triglyceride–glucose index combined with waist circumference; TyG-WHtR, triglyceride–glucose index combined with waist-to-height ratio

Supplementary Table 14. Associations of surrogate indices of insulin resistance with progression to surrogate endpoints in participants with incident CKD after excluding those with BMI ≥28 kg/m²

| Exposure | Levels | HR(95%CI) | *P* |
| --- | --- | --- | --- |
| TyG | Continuous | 2.78 (1.28-6.06) | 0.01 |
|  | Per 1-SD | 1.99 (1.18-3.37) | 0.01 |
| TG/HDL-C | Continuous | 1.05 (0.98-1.12) | 0.161 |
|  | Per 1-SD | 1.26 (0.91-1.75) | 0.161 |
| METS-IR | Continuous | 1.08 (1.01-1.15) | 0.029 |
|  | Per 1-SD | 1.70 (1.06-2.72) | 0.029 |
| TyG-BMI | Continuous | 1.03 (1.00-1.06) | 0.036 |
|  | Per 1-SD | 2.67 (1.16-6.13) | 0.036 |
| TyG-WC | Continuous | 1.01 (1.00-1.02) | 0.021 |
|  | Per 1-SD | 2.54 (1.06-6.09) | 0.021 |
| TyG-WHtR | Continuous | 3.63 (1.11-11.91) | 0.034 |
|  | Per 1-SD | 2.31 (1.07-4.99) | 0.034 |
| METS-VF | Continuous | 3.38 (0.64-17.93) | 0.152 |
|  | Per 1-SD | 2.42 (0.72-8.11) | 0.152 |
| LAP | Continuous | 1.01 (0.99-1.03) | 0.335 |
|  | Per 1-SD | 1.31 (0.76-2.26) | 0.335 |
| CVAI | Continuous | 1.02 (1.00-1.04) | 0.1 |
|  | Per 1-SD | 2.37 (0.85-6.65) | 0.1 |
| AIP | Continuous | 5.17 (0.80-33.35) | 0.084 |
|  | Per 1-SD | 1.68 (0.93-3.03) | 0.084 |
| CHG | Continuous | 4.89 (2.15-11.10) | <0.001 |
|  | Per 1-SD | 2.20 (1.46-3.29) | <0.001 |
| RC | Continuous | 1.01 (1.00-1.03) | 0.078 |
|  | Per 1-SD | 1.32 (0.97-1.81) | 0.078 |

*Note:* After excluding participants with BMI ≥28 kg/m², the analysis was restricted to participants who developed CKD, among whom 8 subsequently progressed to the surrogate endpoints. Given the limited number of events, no covariate adjustment was performed to minimize the risk of model overfitting. Surrogate endpoints were defined as either a 40% decline in eGFR or a doubling of serum creatinine.

Abbreviations: AIP, atherogenic index of plasma; BMI, body mass index; CHG, cholesterol–high-density lipoprotein–glucose index; CI, confidence interval; CKD, chronic kidney disease; CVAI, Chinese visceral adiposity index; eGFR, estimated glomerular filtration rate; HR, hazard ratio; LAP, lipid accumulation product; METS-IR, metabolic score for insulin resistance; METS-VF, metabolic score for visceral fat; RC, remnant cholesterol; SD, standard deviation; TG/HDL-C, triglyceride to high-density lipoprotein cholesterol ratio; TyG, triglyceride–glucose index; TyG-BMI, triglyceride–glucose index combined with body mass index; TyG-WC, triglyceride–glucose index combined with waist circumference; TyG-WHtR, triglyceride–glucose index combined with waist-to-height ratio

Supplementary Table 15. Discriminative performance and prediction error of surrogate indices of insulin resistance for incident CKD

| Model | Harrell’s-C-index | Uno-C-index | IBS |
| --- | --- | --- | --- |
| TyG | 0.748 (0.736–0.756) | 0.728 (0.728–0.729) | 0.00726 |
| TG/HDL-C | 0.740 (0.739–0.742) | 0.722 (0.721–0.723) | 0.00728 |
| METS-IR | 0.734 (0.732–0.736) | 0.723 (0.722–0.733) | 0.00728 |
| TyG-BMI | 0.741 (0.734–0.750) | 0.723 (0.721–0.729) | 0.00728 |
| TyG-WC | 0.735 (0.730–0.744) | 0.729 (0.726–0.727) | 0.00728 |
| TyG-WHtR | 0.738 (0.725–0.752) | 0.727 (0.727–0.734) | 0.00728 |
| METS-VF | 0.739 (0.732–0.745) | 0.730 (0.730–0.734) | 0.00728 |
| LAP | 0.738 (0.730–0.743) | 0.725 (0.724–0.725) | 0.00728 |
| CVAI | 0.730 (0.726–0.733) | 0.724 (0.721–0.732) | 0.00728 |
| AIP | 0.746 (0.744–0.747) | 0.728 (0.726–0.728) | 0.00727 |
| CHG | 0.750 (0.748–0.751) | 0.728 (0.728–0.729) | 0.00723 |
| RC | 0.745 (0.741–0.748) | 0.723 (0.721–0.723) | 0.00727 |

*Note:* Models were adjusted for age, sex, smoking status, alcohol consumption, medication use, diabetes, dyslipidemia, hypertension, and eGFR.

Abbreviations: AIP, atherogenic index of plasma; AUC, area under the curve; CHG, cholesterol–high-density lipoprotein–glucose index; CKD, chronic kidney disease; CVAI, Chinese visceral adiposity index; C-index, concordance index; eGFR, estimated glomerular filtration rate; IBS, integrated Brier score; LAP, lipid accumulation product; METS-IR, metabolic score for insulin resistance; METS-VF, metabolic score for visceral fat; RC, remnant cholesterol; TG/HDL-C, triglyceride to high-density lipoprotein cholesterol ratio; TyG, triglyceride–glucose index; TyG-BMI, triglyceride–glucose index combined with body mass index; TyG-WC, triglyceride–glucose index combined with waist circumference; TyG-WHtR, triglyceride–glucose index combined with waist-to-height ratio

Supplementary Figure 1. Directed acyclic graph illustrating the assumed relationships between surrogate indices of insulin resistance and chronic kidney disease.

*Note:* The green node with a black triangle represents the exposure, i.e., surrogate indices of insulin resistance. The blue node with a black vertical bar represents the outcome, chronic kidney disease. Light green nodes represent ancestors of the exposure, blue nodes represent ancestors of the outcome, and pink nodes represent common ancestors of both the exposure and the outcome. Directed arrows denote assumed direct relationships between variables. Green arrows indicate paths from the exposure to the outcome. Blue arrows indicate paths from ancestors of the outcome to the outcome. Pink arrows indicate paths involving common ancestors of both the exposure and outcome, representing potential confounding structures

Abbreviations: eGFR, estimated glomerular filtration rate

Supplementary Figure 2. Pairwise Spearman correlations among the 12 surrogate insulin resistance indices.

Abbreviations: AIP, atherogenic index of plasma; CHG, cholesterol–high-density lipoprotein–glucose index; CVAI, Chinese visceral adiposity index; LAP, lipid accumulation product; METS-IR, metabolic score for insulin resistance; METS-VF, metabolic score for visceral fat; RC, remnant cholesterol; TG/HDL-C, triglyceride to high-density lipoprotein cholesterol ratio; TyG, triglyceride–glucose index; TyG-BMI, triglyceride–glucose index combined with body mass index; TyG-WC, triglyceride–glucose index combined with waist circumference; TyG-WHtR, triglyceride–glucose index combined with waist-to-height ratio

Supplementary Figure 3. Restricted cubic spline analyses of associations between surrogate indices of insulin resistance and RKFD in participants with CKD: (A) TyG; (B) TG/HDL-C; (C) METS-IR; (D) TyG-BMI; (E) TyG-WC; (F) TyG-WHtR; (G) METS-VF; (H) LAP; (I) CVAI; (J) AIP; (K) CHG; (L) RC.

*Notes:* Restricted cubic spline analyses were conducted using logistic regression models and adjusted for age, sex, and eGFR.

Abbreviations: AIP, atherogenic index of plasma; CHG, cholesterol–high-density lipoprotein–glucose index; CI, confidence interval; CKD, chronic kidney disease; CVAI, Chinese visceral adiposity index; eGFR, estimated glomerular filtration rate; LAP, lipid accumulation product; METS-IR, metabolic score for insulin resistance; METS-VF, metabolic score for visceral fat; OR, odds ratio; RC, remnant cholesterol; RKFD, rapid kidney function decline; TG/HDL-C, triglyceride to high-density lipoprotein cholesterol ratio; TyG, triglyceride–glucose index; TyG-BMI, triglyceride–glucose index combined with body mass index; TyG-WC, triglyceride–glucose index combined with waist circumference; TyG-WHtR, triglyceride–glucose index combined with waist-to-height ratio

Supplementary Figure 4. Restricted cubic spline analyses of associations between surrogate indices of insulin resistance and surrogate endpoints in participants with CKD: (A) TyG; (B) TG/HDL-C; (C) METS-IR; (D) TyG-BMI; (E) TyG-WC; (F) TyG-WHtR; (G) METS-VF; (H) LAP; (I) CVAI; (J) AIP; (K) CHG; (L) RC.

*Notes:* Restricted cubic spline models were adjusted for age, sex, and eGFR. Surrogate endpoints were defined as either a 40% decline in eGFR or a doubling of serum creatinine.

Abbreviations: AIP, atherogenic index of plasma; CHG, cholesterol–high-density lipoprotein–glucose index; CI, confidence interval; CKD, chronic kidney disease; CVAI, Chinese visceral adiposity index; eGFR, estimated glomerular filtration rate; HR, hazard ratio; LAP, lipid accumulation product; METS-IR, metabolic score for insulin resistance; METS-VF, metabolic score for visceral fat; RC, remnant cholesterol; TG/HDL-C, triglyceride to high-density lipoprotein cholesterol ratio; TyG, triglyceride–glucose index; TyG-BMI, triglyceride–glucose index combined with body mass index; TyG-WC, triglyceride–glucose index combined with waist circumference; TyG-WHtR, triglyceride–glucose index combined with waist-to-height ratio

Supplementary Figure 5. Subgroup analyses of the association between TyG-WC and incident CKD.

Abbreviations: BMI, body mass index; CI, confidence interval; CKD, chronic kidney disease; HR, hazard ratio; PY, person-years; TyG-WC, triglyceride–glucose index combined with waist circumference

Figure S6. Calibration plots of surrogate indices of insulin resistance for incident CKD at 3 years.

*Note:* Models were adjusted for age, sex, smoking status, alcohol consumption, medication use, diabetes, dyslipidemia, hypertension, and eGFR.

Abbreviations: AIP, atherogenic index of plasma; CHG, cholesterol–high-density lipoprotein–glucose index; CKD, chronic kidney disease; CVAI, Chinese visceral adiposity index; eGFR, estimated glomerular filtration rate; LAP, lipid accumulation product; METS-IR, metabolic score for insulin resistance; METS-VF, metabolic score for visceral fat; RC, remnant cholesterol; TG/HDL-C, triglyceride to high-density lipoprotein cholesterol ratio; TyG, triglyceride–glucose index; TyG-BMI, triglyceride–glucose index combined with body mass index; TyG-WC, triglyceride–glucose index combined with waist circumference; TyG-WHtR, triglyceride–glucose index combined with waist-to-height ratio

Supplementary Figure 7. Decision curve analysis of surrogate indices of insulin resistance for incident CKD at 3 years.

*Note:* Models were adjusted for age, sex, smoking status, alcohol consumption, medication use, diabetes, dyslipidemia, hypertension, and eGFR.

Abbreviations: AIP, atherogenic index of plasma; CHG, cholesterol–high-density lipoprotein–glucose index; CKD, chronic kidney disease; CVAI, Chinese visceral adiposity index; eGFR, estimated glomerular filtration rate; LAP, lipid accumulation product; METS-IR, metabolic score for insulin resistance; METS-VF, metabolic score for visceral fat; RC, remnant cholesterol; TG/HDL-C, triglyceride to high-density lipoprotein cholesterol ratio; TyG, triglyceride–glucose index; TyG-BMI, triglyceride–glucose index combined with body mass index; TyG-WC, triglyceride–glucose index combined with waist circumference; TyG-WHtR, triglyceride–glucose index combined with waist-to-height ratio

Supplementary Figure 8. Time-dependent Brier scores of surrogate indices of insulin resistance for incident CKD.

*Note:* Models were adjusted for age, sex, smoking status, alcohol consumption, medication use, diabetes, dyslipidemia, hypertension, eGFR.

Abbreviations: AIP, atherogenic index of plasma; CHG, cholesterol–high-density lipoprotein–glucose index; CKD, chronic kidney disease; CVAI, Chinese visceral adiposity index; eGFR, estimated glomerular filtration rate; LAP, lipid accumulation product; METS-IR, metabolic score for insulin resistance; METS-VF, metabolic score for visceral fat; RC, remnant cholesterol; TG/HDL-C, triglyceride to high-density lipoprotein cholesterol ratio; TyG, triglyceride–glucose index; TyG-BMI, triglyceride–glucose index combined with body mass index; TyG-WC, triglyceride–glucose index combined with waist circumference; TyG-WHtR, triglyceride–glucose index combined with waist-to-height ratio

Supplementary Figure 9. Kaplan–Meier curves for risk stratification of incident CKD based on TyG-WC–derived median predicted 3-year risk.

Abbreviations: CKD, chronic kidney disease; TyG-WC, triglyceride–glucose index combined with waist circumference

Supplementary Figure 10. Mediation analysis of monocytes in the associations between surrogate indices of insulin resistance and incident CKD.

*Note:* The surrogate indices of insulin resistance were categorized into two groups based on the 75% cut-off. **P* < 0.05, ***P* < 0.01, ****P* < 0.001.

Abbreviations: AIP, atherogenic index of plasma; CHG, cholesterol–high-density lipoprotein–glucose index; CKD, chronic kidney disease; CVAI, Chinese visceral adiposity index; eGFR, estimated glomerular filtration rate; LAP, lipid accumulation product; METS-IR, metabolic score for insulin resistance; METS-VF, metabolic score for visceral fat; RC, remnant cholesterol; TG/HDL-C, triglyceride to high-density lipoprotein cholesterol ratio; TyG, triglyceride–glucose index; TyG-BMI, triglyceride–glucose index combined with body mass index; TyG-WC, triglyceride–glucose index combined with waist circumference; TyG-WHtR, triglyceride–glucose index combined with waist-to-height ratio; β_DE, direct effect; β_IE, indirect effect; β_total, total effect; Prop, mediation proportion
